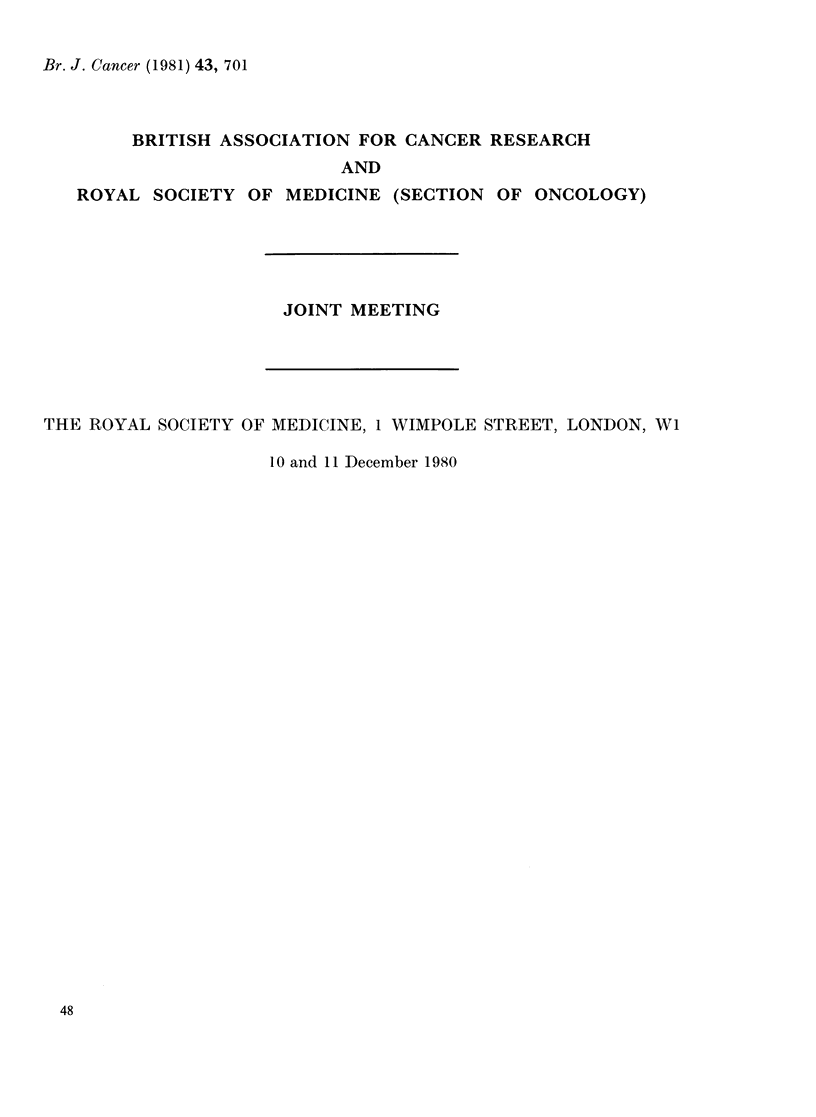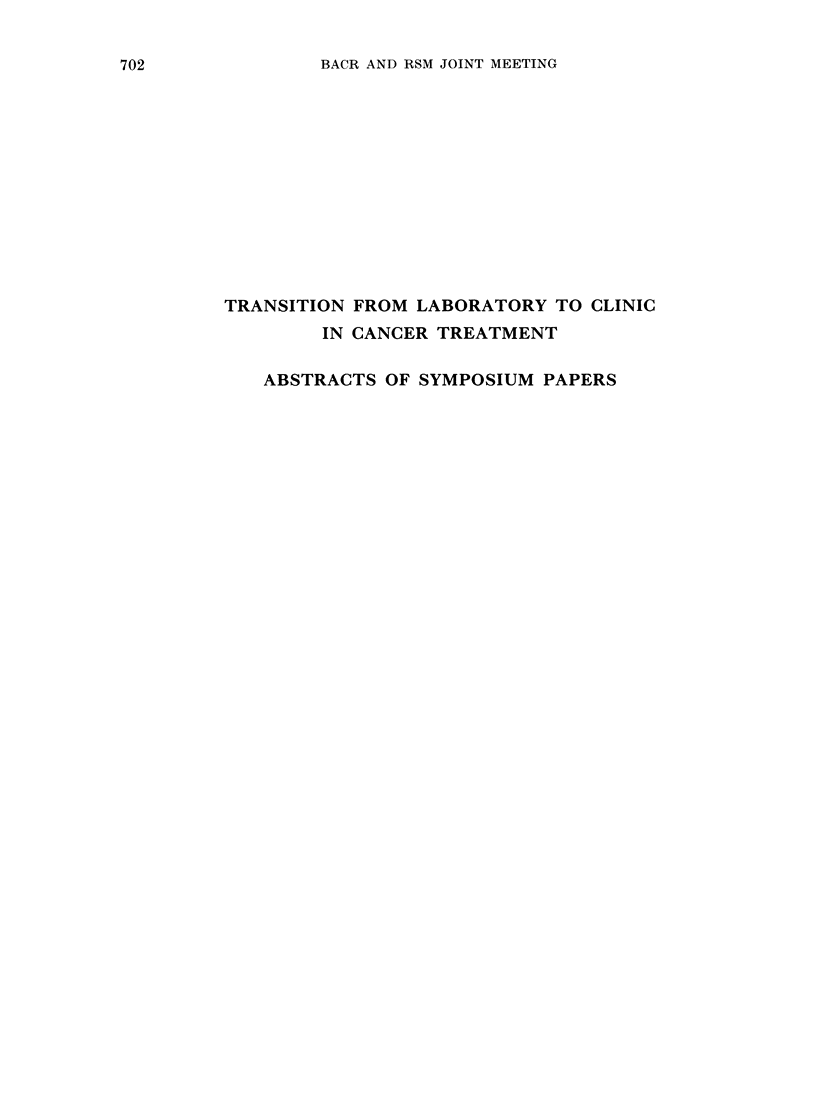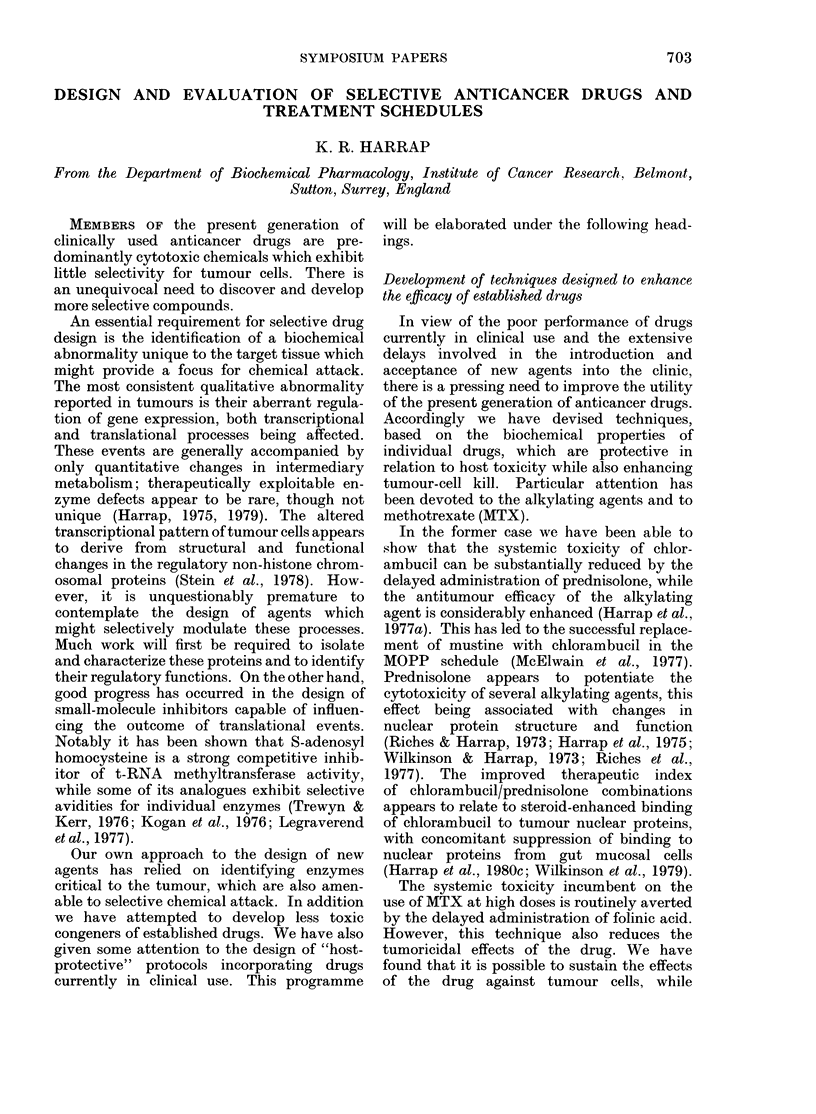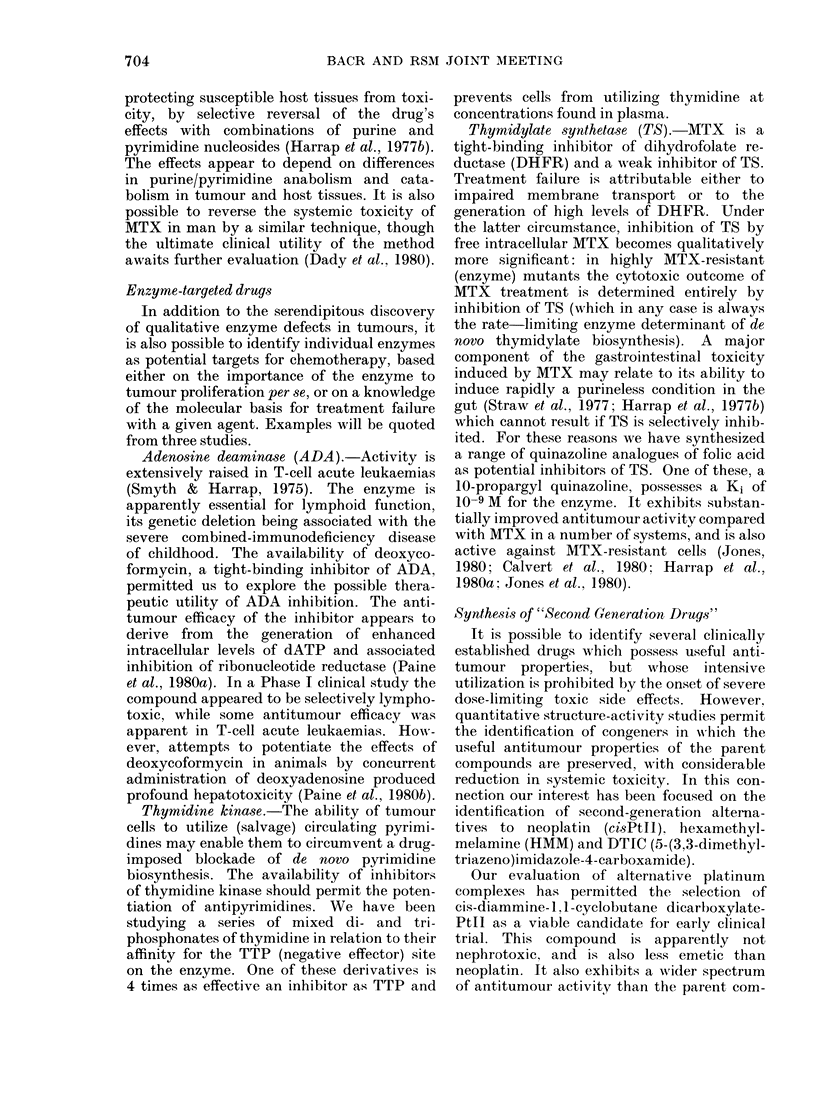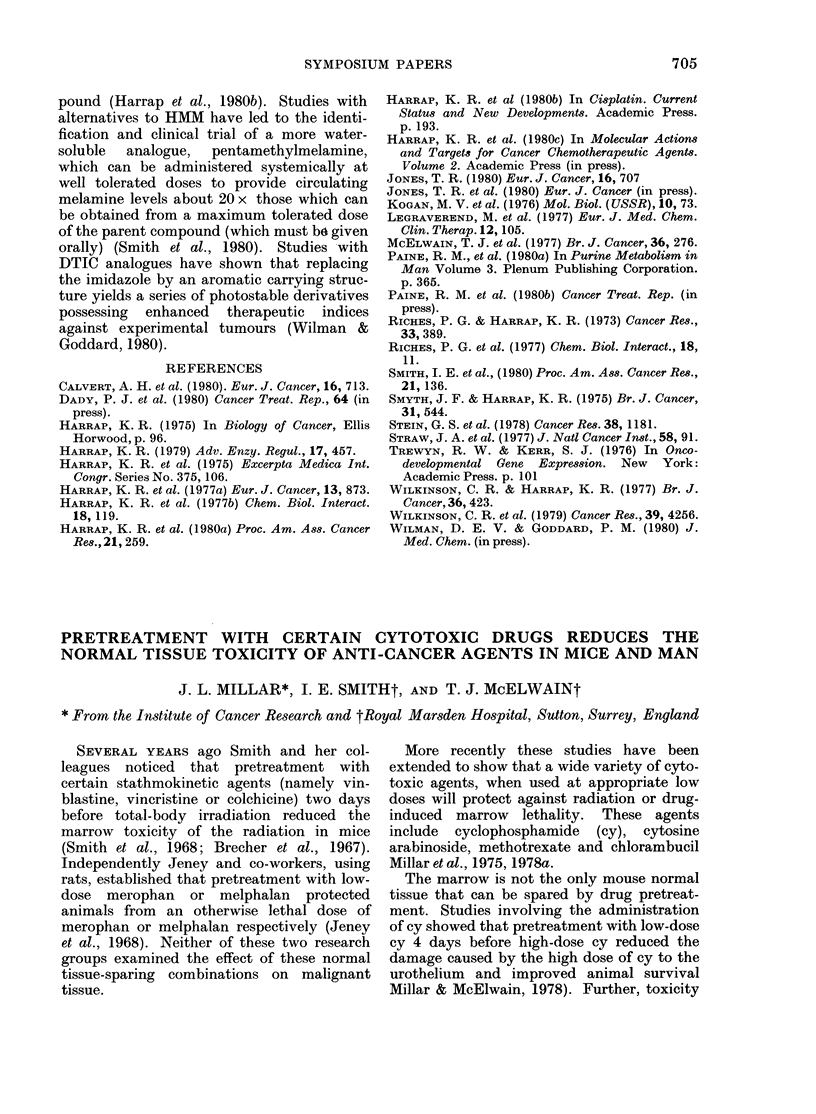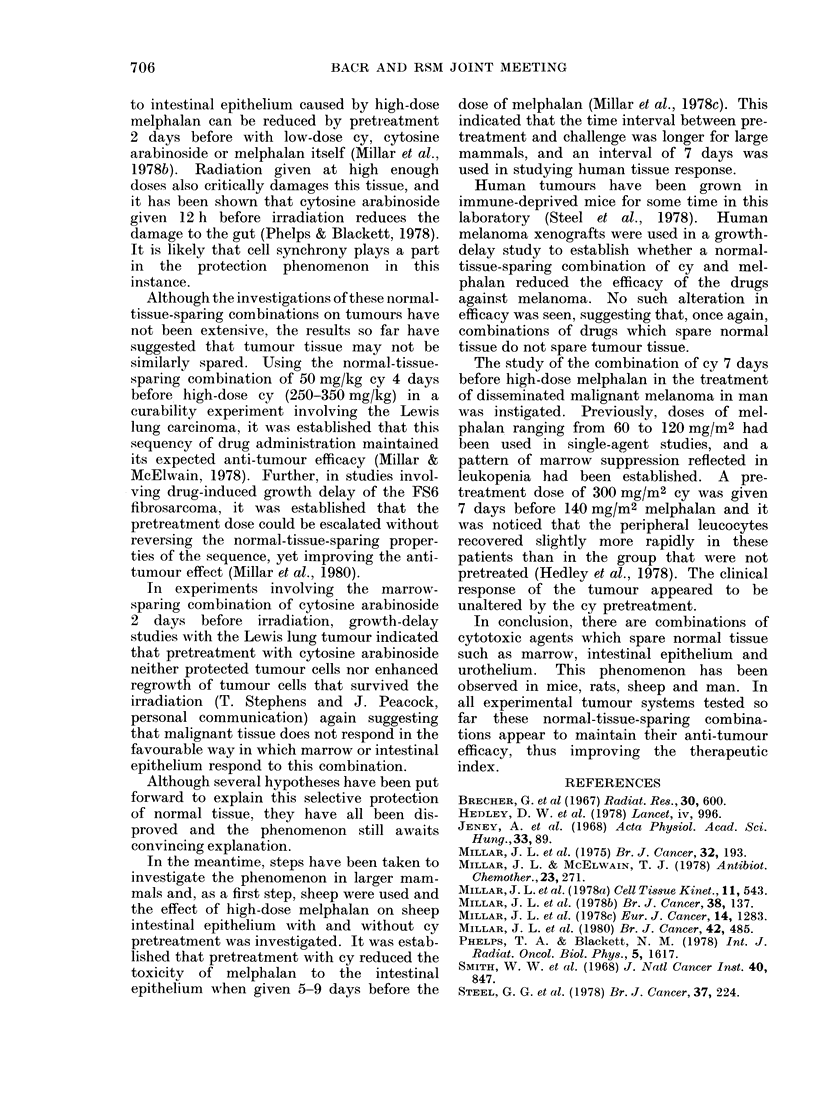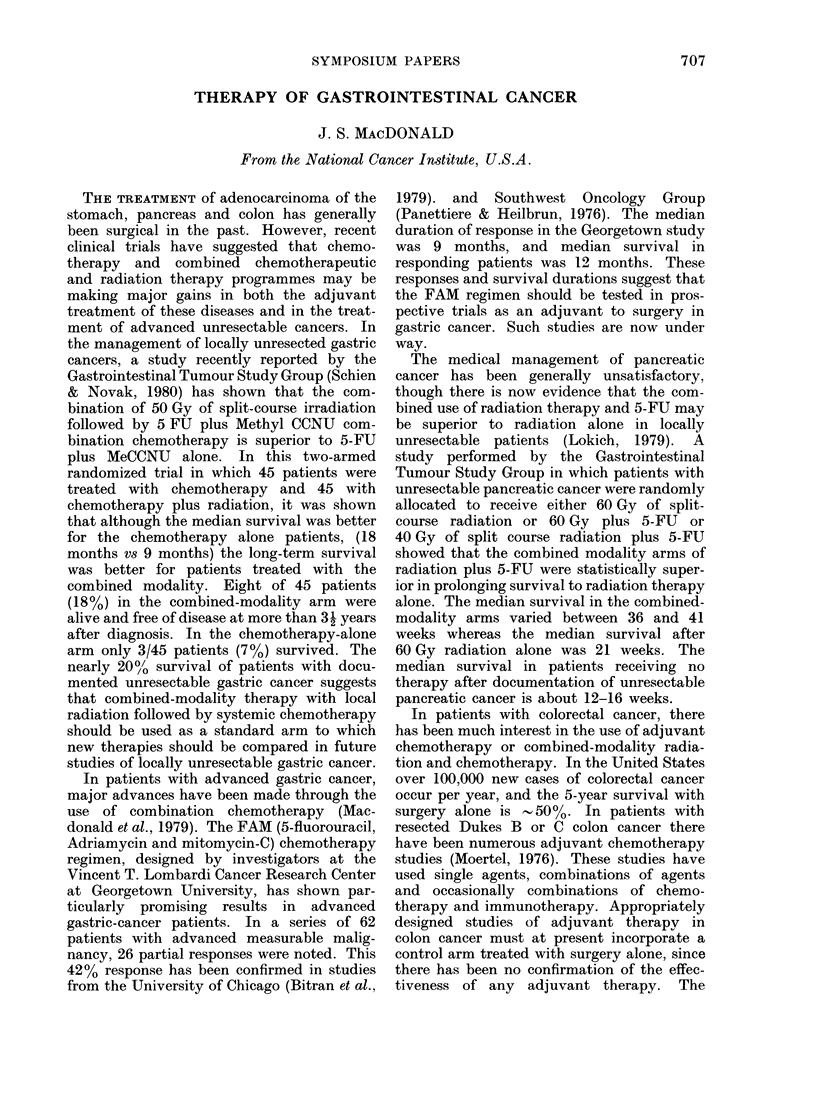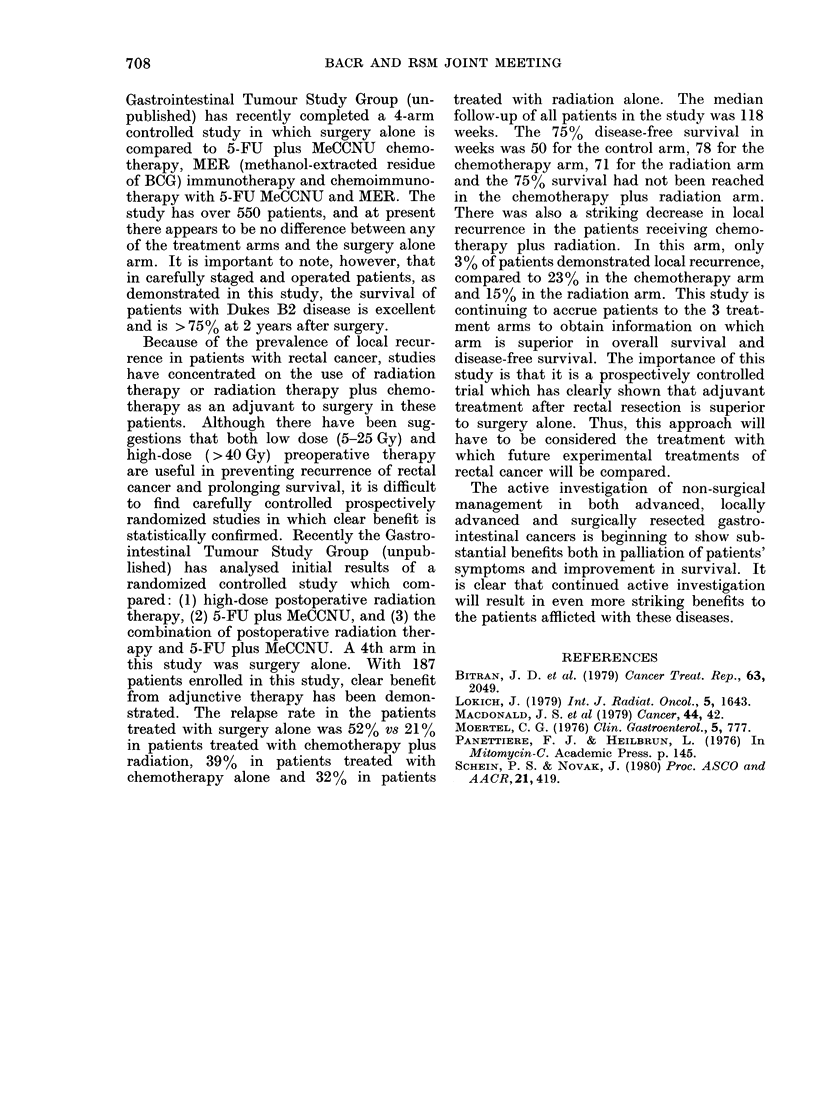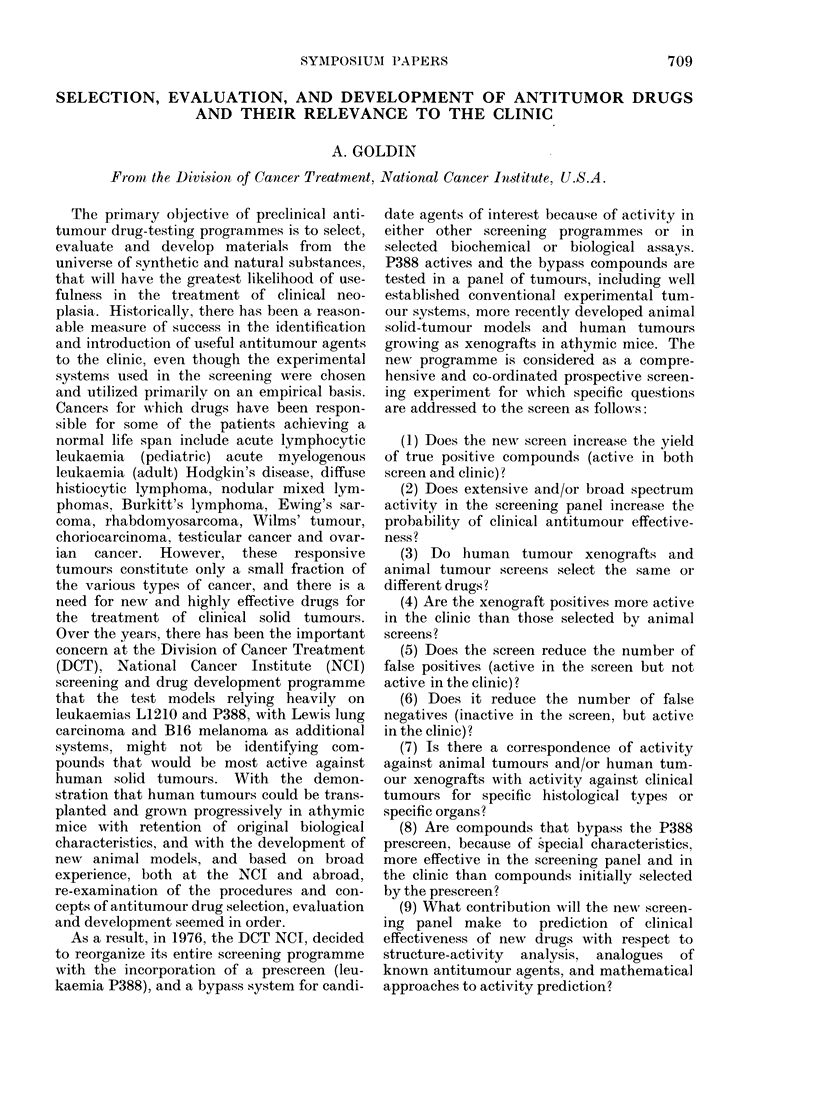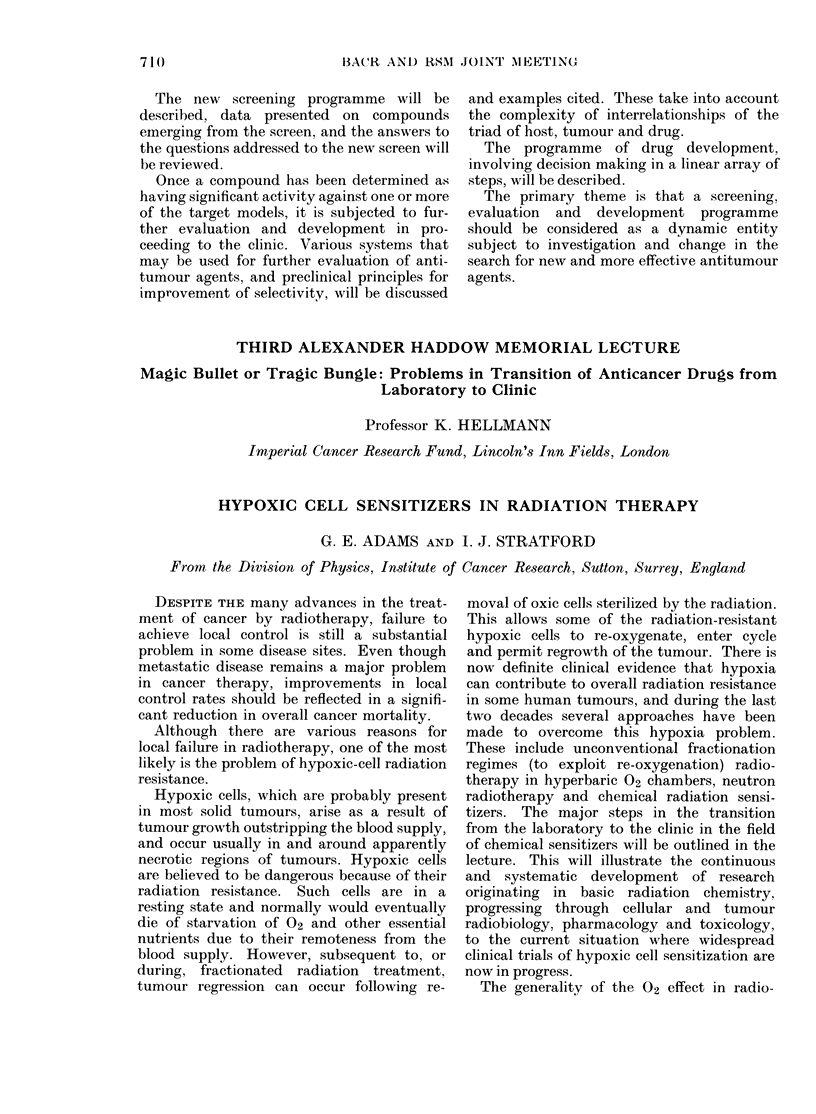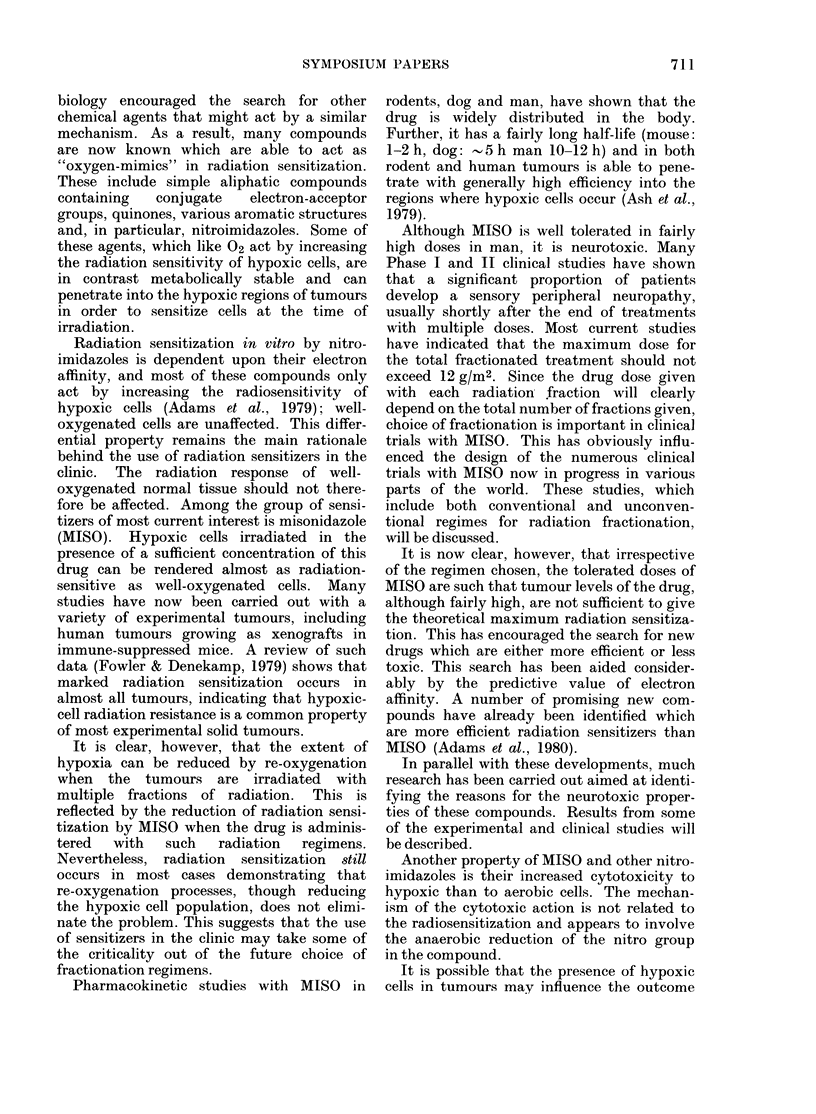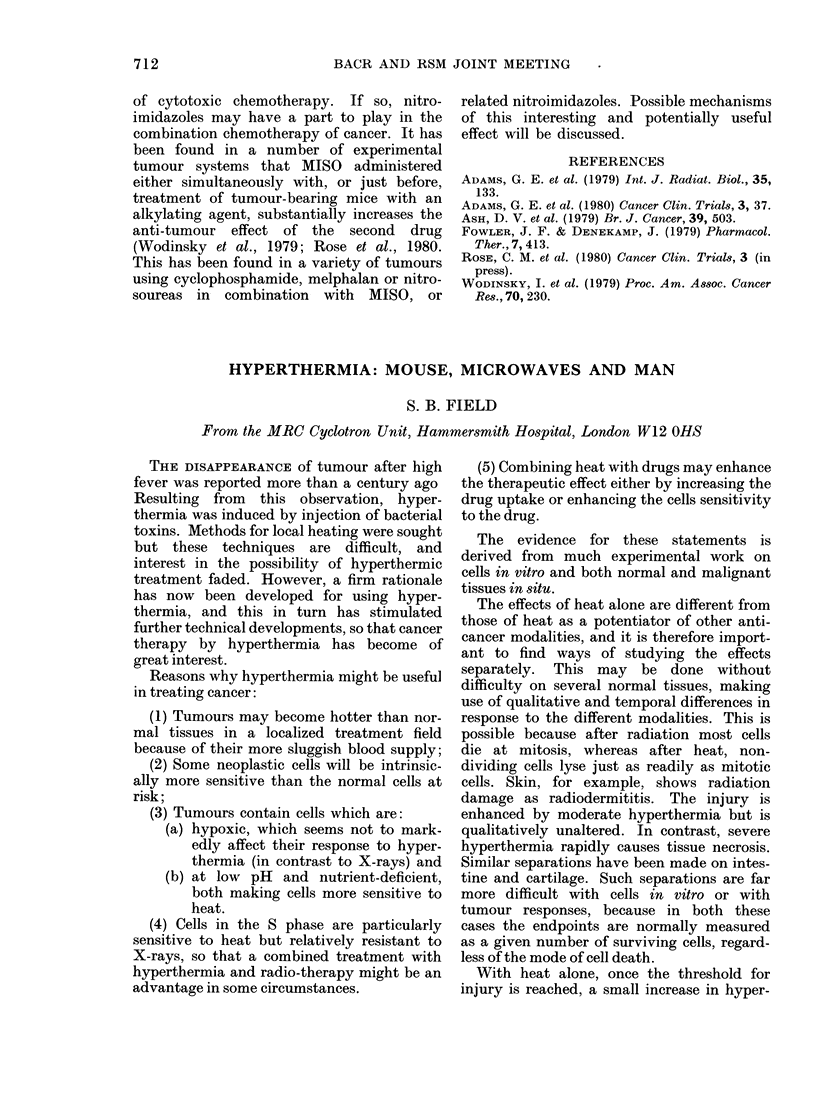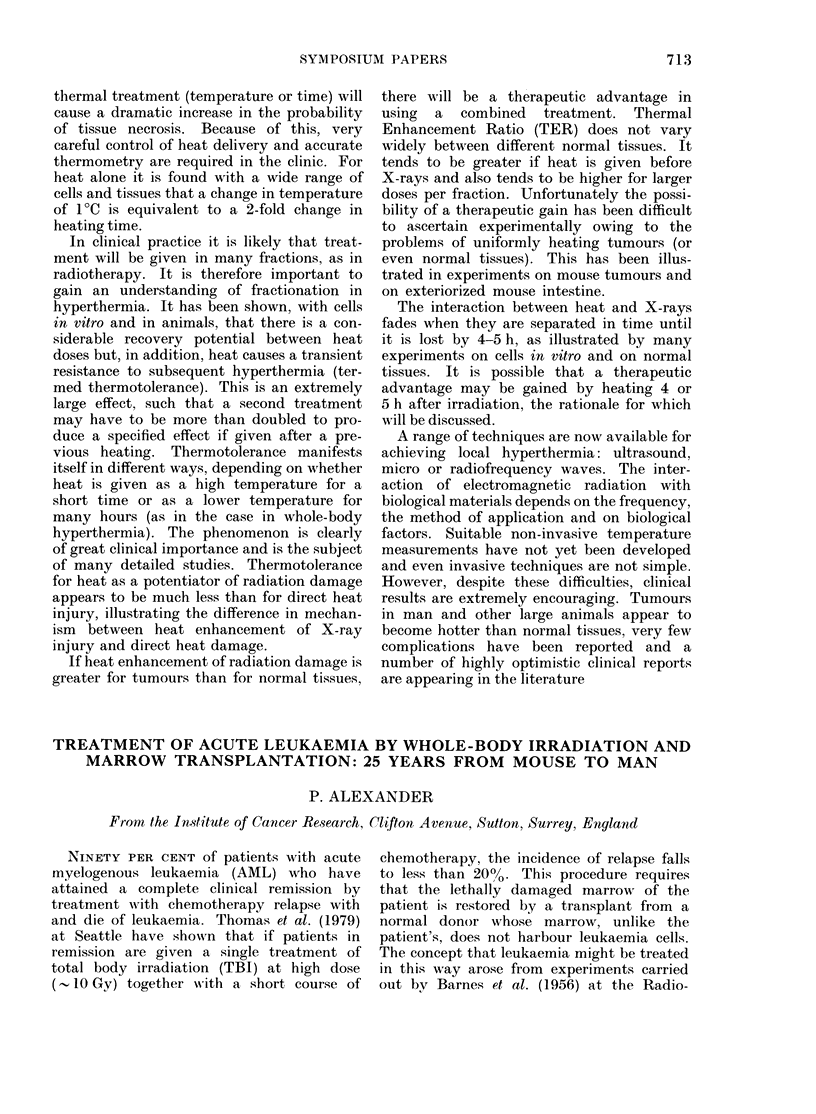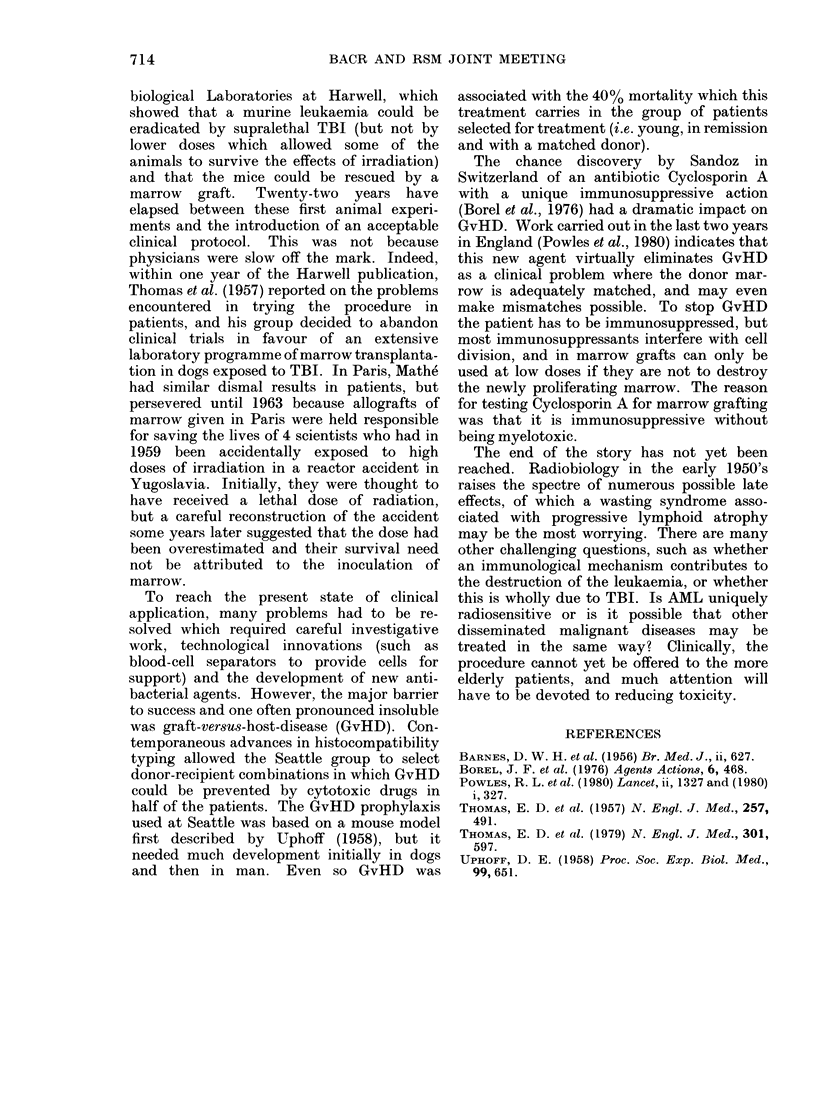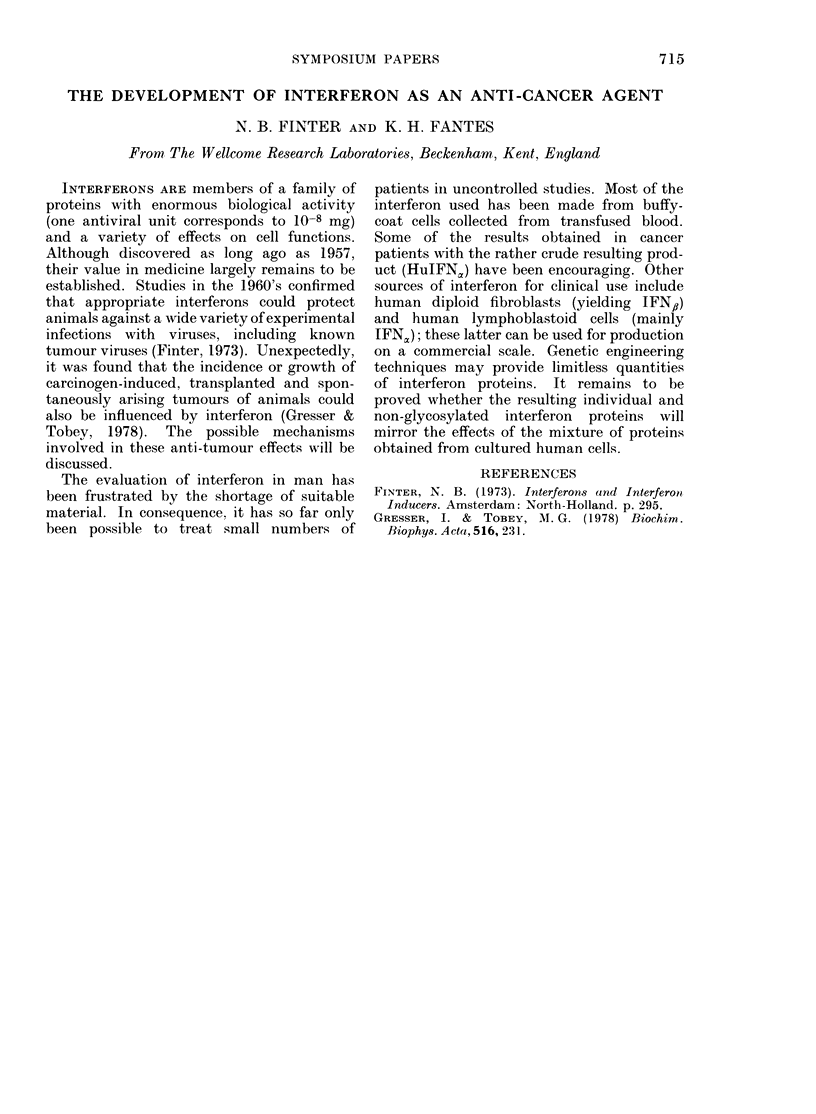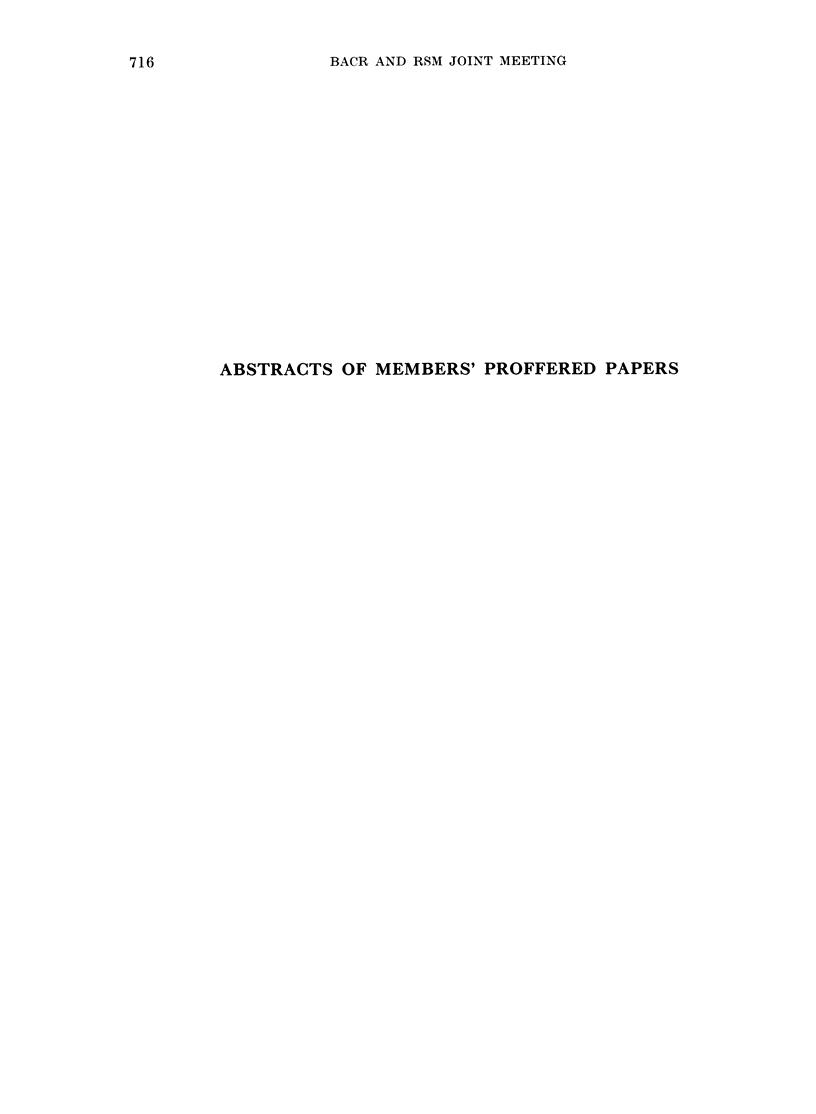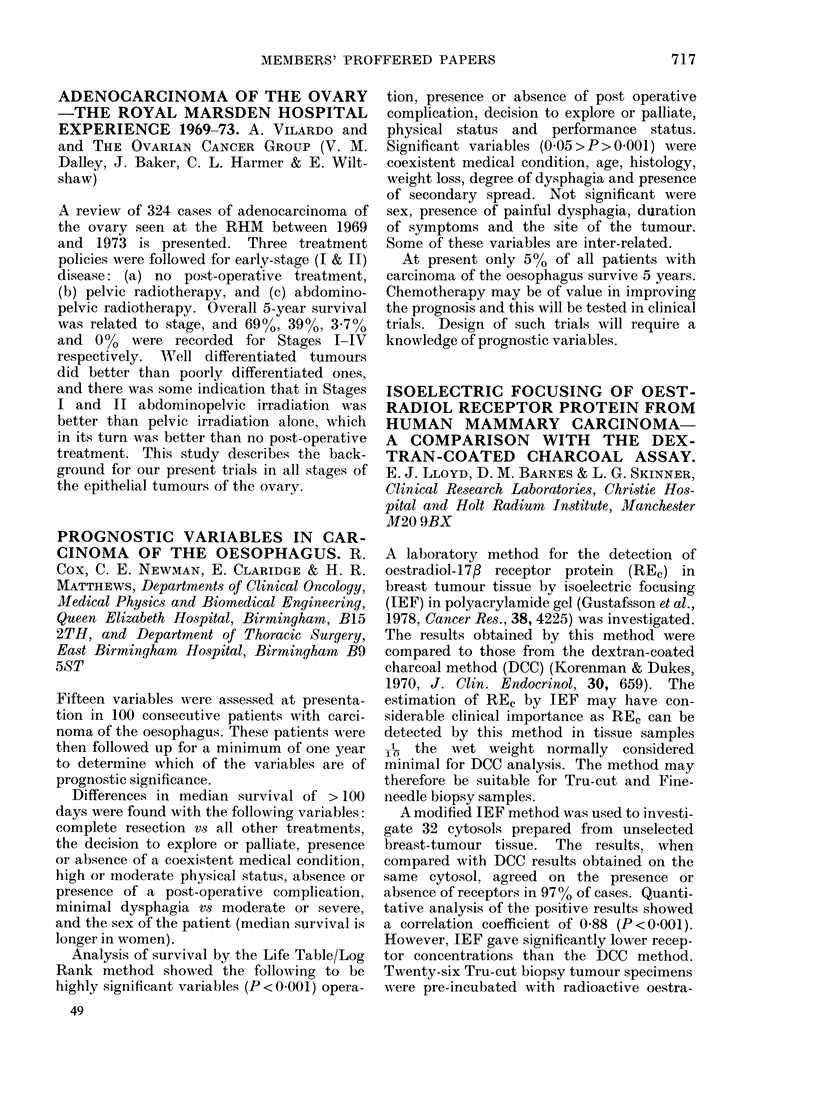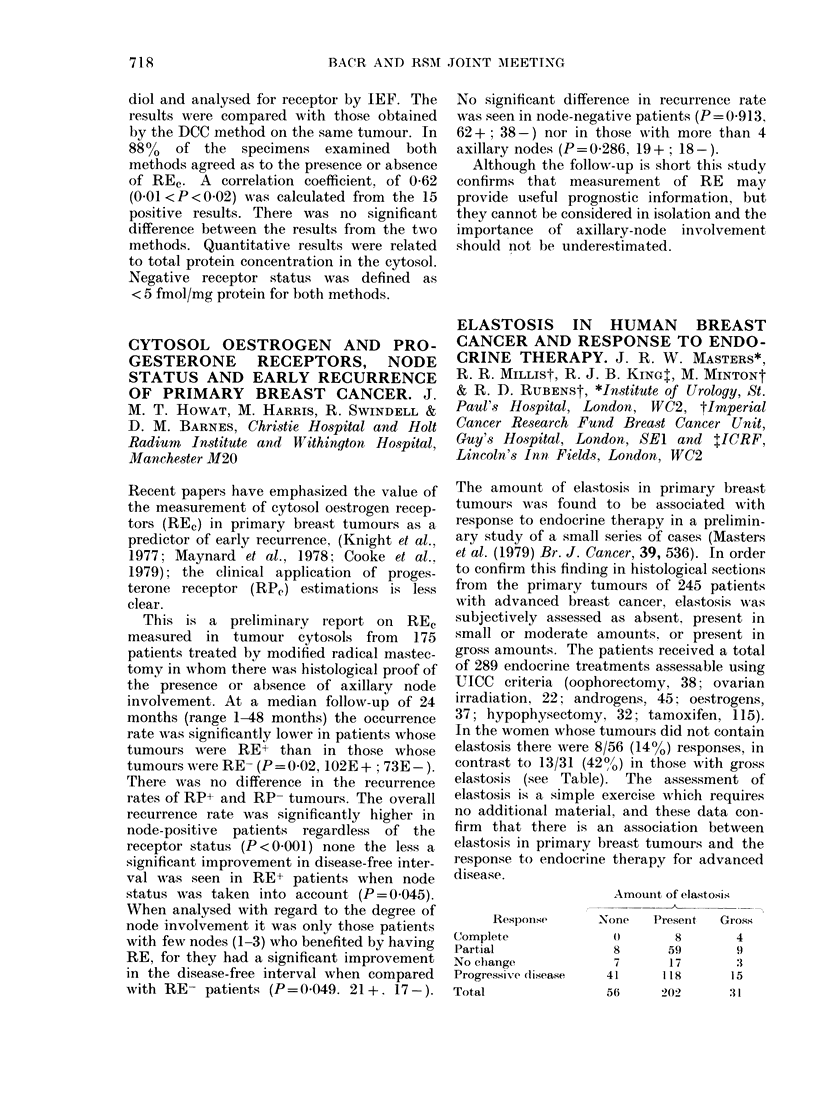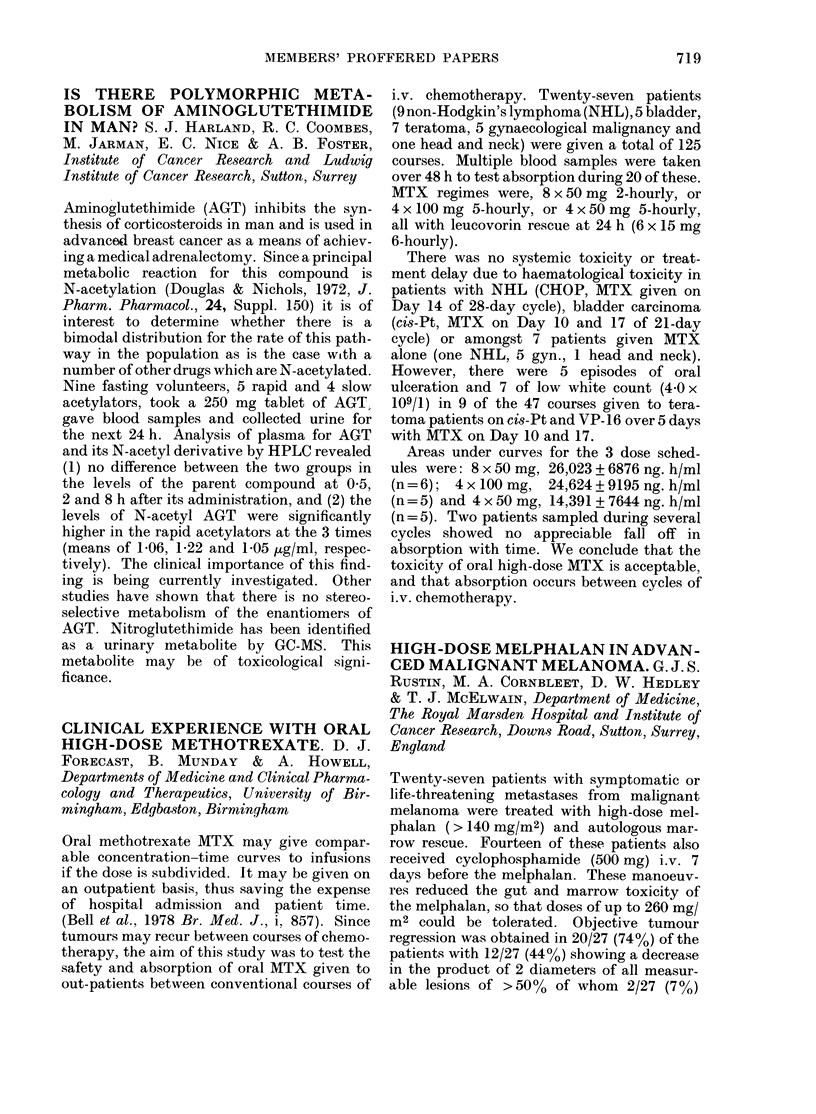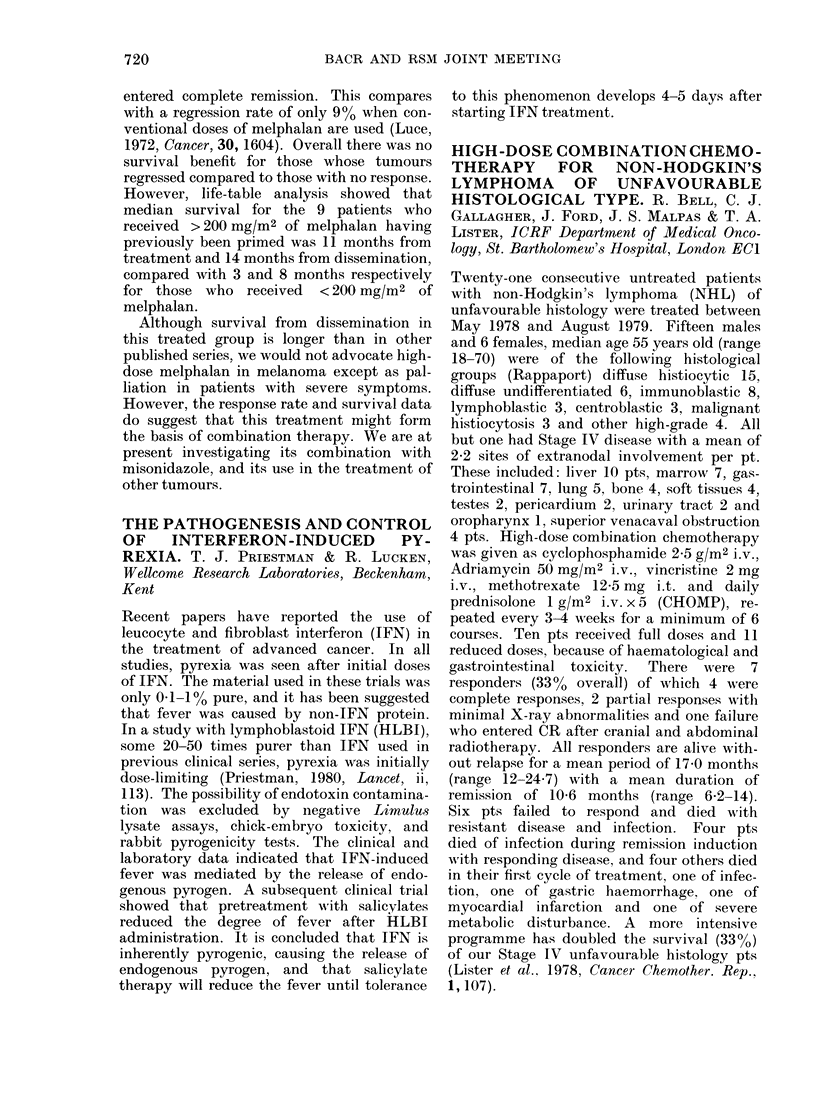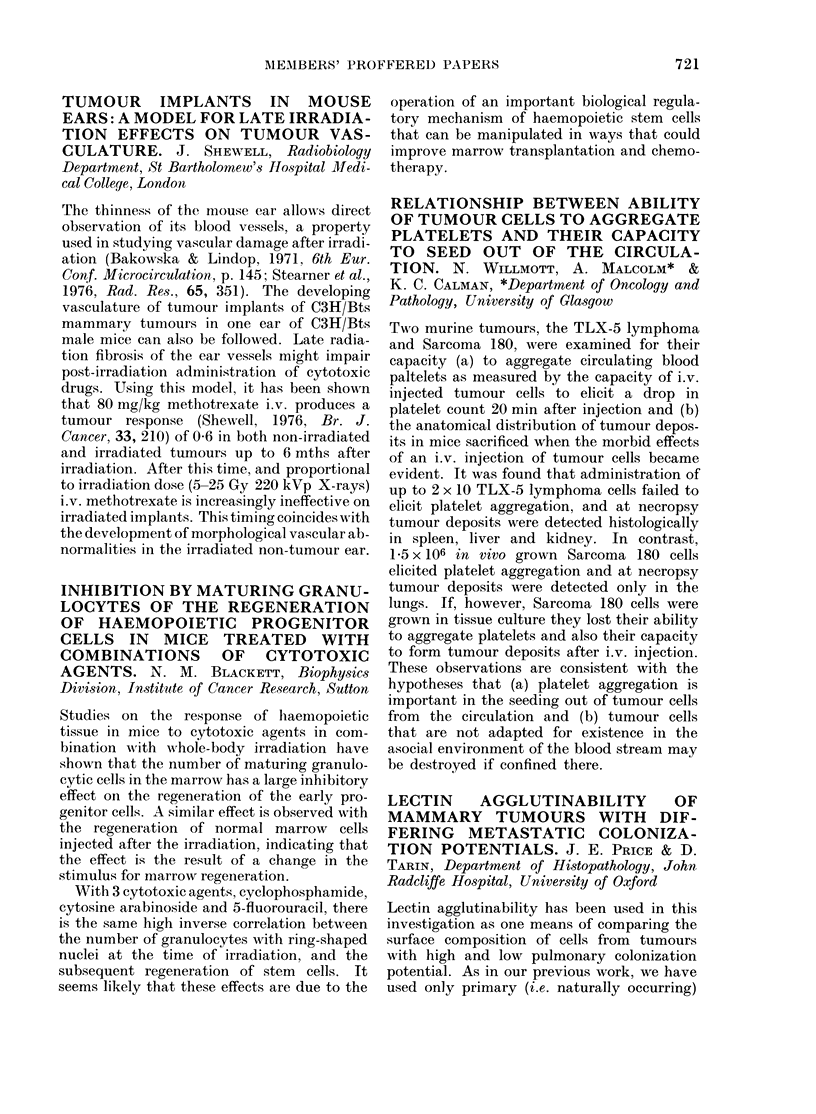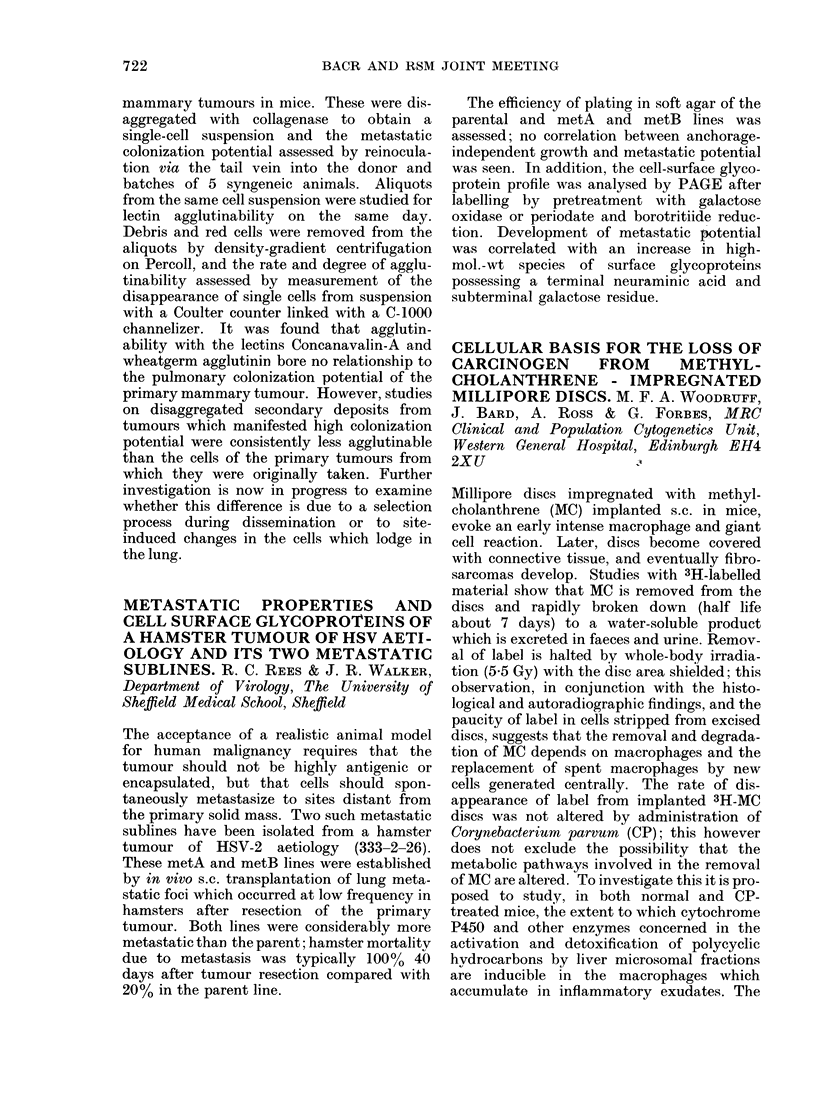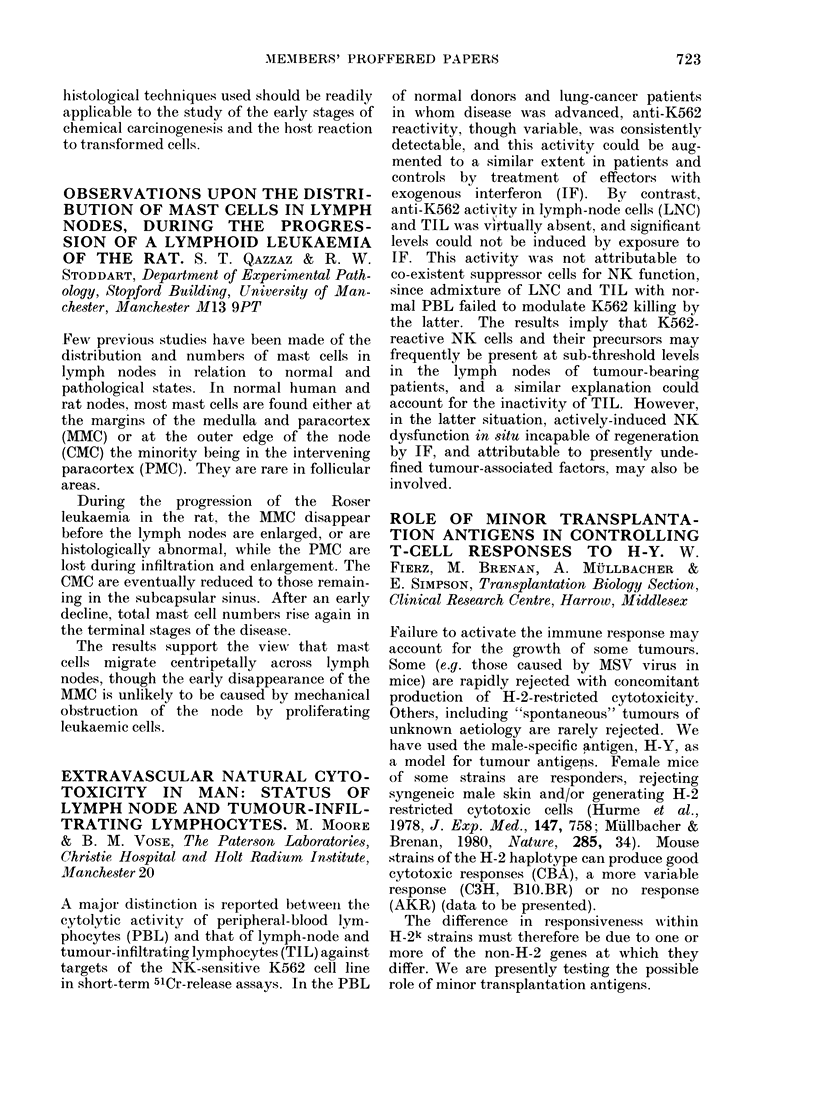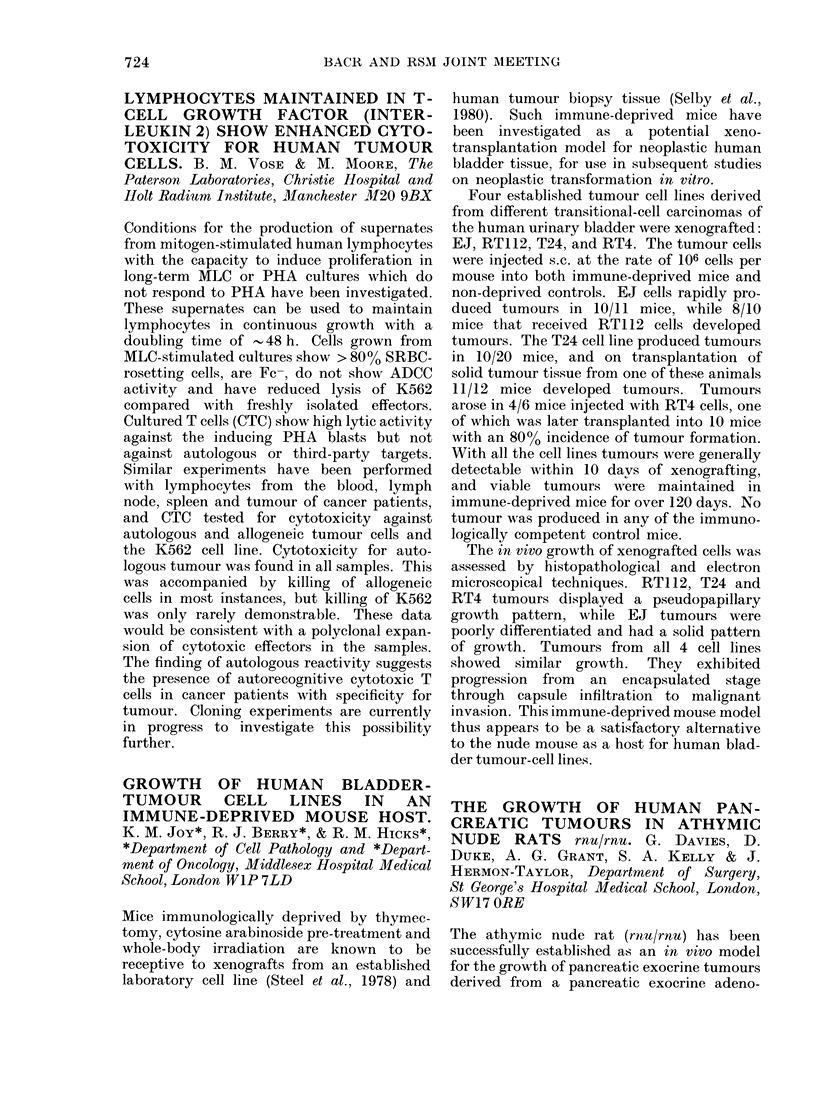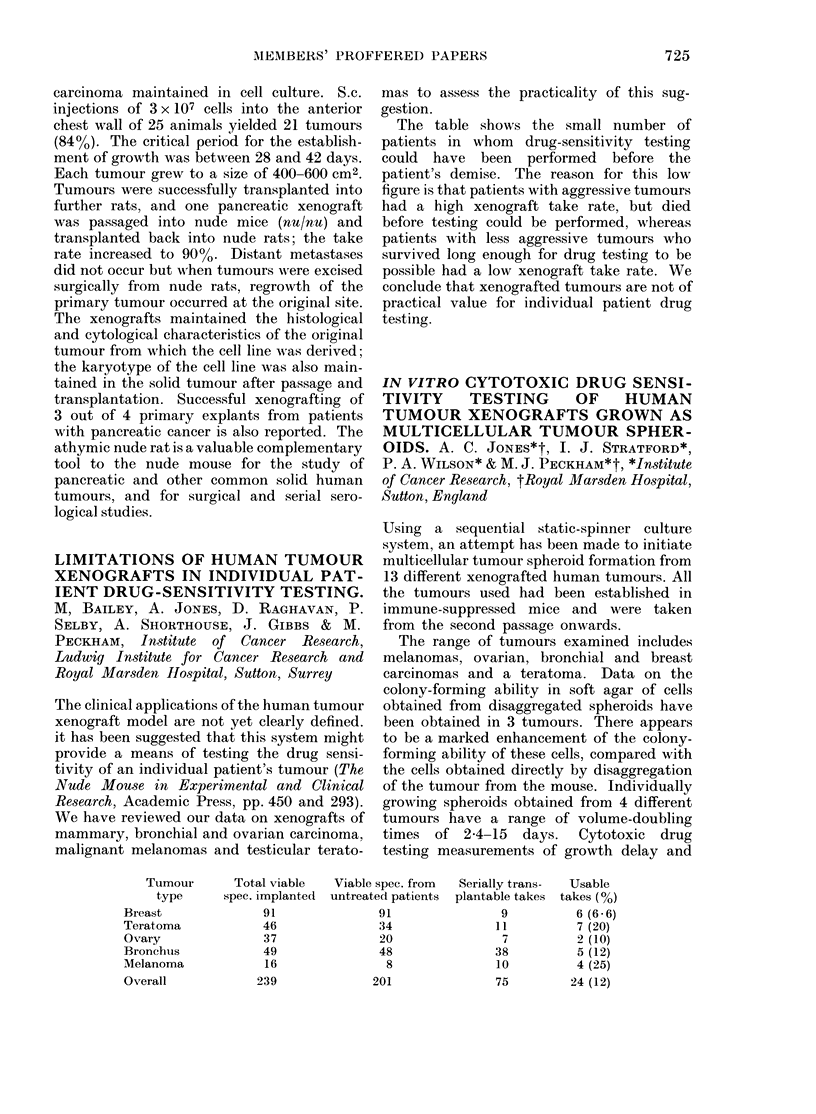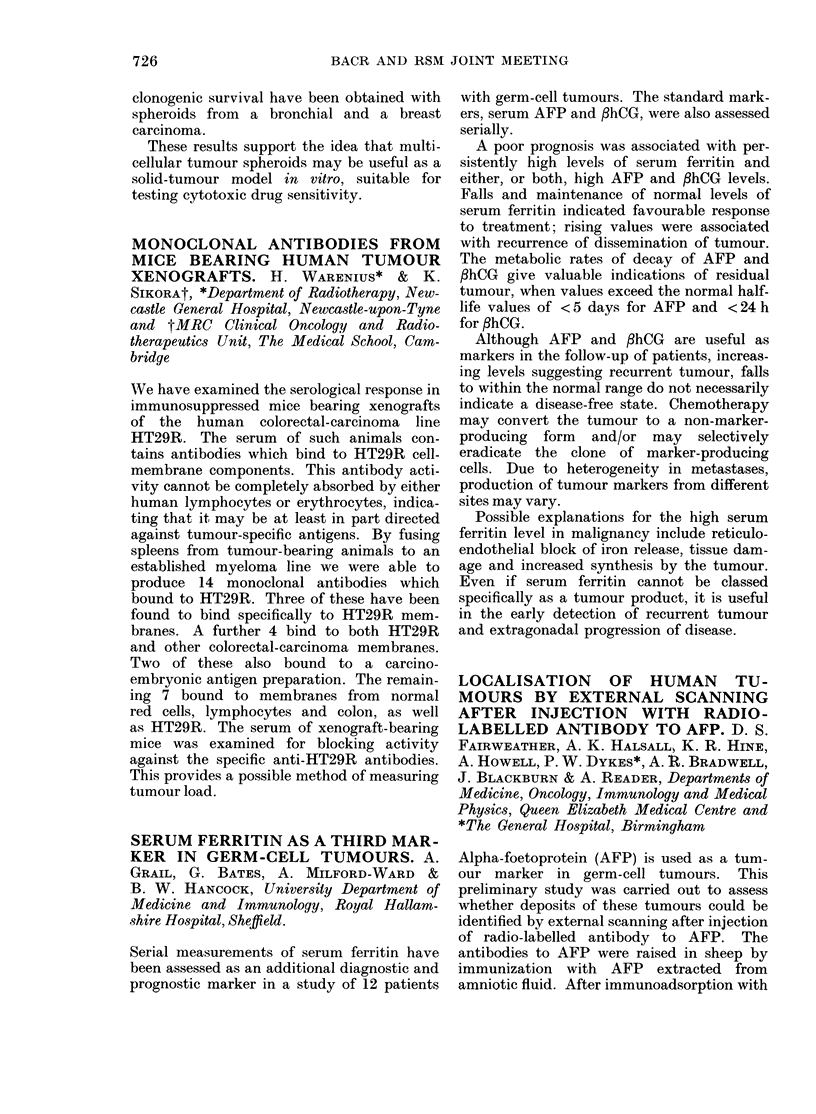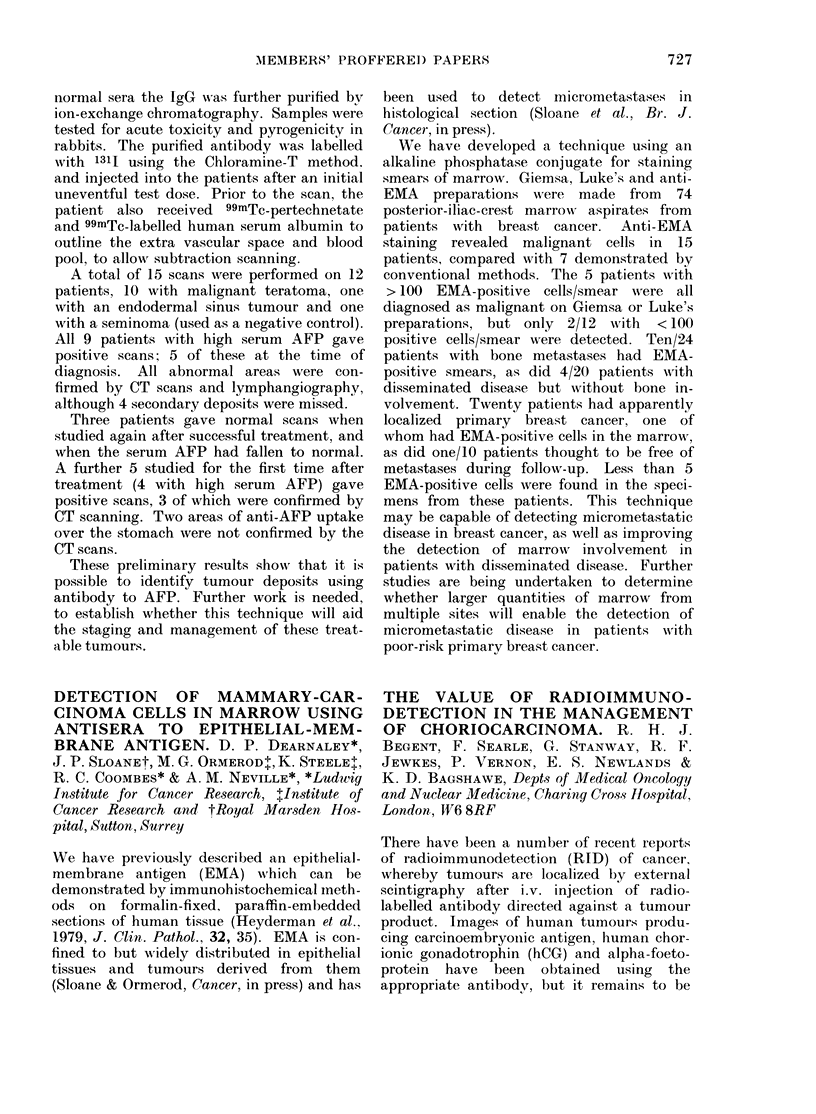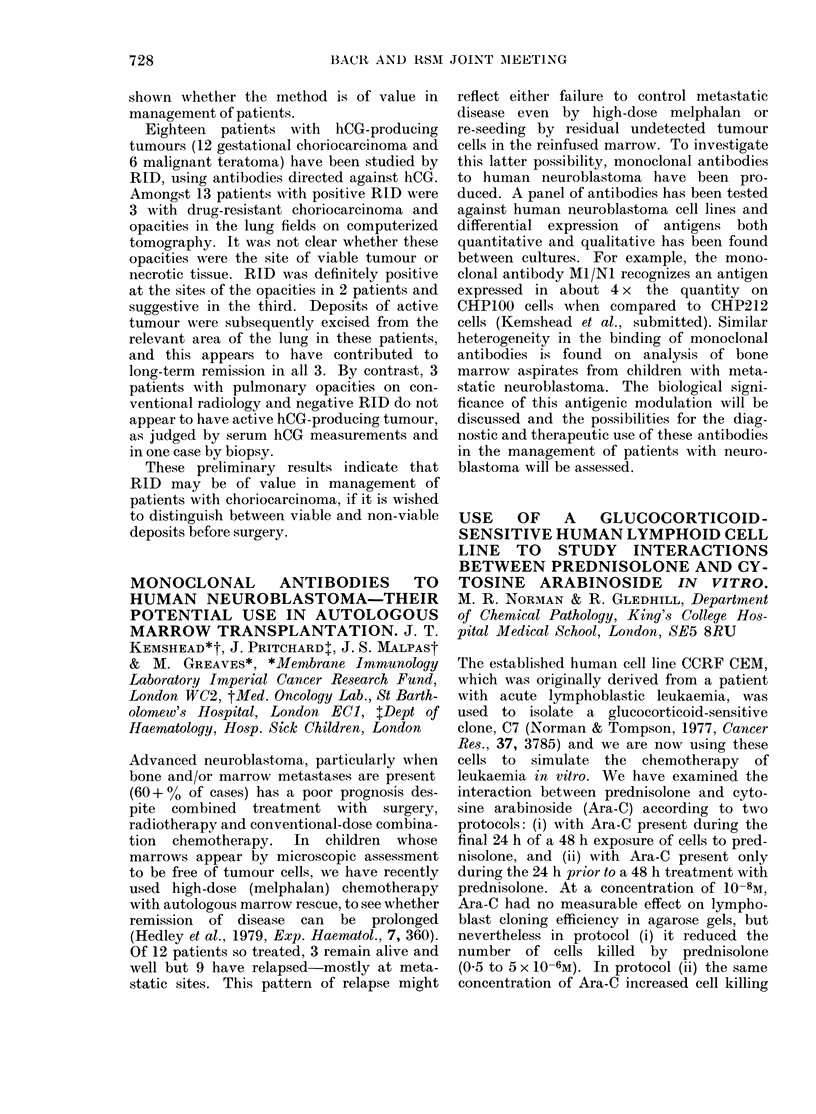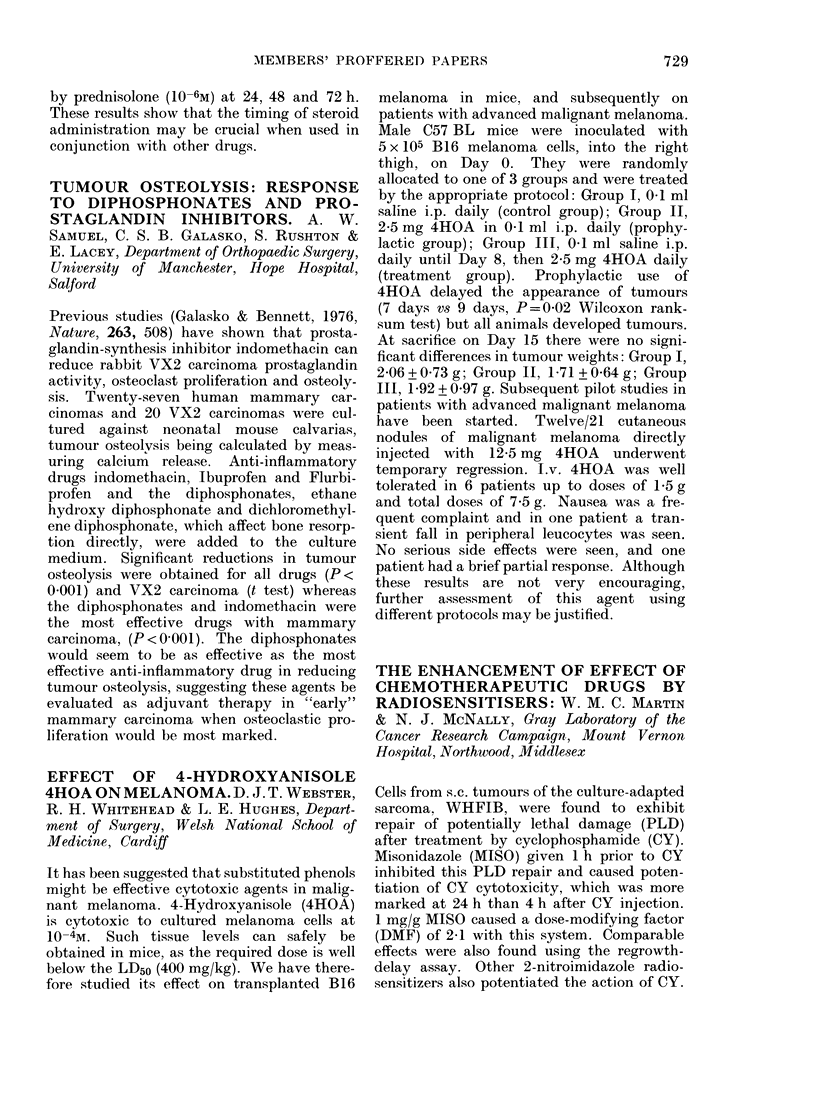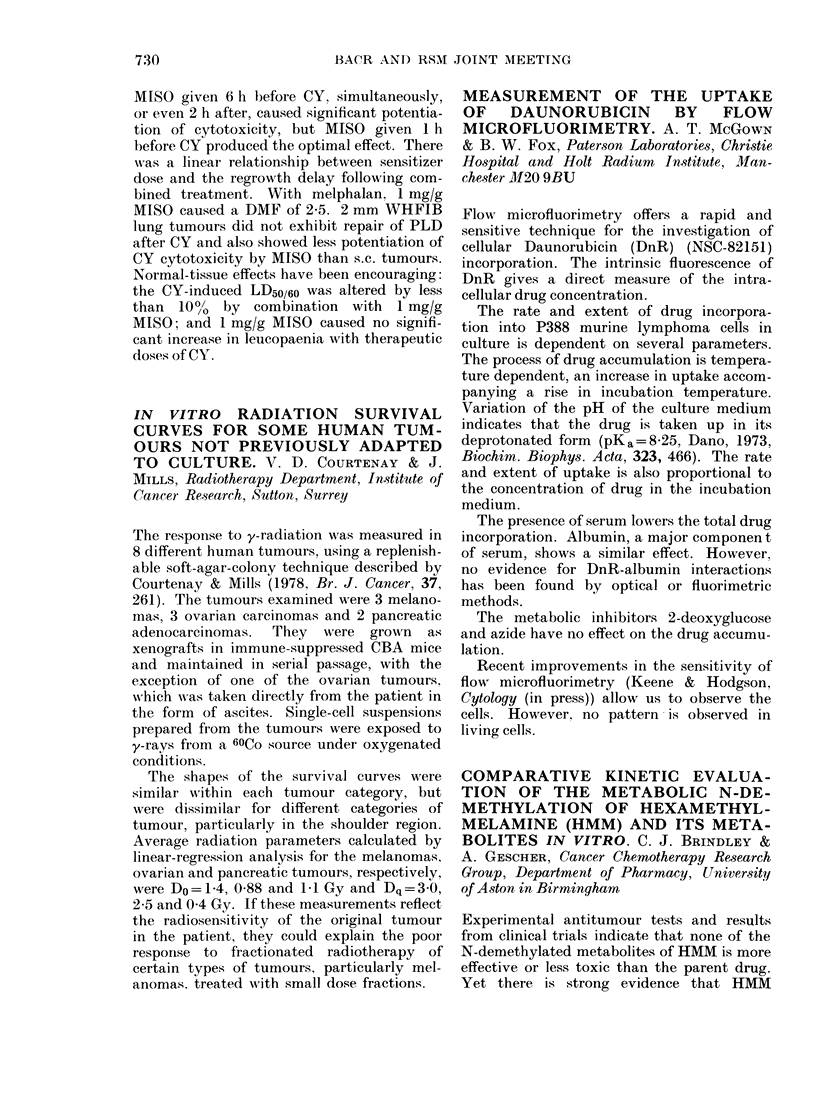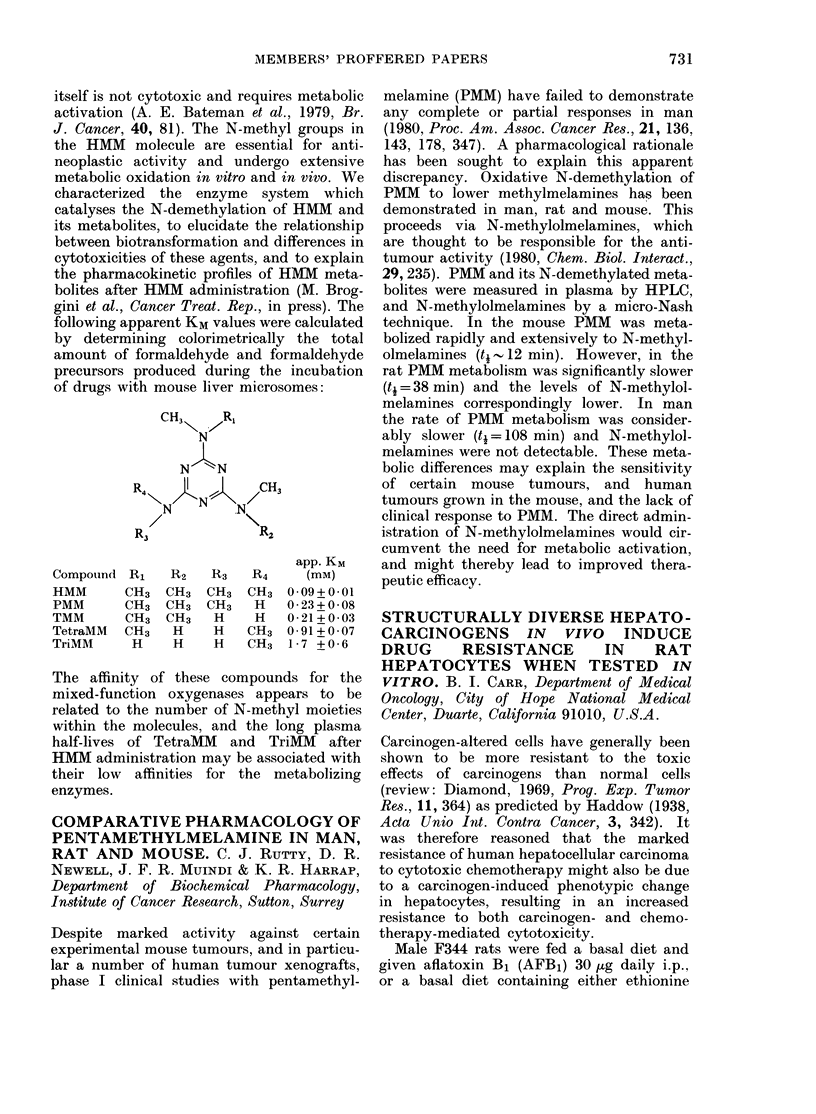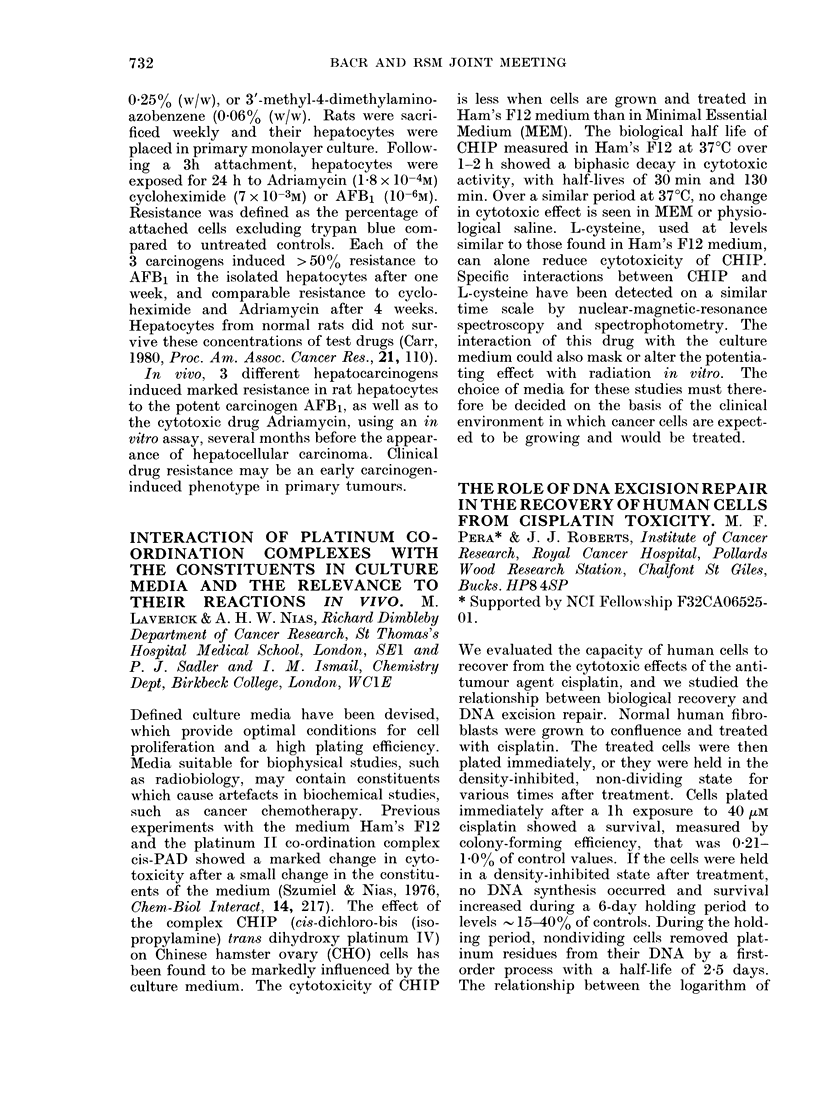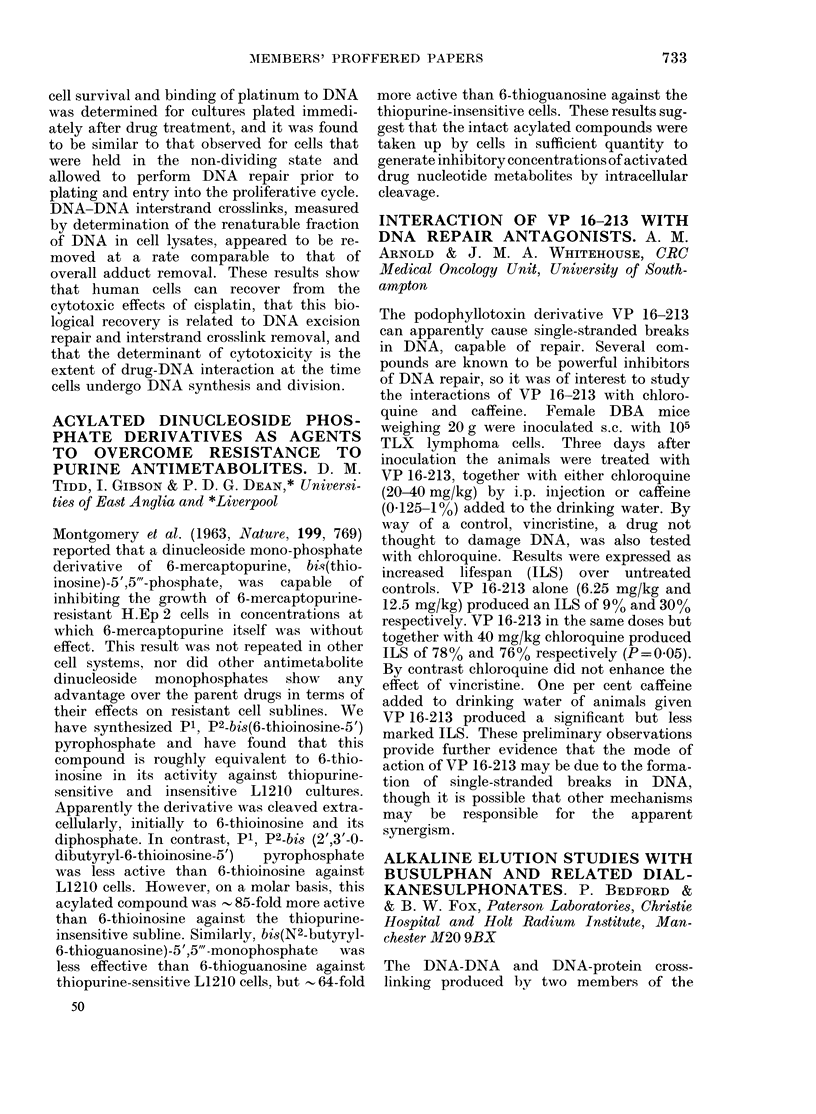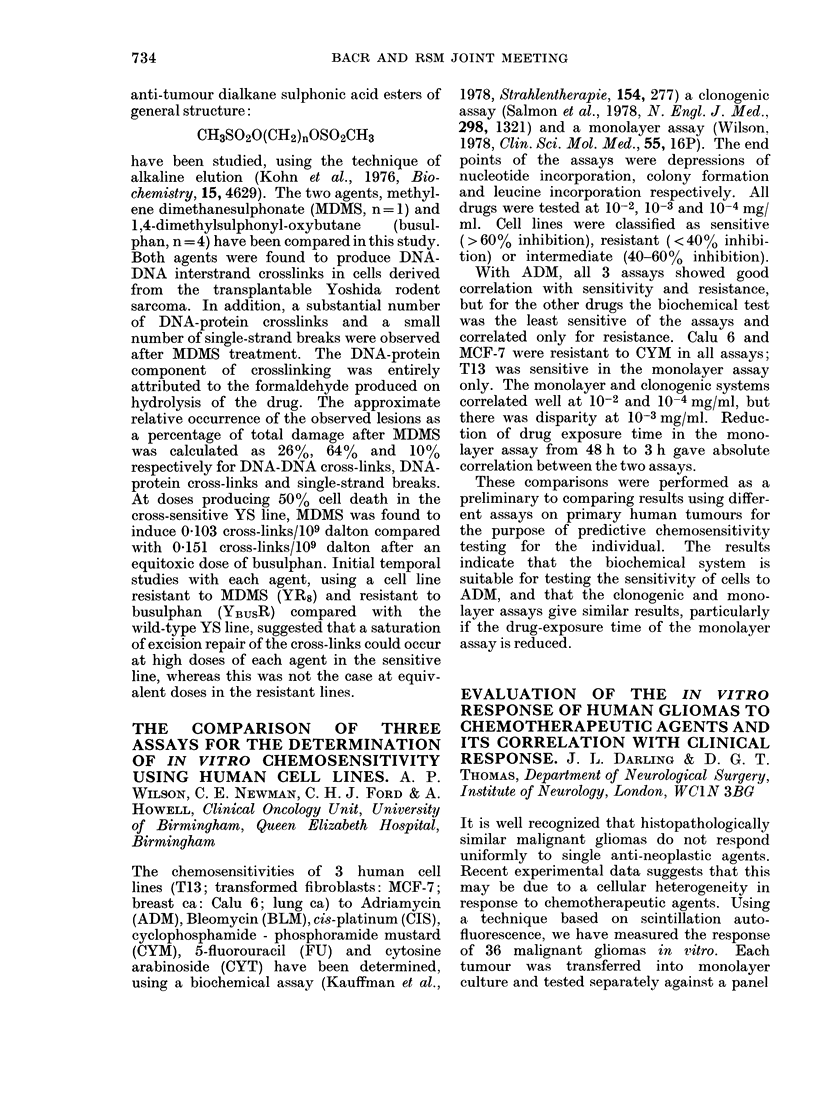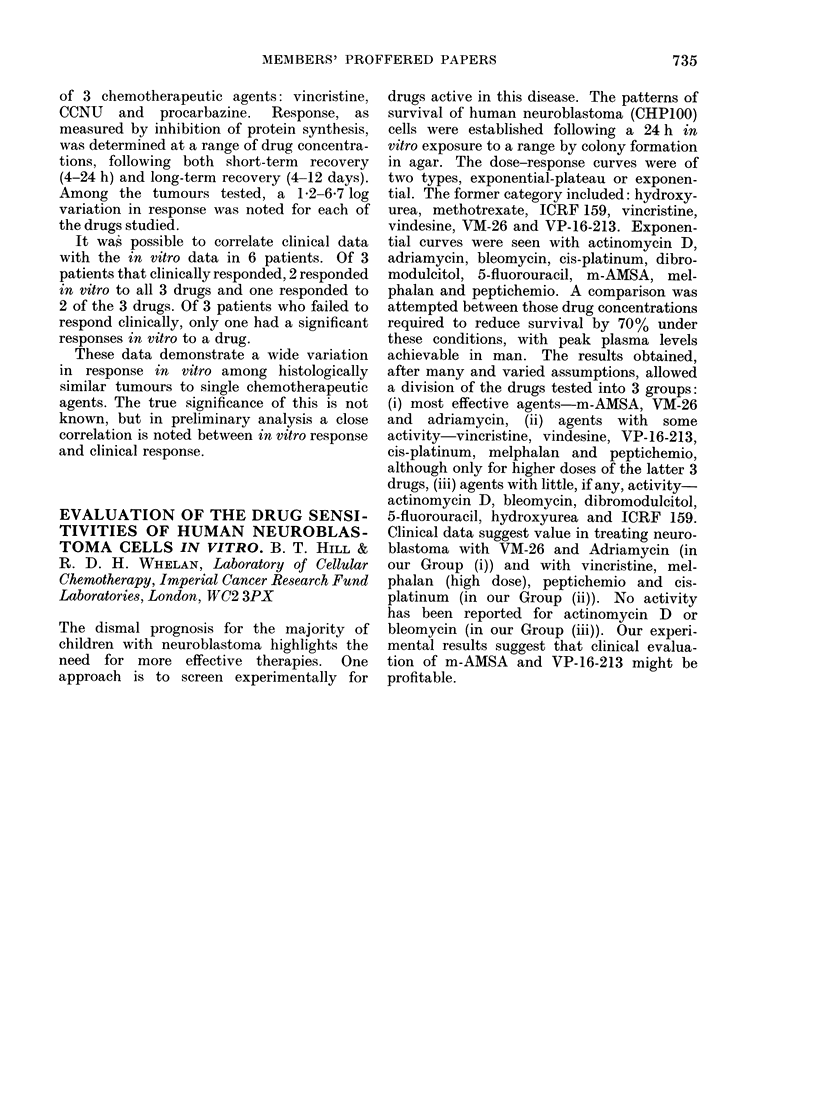# Transition from laboratory to clinic in cancer treatment. Abstracts of symposium papers.

**DOI:** 10.1038/bjc.1981.102

**Published:** 1981-05

**Authors:** 


*Br. J. Cancer* (1981) **43**, 701

### BRITISH ASSOCIATION FOR CANCER RESEARCH
### AND
### ROYAL SOCIETY OF MEDICINE (SECTION OF ONCOLOGY)

---

### JOINT MEETING

---

THE ROYAL SOCIETY OF MEDICINE, 1 WIMPOLE STREET, LONDON, W1

10 and 11 December 1980

### TRANSITION FROM LABORATORY TO CLINIC IN CANCER TREATMENT

### ABSTRACTS OF SYMPOSIUM PAPERS

### DESIGN AND EVALUATION OF SELECTIVE ANTICANCER DRUGS AND TREATMENT SCHEDULES

K. R. HARRAP

*From the Department of Biochemical Pharmacology, Institute of Cancer Research, Belmont, Sutton, Surrey, England*

MEMBERS OF the present generation of clinically used anticancer drugs are predominantly cytotoxic chemicals which exhibit little selectivity for tumour cells. There is an unequivocal need to discover and develop more selective compounds.

An essential requirement for selective drug design is the identification of a biochemical abnormality unique to the target tissue which might provide a focus for chemical attack. The most consistent qualitative abnormality reported in tumours is their aberrant regulation of gene expression, both transcriptional and translational processes being affected. These events are generally accompanied by only quantitative changes in intermediary metabolism; therapeutically exploitable enzyme defects appear to be rare, though not unique (Harrap, 1975, 1979). The altered transcriptional pattern of tumour cells appears to derive from structural and functional changes in the regulatory non-histone chromosomal proteins (Stein *et al.*, 1978). However, it is unquestionably premature to contemplate the design of agents which might selectively modulate these processes. Much work will first be required to isolate and characterize these proteins and to identify their regulatory functions. On the other hand, good progress has occurred in the design of small-molecule inhibitors capable of influencing the outcome of translational events. Notably it has been shown that S-adenosyl homocysteine is a strong competitive inhibitor of t-RNA methyltransferase activity, while some of its analogues exhibit selective avidities for individual enzymes (Trewyn & Kerr, 1976; Kogan *et al.*, 1976; Legraverend *et al.*, 1977).

Our own approach to the design of new agents has relied on identifying enzymes critical to the tumour, which are also amenable to selective chemical attack. In addition we have attempted to develop less toxic congeners of established drugs. We have also given some attention to the design of "host-protective" protocols incorporating drugs currently in clinical use. This programme will be elaborated under the following headings.

*Development of techniques designed to enhance the efficacy of established drugs*

In view of the poor performance of drugs currently in clinical use and the extensive delays involved in the introduction and acceptance of new agents into the clinic, there is a pressing need to improve the utility of the present generation of anticancer drugs. Accordingly we have devised techniques, based on the biochemical properties of individual drugs, which are protective in relation to host toxicity while also enhancing tumour-cell kill. Particular attention has been devoted to the alkylating agents and to methotrexate (MTX).

In the former case we have been able to show that the systemic toxicity of chlorambucil can be substantially reduced by the delayed administration of prednisolone, while the antitumour efficacy of the alkylating agent is considerably enhanced (Harrap *et al.*, 1977*a*). This has led to the successful replacement of mustine with chlorambucil in the MOPP schedule (McElwain *et al.*, 1977). Prednisolone appears to potentiate the cytotoxicity of several alkylating agents, this effect being associated with changes in nuclear protein structure and function (Riches & Harrap, 1973; Harrap *et al.*, 1975; Wilkinson & Harrap, 1973; Riches *et al.*, 1977). The improved therapeutic index of chlorambucil/prednisolone combinations appears to relate to steroid-enhanced binding of chlorambucil to tumour nuclear proteins, with concomitant suppression of binding to nuclear proteins from gut mucosal cells (Harrap *et al.*, 1980*c*; Wilkinson *et al.*, 1979).

The systemic toxicity incumbent on the use of MTX at high doses is routinely averted by the delayed administration of folinic acid. However, this technique also reduces the tumoricidal effects of the drug. We have found that it is possible to sustain the effects of the drug against tumour cells, while protecting susceptible host tissues from toxicity, by selective reversal of the drug's effects with combinations of purine and pyrimidine nucleosides (Harrap *et al.*, 1977*b*). The effects appear to depend on differences in purine/pyrimidine anabolism and catabolism in tumour and host tissues. It is also possible to reverse the systemic toxicity of MTX in man by a similar technique, though the ultimate clinical utility of the method awaits further evaluation (Dady *et al.*, 1980).

*Enzyme-targeted drugs*

In addition to the serendipitous discovery of qualitative enzyme defects in tumours, it is also possible to identify individual enzymes as potential targets for chemotherapy, based either on the importance of the enzyme to tumour proliferation *per se*, or on a knowledge of the molecular basis for treatment failure with a given agent. Examples will be quoted from three studies.

*Adenosine deaminase* (*ADA*).—Activity is extensively raised in T-cell acute leukaemias (Smyth & Harrap, 1975). The enzyme is apparently essential for lymphoid function, its genetic deletion being associated with the severe combined-immunodeficiency disease of childhood. The availability of deoxycoformycin, a tight-binding inhibitor of ADA, permitted us to explore the possible therapeutic utility of ADA inhibition. The antitumour efficacy of the inhibitor appears to derive from the generation of enhanced intracellular levels of dATP and associated inhibition of ribonucleotide reductase (Paine *et al.*, 1980*a*). In a Phase I clinical study the compound appeared to be selectively lymphotoxic, while some antitumour efficacy was apparent in T-cell acute leukaemias. However, attempts to potentiate the effects of deoxycoformycin in animals by concurrent administration of deoxyadenosine produced profound hepatotoxicity (Paine *et al.*, 1980*b*).

*Thymidine kinase*.—The ability of tumour cells to utilize (salvage) circulating pyrimidines may enable them to circumvent a drug-imposed blockade of *de novo* pyrimidine biosynthesis. The availability of inhibitors of thymidine kinase should permit the potentiation of antipyrimidines. We have been studying a series of mixed di- and triphosphonates of thymidine in relation to their affinity for the TTP (negative effector) site on the enzyme. One of these derivatives is 4 times as effective an inhibitor as TTP and prevents cells from utilizing thymidine at concentrations found in plasma.

*Thymidylate synthetase* (*TS*).—MTX is a tight-binding inhibitor of dihydrofolate reductase (DHFR) and a weak inhibitor of TS. Treatment failure is attributable either to impaired membrane transport or to the generation of high levels of DHFR. Under the latter circumstance, inhibition of TS by free intracellular MTX becomes qualitatively more significant: in highly MTX-resistant (enzyme) mutants the cytotoxic outcome of MTX treatment is determined entirely by inhibition of TS (which in any case is always the rate—limiting enzyme determinant of *de novo* thymidylate biosynthesis). A major component of the gastrointestinal toxicity induced by MTX may relate to its ability to induce rapidly a purineless condition in the gut (Straw *et al.*, 1977; Harrap *et al.*, 1977*b*) which cannot result if TS is selectively inhibited. For these reasons we have synthesized a range of quinazoline analogues of folic acid as potential inhibitors of TS. One of these, a 10-propargyl quinazoline, possesses a $K_i$ of $10^{-9}$ M for the enzyme. It exhibits substantially improved antitumour activity compared with MTX in a number of systems, and is also active against MTX-resistant cells (Jones, 1980; Calvert *et al.*, 1980; Harrap *et al.*, 1980*a*; Jones *et al.*, 1980).

*Synthesis of "Second Generation Drugs"*

It is possible to identify several clinically established drugs which possess useful antitumour properties, but whose intensive utilization is prohibited by the onset of severe dose-limiting toxic side effects. However, quantitative structure-activity studies permit the identification of congeners in which the useful antitumour properties of the parent compounds are preserved, with considerable reduction in systemic toxicity. In this connection our interest has been focused on the identification of second-generation alternatives to neoplatin (*cis*PtII), hexamethylmelamine (HMM) and DTIC (5-(3,3-dimethyltriazeno)imidazole-4-carboxamide).

Our evaluation of alternative platinum complexes has permitted the selection of cis-diammine-1,1-cyclobutane dicarboxylate-PtII as a viable candidate for early clinical trial. This compound is apparently not nephrotoxic, and is also less emetic than neoplatin. It also exhibits a wider spectrum of antitumour activity than the parent compound (Harrap *et al.*, 1980*b*). Studies with alternatives to HMM have led to the identification and clinical trial of a more water-soluble analogue, pentamethylmelamine, which can be administered systemically at well tolerated doses to provide circulating melamine levels about 20× those which can be obtained from a maximum tolerated dose of the parent compound (which must be given orally) (Smith *et al.*, 1980). Studies with DTIC analogues have shown that replacing the imidazole by an aromatic carrying structure yields a series of photostable derivatives possessing enhanced therapeutic indices against experimental tumours (Wilman & Goddard, 1980).

REFERENCES

CALVERT, A. H. *et al.* (1980). *Eur. J. Cancer*, **16**, 713.
DADY, P. J. *et al.* (1980) *Cancer Treat. Rep.*, **64** (in press).
HARRAP, K. R. (1975) In *Biology of Cancer*, Ellis Horwood, p. 96.
HARRAP, K. R. (1979) *Adv. Enzy. Regul.*, **17**, 457.
HARRAP, K. R. *et al.* (1975) *Excerpta Medica Int. Congr.* Series No. 375, 106.
HARRAP, K. R. *et al.* (1977*a*) *Eur. J. Cancer*, **13**, 873.
HARRAP, K. R. *et al.* (1977*b*) *Chem. Biol. Interact.* **18**, 119.
HARRAP, K. R. *et al.* (1980*a*) *Proc. Am. Ass. Cancer Res.*, **21**, 259.
HARRAP, K. R. *et al* (1980*b*) In *Cisplatin. Current Status and New Developments.* Academic Press. p. 193.
HARRAP, K. R. *et al.* (1980*c*) In *Molecular Actions and Targets for Cancer Chemotherapeutic Agents. Volume 2.* Academic Press (in press).
JONES, T. R. (1980) *Eur. J. Cancer*, **16**, 707
JONES, T. R. *et al.* (1980) *Eur. J. Cancer* (in press).
KOGAN, M. V. *et al.* (1976) *Mol. Biol.* (*USSR*), **10**, 73.
LEGRAVEREND, M. *et al.* (1977) *Eur. J. Med. Chem. Clin. Therap.* **12**, 105.
MCELWAIN, T. J. *et al.* (1977) *Br. J. Cancer*, **36**, 276.
PAINE, R. M., *et al.* (1980*a*) In *Purine Metabolism in Man* Volume 3. Plenum Publishing Corporation. p. 365.
PAINE, R. M. *et al.* (1980*b*) *Cancer Treat. Rep.* (in press).
RICHES, P. G. & HARRAP, K. R. (1973) *Cancer Res.*, **33**, 389.
RICHES, P. G. *et al.* (1977) *Chem. Biol. Interact.*, **18**, 11.
SMITH, I. E. *et al.*, (1980) *Proc. Am. Ass. Cancer Res.*, **21**, 136.
SMYTH, J. F. & HARRAP, K. R. (1975) *Br. J. Cancer*, **31**, 544.
STEIN, G. S. *et al.* (1978) *Cancer Res.* **38**, 1181.
STRAW, J. A. *et al.* (1977) *J. Natl Cancer Inst.*, **58**, 91.
TREWYN, R. W. & KERR, S. J. (1976) In *Oncodevelopmental Gene Expression.* New York: Academic Press. p. 101
WILKINSON, C. R. & HARRAP, K. R. (1977) *Br. J. Cancer*, **36**, 423.
WILKINSON, C. R. *et al.* (1979) *Cancer Res.*, **39**, 4256.
WILMAN, D. E. V. & GODDARD, P. M. (1980) *J. Med. Chem.* (in press).

### PRETREATMENT WITH CERTAIN CYTOTOXIC DRUGS REDUCES THE NORMAL TISSUE TOXICITY OF ANTI-CANCER AGENTS IN MICE AND MAN

J. L. MILLAR*, I. E. SMITH†, AND T. J. MCELWAIN†

* *From the Institute of Cancer Research and* †*Royal Marsden Hospital, Sutton, Surrey, England*

SEVERAL YEARS ago Smith and her colleagues noticed that pretreatment with certain stathmokinetic agents (namely vinblastine, vincristine or colchicine) two days before total-body irradiation reduced the marrow toxicity of the radiation in mice (Smith *et al.*, 1968; Brecher *et al.*, 1967). Independently Jeney and co-workers, using rats, established that pretreatment with low-dose merophan or melphalan protected animals from an otherwise lethal dose of merophan or melphalan respectively (Jeney *et al.*, 1968). Neither of these two research groups examined the effect of these normal tissue-sparing combinations on malignant tissue.

More recently these studies have been extended to show that a wide variety of cytotoxic agents, when used at appropriate low doses will protect against radiation or drug-induced marrow lethality. These agents include cyclophosphamide (cy), cytosine arabinoside, methotrexate and chlorambucil Millar *et al.*, 1975, 1978*a*.

The marrow is not the only mouse normal tissue that can be spared by drug pretreatment. Studies involving the administration of cy showed that pretreatment with low-dose cy 4 days before high-dose cy reduced the damage caused by the high dose of cy to the urothelium and improved animal survival Millar & McElwain, 1978). Further, toxicity to intestinal epithelium caused by high-dose melphalan can be reduced by pretreatment 2 days before with low-dose cy, cytosine arabinoside or melphalan itself (Millar *et al.*, 1978*b*). Radiation given at high enough doses also critically damages this tissue, and it has been shown that cytosine arabinoside given 12 h before irradiation reduces the damage to the gut (Phelps & Blackett, 1978). It is likely that cell synchrony plays a part in the protection phenomenon in this instance.

Although the investigations of these normal-tissue-sparing combinations on tumours have not been extensive, the results so far have suggested that tumour tissue may not be similarly spared. Using the normal-tissue-sparing combination of 50 mg/kg cy 4 days before high-dose cy (250–350 mg/kg) in a curability experiment involving the Lewis lung carcinoma, it was established that this sequency of drug administration maintained its expected anti-tumour efficacy (Millar & McElwain, 1978). Further, in studies involving drug-induced growth delay of the FS6 fibrosarcoma, it was established that the pretreatment dose could be escalated without reversing the normal-tissue-sparing properties of the sequence, yet improving the anti-tumour effect (Millar *et al.*, 1980).

In experiments involving the marrow-sparing combination of cytosine arabinoside 2 days before irradiation, growth-delay studies with the Lewis lung tumour indicated that pretreatment with cytosine arabinoside neither protected tumour cells nor enhanced regrowth of tumour cells that survived the irradiation (T. Stephens and J. Peacock, personal communication) again suggesting that malignant tissue does not respond in the favourable way in which marrow or intestinal epithelium respond to this combination.

Although several hypotheses have been put forward to explain this selective protection of normal tissue, they have all been disproved and the phenomenon still awaits convincing explanation.

In the meantime, steps have been taken to investigate the phenomenon in larger mammals and, as a first step, sheep were used and the effect of high-dose melphalan on sheep intestinal epithelium with and without cy pretreatment was investigated. It was established that pretreatment with cy reduced the toxicity of melphalan to the intestinal epithelium when given 5–9 days before the dose of melphalan (Millar *et al.*, 1978*c*). This indicated that the time interval between pretreatment and challenge was longer for large mammals, and an interval of 7 days was used in studying human tissue response.

Human tumours have been grown in immune-deprived mice for some time in this laboratory (Steel *et al.*, 1978). Human melanoma xenografts were used in a growth-delay study to establish whether a normal-tissue-sparing combination of cy and melphalan reduced the efficacy of the drugs against melanoma. No such alteration in efficacy was seen, suggesting that, once again, combinations of drugs which spare normal tissue do not spare tumour tissue.

The study of the combination of cy 7 days before high-dose melphalan in the treatment of disseminated malignant melanoma in man was instigated. Previously, doses of melphalan ranging from 60 to 120 mg/m$^2$ had been used in single-agent studies, and a pattern of marrow suppression reflected in leukopenia had been established. A pretreatment dose of 300 mg/m$^2$ cy was given 7 days before 140 mg/m$^2$ melphalan and it was noticed that the peripheral leucocytes recovered slightly more rapidly in these patients than in the group that were not pretreated (Hedley *et al.*, 1978). The clinical response of the tumour appeared to be unaltered by the cy pretreatment.

In conclusion, there are combinations of cytotoxic agents which spare normal tissue such as marrow, intestinal epithelium and urothelium. This phenomenon has been observed in mice, rats, sheep and man. In all experimental tumour systems tested so far these normal-tissue-sparing combinations appear to maintain their anti-tumour efficacy, thus improving the therapeutic index.

REFERENCES

Brecher, G. *et al* (1967) *Radiat. Res.*, **30**, 600.
Hedley, D. W. *et al.* (1978) *Lancet*, iv, 996.
Jeney, A. *et al.* (1968) *Acta Physiol. Acad. Sci. Hung.*, **33**, 89.
Millar, J. L. *et al.* (1975) *Br. J. Cancer*, **32**, 193.
Millar, J. L. & McElwain, T. J. (1978) *Antibiot. Chemother.*, **23**, 271.
Millar, J. L. *et al.* (1978*a*) *Cell Tissue Kinet.*, **11**, 543.
Millar, J. L. *et al.* (1978*b*) *Br. J. Cancer*, **38**, 137.
Millar, J. L. *et al.* (1978*c*) *Eur. J. Cancer*, **14**, 1283.
Millar, J. L. *et al.* (1980) *Br. J. Cancer*, **42**, 485.
Phelps, T. A. & Blackett, N. M. (1978) *Int. J. Radiat. Oncol. Biol. Phys.*, **5**, 1617.
Smith, W. W. *et al.* (1968) *J. Natl Cancer Inst.* **40**, 847.
Steel, G. G. *et al.* (1978) *Br. J. Cancer*, **37**, 224.

### THERAPY OF GASTROINTESTINAL CANCER

J. S. MacDONALD

*From the National Cancer Institute, U.S.A.*

The treatment of adenocarcinoma of the stomach, pancreas and colon has generally been surgical in the past. However, recent clinical trials have suggested that chemotherapy and combined chemotherapeutic and radiation therapy programmes may be making major gains in both the adjuvant treatment of these diseases and in the treatment of advanced unresectable cancers. In the management of locally unresected gastric cancers, a study recently reported by the Gastrointestinal Tumour Study Group (Schien & Novak, 1980) has shown that the combination of 50 Gy of split-course irradiation followed by 5 FU plus Methyl CCNU combination chemotherapy is superior to 5-FU plus MeCCNU alone. In this two-armed randomized trial in which 45 patients were treated with chemotherapy and 45 with chemotherapy plus radiation, it was shown that although the median survival was better for the chemotherapy alone patients, (18 months *vs* 9 months) the long-term survival was better for patients treated with the combined modality. Eight of 45 patients (18%) in the combined-modality arm were alive and free of disease at more than $3\frac{1}{2}$ years after diagnosis. In the chemotherapy-alone arm only 3/45 patients (7%) survived. The nearly 20% survival of patients with documented unresectable gastric cancer suggests that combined-modality therapy with local radiation followed by systemic chemotherapy should be used as a standard arm to which new therapies should be compared in future studies of locally unresectable gastric cancer.

In patients with advanced gastric cancer, major advances have been made through the use of combination chemotherapy (Macdonald *et al.*, 1979). The FAM (5-fluorouracil, Adriamycin and mitomycin-C) chemotherapy regimen, designed by investigators at the Vincent T. Lombardi Cancer Research Center at Georgetown University, has shown particularly promising results in advanced gastric-cancer patients. In a series of 62 patients with advanced measurable malignancy, 26 partial responses were noted. This 42% response has been confirmed in studies from the University of Chicago (Bitran *et al.*, 1979). and Southwest Oncology Group (Panettiere & Heilbrun, 1976). The median duration of response in the Georgetown study was 9 months, and median survival in responding patients was 12 months. These responses and survival durations suggest that the FAM regimen should be tested in prospective trials as an adjuvant to surgery in gastric cancer. Such studies are now under way.

The medical management of pancreatic cancer has been generally unsatisfactory, though there is now evidence that the combined use of radiation therapy and 5-FU may be superior to radiation alone in locally unresectable patients (Lokich, 1979). A study performed by the Gastrointestinal Tumour Study Group in which patients with unresectable pancreatic cancer were randomly allocated to receive either 60 Gy of split-course radiation or 60 Gy plus 5-FU or 40 Gy of split course radiation plus 5-FU showed that the combined modality arms of radiation plus 5-FU were statistically superior in prolonging survival to radiation therapy alone. The median survival in the combined-modality arms varied between 36 and 41 weeks whereas the median survival after 60 Gy radiation alone was 21 weeks. The median survival in patients receiving no therapy after documentation of unresectable pancreatic cancer is about 12–16 weeks.

In patients with colorectal cancer, there has been much interest in the use of adjuvant chemotherapy or combined-modality radiation and chemotherapy. In the United States over 100,000 new cases of colorectal cancer occur per year, and the 5-year survival with surgery alone is $\sim$50%. In patients with resected Dukes B or C colon cancer there have been numerous adjuvant chemotherapy studies (Moertel, 1976). These studies have used single agents, combinations of agents and occasionally combinations of chemotherapy and immunotherapy. Appropriately designed studies of adjuvant therapy in colon cancer must at present incorporate a control arm treated with surgery alone, since there has been no confirmation of the effectiveness of any adjuvant therapy. The

Gastrointestinal Tumour Study Group (unpublished) has recently completed a 4-arm controlled study in which surgery alone is compared to 5-FU plus MeCCNU chemotherapy, MER (methanol-extracted residue of BCG) immunotherapy and chemoimmunotherapy with 5-FU MeCCNU and MER. The study has over 550 patients, and at present there appears to be no difference between any of the treatment arms and the surgery alone arm. It is important to note, however, that in carefully staged and operated patients, as demonstrated in this study, the survival of patients with Dukes B2 disease is excellent and is $>75\%$ at 2 years after surgery.

Because of the prevalence of local recurrence in patients with rectal cancer, studies have concentrated on the use of radiation therapy or radiation therapy plus chemotherapy as an adjuvant to surgery in these patients. Although there have been suggestions that both low dose (5–25 Gy) and high-dose ($>40$ Gy) preoperative therapy are useful in preventing recurrence of rectal cancer and prolonging survival, it is difficult to find carefully controlled prospectively randomized studies in which clear benefit is statistically confirmed. Recently the Gastrointestinal Tumour Study Group (unpublished) has analysed initial results of a randomized controlled study which compared: (1) high-dose postoperative radiation therapy, (2) 5-FU plus MeCCNU, and (3) the combination of postoperative radiation therapy and 5-FU plus MeCCNU. A 4th arm in this study was surgery alone. With 187 patients enrolled in this study, clear benefit from adjunctive therapy has been demonstrated. The relapse rate in the patients treated with surgery alone was 52% *vs* 21% in patients treated with chemotherapy plus radiation, 39% in patients treated with chemotherapy alone and 32% in patients treated with radiation alone. The median follow-up of all patients in the study was 118 weeks. The 75% disease-free survival in weeks was 50 for the control arm, 78 for the chemotherapy arm, 71 for the radiation arm and the 75% survival had not been reached in the chemotherapy plus radiation arm. There was also a striking decrease in local recurrence in the patients receiving chemotherapy plus radiation. In this arm, only 3% of patients demonstrated local recurrence, compared to 23% in the chemotherapy arm and 15% in the radiation arm. This study is continuing to accrue patients to the 3 treatment arms to obtain information on which arm is superior in overall survival and disease-free survival. The importance of this study is that it is a prospectively controlled trial which has clearly shown that adjuvant treatment after rectal resection is superior to surgery alone. Thus, this approach will have to be considered the treatment with which future experimental treatments of rectal cancer will be compared.

The active investigation of non-surgical management in both advanced, locally advanced and surgically resected gastrointestinal cancers is beginning to show substantial benefits both in palliation of patients' symptoms and improvement in survival. It is clear that continued active investigation will result in even more striking benefits to the patients afflicted with these diseases.

### REFERENCES

Bitran, J. D. *et al.* (1979) *Cancer Treat. Rep.*, **63**, 2049.

Lokich, J. (1979) *Int. J. Radiat. Oncol.*, **5**, 1643.

Macdonald, J. S. *et al* (1979) *Cancer*, **44**, 42.

Moertel, C. G. (1976) *Clin. Gastroenterol.*, **5**, 777.

Panettiere, F. J. & Heilbrun, L. (1976) In *Mitomycin-C.* Academic Press. p. 145.

Schein, P. S. & Novak, J. (1980) *Proc. ASCO and AACR*, **21**, 419.

### SELECTION, EVALUATION, AND DEVELOPMENT OF ANTITUMOR DRUGS AND THEIR RELEVANCE TO THE CLINIC

A. GOLDIN

*From the Division of Cancer Treatment, National Cancer Institute, U.S.A.*

The primary objective of preclinical antitumour drug-testing programmes is to select, evaluate and develop materials from the universe of synthetic and natural substances, that will have the greatest likelihood of usefulness in the treatment of clinical neoplasia. Historically, there has been a reasonable measure of success in the identification and introduction of useful antitumour agents to the clinic, even though the experimental systems used in the screening were chosen and utilized primarily on an empirical basis. Cancers for which drugs have been responsible for some of the patients achieving a normal life span include acute lymphocytic leukaemia (pediatric) acute myelogenous leukaemia (adult) Hodgkin's disease, diffuse histiocytic lymphoma, nodular mixed lymphomas, Burkitt's lymphoma, Ewing's sarcoma, rhabdomyosarcoma, Wilms' tumour, choriocarcinoma, testicular cancer and ovarian cancer. However, these responsive tumours constitute only a small fraction of the various types of cancer, and there is a need for new and highly effective drugs for the treatment of clinical solid tumours. Over the years, there has been the important concern at the Division of Cancer Treatment (DCT), National Cancer Institute (NCI) screening and drug development programme that the test models relying heavily on leukaemias L1210 and P388, with Lewis lung carcinoma and B16 melanoma as additional systems, might not be identifying compounds that would be most active against human solid tumours. With the demonstration that human tumours could be transplanted and grown progressively in athymic mice with retention of original biological characteristics, and with the development of new animal models, and based on broad experience, both at the NCI and abroad, re-examination of the procedures and concepts of antitumour drug selection, evaluation and development seemed in order.

As a result, in 1976, the DCT NCI, decided to reorganize its entire screening programme with the incorporation of a prescreen (leukaemia P388), and a bypass system for candidate agents of interest because of activity in either other screening programmes or in selected biochemical or biological assays. P388 actives and the bypass compounds are tested in a panel of tumours, including well established conventional experimental tumour systems, more recently developed animal solid-tumour models and human tumours growing as xenografts in athymic mice. The new programme is considered as a comprehensive and co-ordinated prospective screening experiment for which specific questions are addressed to the screen as follows:

(1) Does the new screen increase the yield of true positive compounds (active in both screen and clinic)?

(2) Does extensive and/or broad spectrum activity in the screening panel increase the probability of clinical antitumour effectiveness?

(3) Do human tumour xenografts and animal tumour screens select the same or different drugs?

(4) Are the xenograft positives more active in the clinic than those selected by animal screens?

(5) Does the screen reduce the number of false positives (active in the screen but not active in the clinic)?

(6) Does it reduce the number of false negatives (inactive in the screen, but active in the clinic)?

(7) Is there a correspondence of activity against animal tumours and/or human tumour xenografts with activity against clinical tumours for specific histological types or specific organs?

(8) Are compounds that bypass the P388 prescreen, because of special characteristics, more effective in the screening panel and in the clinic than compounds initially selected by the prescreen?

(9) What contribution will the new screening panel make to prediction of clinical effectiveness of new drugs with respect to structure-activity analysis, analogues of known antitumour agents, and mathematical approaches to activity prediction?

The new screening programme will be described, data presented on compounds emerging from the screen, and the answers to the questions addressed to the new screen will be reviewed.

Once a compound has been determined as having significant activity against one or more of the target models, it is subjected to further evaluation and development in proceeding to the clinic. Various systems that may be used for further evaluation of antitumour agents, and preclinical principles for improvement of selectivity, will be discussed and examples cited. These take into account the complexity of interrelationships of the triad of host, tumour and drug.

The programme of drug development, involving decision making in a linear array of steps, will be described.

The primary theme is that a screening, evaluation and development programme should be considered as a dynamic entity subject to investigation and change in the search for new and more effective antitumour agents.

### THIRD ALEXANDER HADDOW MEMORIAL LECTURE

### Magic Bullet or Tragic Bungle: Problems in Transition of Anticancer Drugs from Laboratory to Clinic

Professor K. HELLMANN

*Imperial Cancer Research Fund, Lincoln's Inn Fields, London*

### HYPOXIC CELL SENSITIZERS IN RADIATION THERAPY

G. E. ADAMS AND I. J. STRATFORD

*From the Division of Physics, Institute of Cancer Research, Sutton, Surrey, England*

DESPITE THE many advances in the treatment of cancer by radiotherapy, failure to achieve local control is still a substantial problem in some disease sites. Even though metastatic disease remains a major problem in cancer therapy, improvements in local control rates should be reflected in a significant reduction in overall cancer mortality.

Although there are various reasons for local failure in radiotherapy, one of the most likely is the problem of hypoxic-cell radiation resistance.

Hypoxic cells, which are probably present in most solid tumours, arise as a result of tumour growth outstripping the blood supply, and occur usually in and around apparently necrotic regions of tumours. Hypoxic cells are believed to be dangerous because of their radiation resistance. Such cells are in a resting state and normally would eventually die of starvation of $O_2$ and other essential nutrients due to their remoteness from the blood supply. However, subsequent to, or during, fractionated radiation treatment, tumour regression can occur following removal of oxic cells sterilized by the radiation. This allows some of the radiation-resistant hypoxic cells to re-oxygenate, enter cycle and permit regrowth of the tumour. There is now definite clinical evidence that hypoxia can contribute to overall radiation resistance in some human tumours, and during the last two decades several approaches have been made to overcome this hypoxia problem. These include unconventional fractionation regimes (to exploit re-oxygenation) radiotherapy in hyperbaric $O_2$ chambers, neutron radiotherapy and chemical radiation sensitizers. The major steps in the transition from the laboratory to the clinic in the field of chemical sensitizers will be outlined in the lecture. This will illustrate the continuous and systematic development of research originating in basic radiation chemistry, progressing through cellular and tumour radiobiology, pharmacology and toxicology, to the current situation where widespread clinical trials of hypoxic cell sensitization are now in progress.

The generality of the $O_2$ effect in radiobiology encouraged the search for other chemical agents that might act by a similar mechanism. As a result, many compounds are now known which are able to act as "oxygen-mimics" in radiation sensitization. These include simple aliphatic compounds containing conjugate electron-acceptor groups, quinones, various aromatic structures and, in particular, nitroimidazoles. Some of these agents, which like $O_2$ act by increasing the radiation sensitivity of hypoxic cells, are in contrast metabolically stable and can penetrate into the hypoxic regions of tumours in order to sensitize cells at the time of irradiation.

Radiation sensitization *in vitro* by nitroimidazoles is dependent upon their electron affinity, and most of these compounds only act by increasing the radiosensitivity of hypoxic cells (Adams *et al.*, 1979); well-oxygenated cells are unaffected. This differential property remains the main rationale behind the use of radiation sensitizers in the clinic. The radiation response of well-oxygenated normal tissue should not therefore be affected. Among the group of sensitizers of most current interest is misonidazole (MISO). Hypoxic cells irradiated in the presence of a sufficient concentration of this drug can be rendered almost as radiation-sensitive as well-oxygenated cells. Many studies have now been carried out with a variety of experimental tumours, including human tumours growing as xenografts in immune-suppressed mice. A review of such data (Fowler & Denekamp, 1979) shows that marked radiation sensitization occurs in almost all tumours, indicating that hypoxic-cell radiation resistance is a common property of most experimental solid tumours.

It is clear, however, that the extent of hypoxia can be reduced by re-oxygenation when the tumours are irradiated with multiple fractions of radiation. This is reflected by the reduction of radiation sensitization by MISO when the drug is administered with such radiation regimens. Nevertheless, radiation sensitization *still* occurs in most cases demonstrating that re-oxygenation processes, though reducing the hypoxic cell population, does not eliminate the problem. This suggests that the use of sensitizers in the clinic may take some of the criticality out of the future choice of fractionation regimens.

Pharmacokinetic studies with MISO in rodents, dog and man, have shown that the drug is widely distributed in the body. Further, it has a fairly long half-life (mouse: 1–2 h, dog: ~5 h man 10–12 h) and in both rodent and human tumours is able to penetrate with generally high efficiency into the regions where hypoxic cells occur (Ash *et al.*, 1979).

Although MISO is well tolerated in fairly high doses in man, it is neurotoxic. Many Phase I and II clinical studies have shown that a significant proportion of patients develop a sensory peripheral neuropathy, usually shortly after the end of treatments with multiple doses. Most current studies have indicated that the maximum dose for the total fractionated treatment should not exceed 12 g/m$^2$. Since the drug dose given with each radiation fraction will clearly depend on the total number of fractions given, choice of fractionation is important in clinical trials with MISO. This has obviously influenced the design of the numerous clinical trials with MISO now in progress in various parts of the world. These studies, which include both conventional and unconventional regimes for radiation fractionation, will be discussed.

It is now clear, however, that irrespective of the regimen chosen, the tolerated doses of MISO are such that tumour levels of the drug, although fairly high, are not sufficient to give the theoretical maximum radiation sensitization. This has encouraged the search for new drugs which are either more efficient or less toxic. This search has been aided considerably by the predictive value of electron affinity. A number of promising new compounds have already been identified which are more efficient radiation sensitizers than MISO (Adams *et al.*, 1980).

In parallel with these developments, much research has been carried out aimed at identifying the reasons for the neurotoxic properties of these compounds. Results from some of the experimental and clinical studies will be described.

Another property of MISO and other nitroimidazoles is their increased cytotoxicity to hypoxic than to aerobic cells. The mechanism of the cytotoxic action is not related to the radiosensitization and appears to involve the anaerobic reduction of the nitro group in the compound.

It is possible that the presence of hypoxic cells in tumours may influence the outcome of cytotoxic chemotherapy. If so, nitroimidazoles may have a part to play in the combination chemotherapy of cancer. It has been found in a number of experimental tumour systems that MISO administered either simultaneously with, or just before, treatment of tumour-bearing mice with an alkylating agent, substantially increases the anti-tumour effect of the second drug (Wodinsky *et al.*, 1979; Rose *et al.*, 1980. This has been found in a variety of tumours using cyclophosphamide, melphalan or nitrosoureas in combination with MISO, or related nitroimidazoles. Possible mechanisms of this interesting and potentially useful effect will be discussed.

REFERENCES

ADAMS, G. E. *et al.* (1979) *Int. J. Radiat. Biol.*, **35**, 133.
ADAMS, G. E. *et al.* (1980) *Cancer Clin. Trials*, **3**, 37.
ASH, D. V. *et al.* (1979) *Br. J. Cancer*, **39**, 503.
FOWLER, J. F. & DENEKAMP, J. (1979) *Pharmacol. Ther.*, **7**, 413.
ROSE, C. M. *et al.* (1980) *Cancer Clin. Trials*, **3** (in press).
WODINSKY, I. *et al.* (1979) *Proc. Am. Assoc. Cancer Res.*, **70**, 230.

### HYPERTHERMIA: MOUSE, MICROWAVES AND MAN

S. B. FIELD

*From the MRC Cyclotron Unit, Hammersmith Hospital, London W12 0HS*

THE DISAPPEARANCE of tumour after high fever was reported more than a century ago Resulting from this observation, hyperthermia was induced by injection of bacterial toxins. Methods for local heating were sought but these techniques are difficult, and interest in the possibility of hyperthermic treatment faded. However, a firm rationale has now been developed for using hyperthermia, and this in turn has stimulated further technical developments, so that cancer therapy by hyperthermia has become of great interest.

Reasons why hyperthermia might be useful in treating cancer:

(1) Tumours may become hotter than normal tissues in a localized treatment field because of their more sluggish blood supply;

(2) Some neoplastic cells will be intrinsically more sensitive than the normal cells at risk;

(3) Tumours contain cells which are:
- (a) hypoxic, which seems not to markedly affect their response to hyperthermia (in contrast to X-rays) and
- (b) at low pH and nutrient-deficient, both making cells more sensitive to heat.

(4) Cells in the S phase are particularly sensitive to heat but relatively resistant to X-rays, so that a combined treatment with hyperthermia and radio-therapy might be an advantage in some circumstances.

(5) Combining heat with drugs may enhance the therapeutic effect either by increasing the drug uptake or enhancing the cells sensitivity to the drug.

The evidence for these statements is derived from much experimental work on cells *in vitro* and both normal and malignant tissues *in situ*.

The effects of heat alone are different from those of heat as a potentiator of other anticancer modalities, and it is therefore important to find ways of studying the effects separately. This may be done without difficulty on several normal tissues, making use of qualitative and temporal differences in response to the different modalities. This is possible because after radiation most cells die at mitosis, whereas after heat, nondividing cells lyse just as readily as mitotic cells. Skin, for example, shows radiation damage as radiodermititis. The injury is enhanced by moderate hyperthermia but is qualitatively unaltered. In contrast, severe hyperthermia rapidly causes tissue necrosis. Similar separations have been made on intestine and cartilage. Such separations are far more difficult with cells *in vitro* or with tumour responses, because in both these cases the endpoints are normally measured as a given number of surviving cells, regardless of the mode of cell death.

With heat alone, once the threshold for injury is reached, a small increase in hyperthermal treatment (temperature or time) will cause a dramatic increase in the probability of tissue necrosis. Because of this, very careful control of heat delivery and accurate thermometry are required in the clinic. For heat alone it is found with a wide range of cells and tissues that a change in temperature of 1°C is equivalent to a 2-fold change in heating time.

In clinical practice it is likely that treatment will be given in many fractions, as in radiotherapy. It is therefore important to gain an understanding of fractionation in hyperthermia. It has been shown, with cells *in vitro* and in animals, that there is a considerable recovery potential between heat doses but, in addition, heat causes a transient resistance to subsequent hyperthermia (termed thermotolerance). This is an extremely large effect, such that a second treatment may have to be more than doubled to produce a specified effect if given after a previous heating. Thermotolerance manifests itself in different ways, depending on whether heat is given as a high temperature for a short time or as a lower temperature for many hours (as in the case in whole-body hyperthermia). The phenomenon is clearly of great clinical importance and is the subject of many detailed studies. Thermotolerance for heat as a potentiator of radiation damage appears to be much less than for direct heat injury, illustrating the difference in mechanism between heat enhancement of X-ray injury and direct heat damage.

If heat enhancement of radiation damage is greater for tumours than for normal tissues, there will be a therapeutic advantage in using a combined treatment. Thermal Enhancement Ratio (TER) does not vary widely between different normal tissues. It tends to be greater if heat is given before X-rays and also tends to be higher for larger doses per fraction. Unfortunately the possibility of a therapeutic gain has been difficult to ascertain experimentally owing to the problems of uniformly heating tumours (or even normal tissues). This has been illustrated in experiments on mouse tumours and on exteriorized mouse intestine.

The interaction between heat and X-rays fades when they are separated in time until it is lost by 4–5 h, as illustrated by many experiments on cells *in vitro* and on normal tissues. It is possible that a therapeutic advantage may be gained by heating 4 or 5 h after irradiation, the rationale for which will be discussed.

A range of techniques are now available for achieving local hyperthermia: ultrasound, micro or radiofrequency waves. The interaction of electromagnetic radiation with biological materials depends on the frequency, the method of application and on biological factors. Suitable non-invasive temperature measurements have not yet been developed and even invasive techniques are not simple. However, despite these difficulties, clinical results are extremely encouraging. Tumours in man and other large animals appear to become hotter than normal tissues, very few complications have been reported and a number of highly optimistic clinical reports are appearing in the literature

### TREATMENT OF ACUTE LEUKAEMIA BY WHOLE-BODY IRRADIATION AND MARROW TRANSPLANTATION: 25 YEARS FROM MOUSE TO MAN

P. ALEXANDER

*From the Institute of Cancer Research, Clifton Avenue, Sutton, Surrey, England*

NINETY PER CENT of patients with acute myelogenous leukaemia (AML) who have attained a complete clinical remission by treatment with chemotherapy relapse with and die of leukaemia. Thomas *et al.* (1979) at Seattle have shown that if patients in remission are given a single treatment of total body irradiation (TBI) at high dose (~10 Gy) together with a short course of chemotherapy, the incidence of relapse falls to less than 20%. This procedure requires that the lethally damaged marrow of the patient is restored by a transplant from a normal donor whose marrow, unlike the patient's, does not harbour leukaemia cells. The concept that leukaemia might be treated in this way arose from experiments carried out by Barnes *et al.* (1956) at the Radiobiological Laboratories at Harwell, which showed that a murine leukaemia could be eradicated by supralethal TBI (but not by lower doses which allowed some of the animals to survive the effects of irradiation) and that the mice could be rescued by a marrow graft. Twenty-two years have elapsed between these first animal experiments and the introduction of an acceptable clinical protocol. This was not because physicians were slow off the mark. Indeed, within one year of the Harwell publication, Thomas *et al.* (1957) reported on the problems encountered in trying the procedure in patients, and his group decided to abandon clinical trials in favour of an extensive laboratory programme of marrow transplantation in dogs exposed to TBI. In Paris, Mathé had similar dismal results in patients, but persevered until 1963 because allografts of marrow given in Paris were held responsible for saving the lives of 4 scientists who had in 1959 been accidentally exposed to high doses of irradiation in a reactor accident in Yugoslavia. Initially, they were thought to have received a lethal dose of radiation, but a careful reconstruction of the accident some years later suggested that the dose had been overestimated and their survival need not be attributed to the inoculation of marrow.

To reach the present state of clinical application, many problems had to be resolved which required careful investigative work, technological innovations (such as blood-cell separators to provide cells for support) and the development of new antibacterial agents. However, the major barrier to success and one often pronounced insoluble was graft-*versus*-host-disease (GvHD). Contemporaneous advances in histocompatibility typing allowed the Seattle group to select donor-recipient combinations in which GvHD could be prevented by cytotoxic drugs in half of the patients. The GvHD prophylaxis used at Seattle was based on a mouse model first described by Uphoff (1958), but it needed much development initially in dogs and then in man. Even so GvHD was associated with the 40% mortality which this treatment carries in the group of patients selected for treatment (*i.e.* young, in remission and with a matched donor).

The chance discovery by Sandoz in Switzerland of an antibiotic Cyclosporin A with a unique immunosuppressive action (Borel *et al.*, 1976) had a dramatic impact on GvHD. Work carried out in the last two years in England (Powles *et al.*, 1980) indicates that this new agent virtually eliminates GvHD as a clinical problem where the donor marrow is adequately matched, and may even make mismatches possible. To stop GvHD the patient has to be immunosuppressed, but most immunosuppressants interfere with cell division, and in marrow grafts can only be used at low doses if they are not to destroy the newly proliferating marrow. The reason for testing Cyclosporin A for marrow grafting was that it is immunosuppressive without being myelotoxic.

The end of the story has not yet been reached. Radiobiology in the early 1950's raises the spectre of numerous possible late effects, of which a wasting syndrome associated with progressive lymphoid atrophy may be the most worrying. There are many other challenging questions, such as whether an immunological mechanism contributes to the destruction of the leukaemia, or whether this is wholly due to TBI. Is AML uniquely radiosensitive or is it possible that other disseminated malignant diseases may be treated in the same way? Clinically, the procedure cannot yet be offered to the more elderly patients, and much attention will have to be devoted to reducing toxicity.

REFERENCES

Barnes, D. W. H. *et al.* (1956) *Br. Med. J.*, ii, 627.

Borel, J. F. *et al.* (1976) *Agents Actions*, **6**, 468.

Powles, R. L. *et al.* (1980) *Lancet*, ii, 1327 and (1980) i, 327.

Thomas, E. D. *et al.* (1957) *N. Engl. J. Med.*, **257**, 491.

Thomas, E. D. *et al.* (1979) *N. Engl. J. Med.*, **301**, 597.

Uphoff, D. E. (1958) *Proc. Soc. Exp. Biol. Med.*, **99**, 651.

### THE DEVELOPMENT OF INTERFERON AS AN ANTI-CANCER AGENT

N. B. FINTER AND K. H. FANTES

*From The Wellcome Research Laboratories, Beckenham, Kent, England*

INTERFERONS ARE members of a family of proteins with enormous biological activity (one antiviral unit corresponds to $10^{-8}$ mg) and a variety of effects on cell functions. Although discovered as long ago as 1957, their value in medicine largely remains to be established. Studies in the 1960's confirmed that appropriate interferons could protect animals against a wide variety of experimental infections with viruses, including known tumour viruses (Finter, 1973). Unexpectedly, it was found that the incidence or growth of carcinogen-induced, transplanted and spontaneously arising tumours of animals could also be influenced by interferon (Gresser & Tobey, 1978). The possible mechanisms involved in these anti-tumour effects will be discussed.

The evaluation of interferon in man has been frustrated by the shortage of suitable material. In consequence, it has so far only been possible to treat small numbers of patients in uncontrolled studies. Most of the interferon used has been made from buffy-coat cells collected from transfused blood. Some of the results obtained in cancer patients with the rather crude resulting product ($HuIFN_{\alpha}$) have been encouraging. Other sources of interferon for clinical use include human diploid fibroblasts (yielding $IFN_{\beta}$) and human lymphoblastoid cells (mainly $IFN_{\alpha}$); these latter can be used for production on a commercial scale. Genetic engineering techniques may provide limitless quantities of interferon proteins. It remains to be proved whether the resulting individual and non-glycosylated interferon proteins will mirror the effects of the mixture of proteins obtained from cultured human cells.

### REFERENCES

FINTER, N. B. (1973). *Interferons and Interferon Inducers*. Amsterdam: North-Holland. p. 295.

GRESSER, I. & TOBEY, M. G. (1978) *Biochim. Biophys. Acta*, **516**, 231.

### ABSTRACTS OF MEMBERS' PROFFERED PAPERS

**ADENOCARCINOMA OF THE OVARY —THE ROYAL MARSDEN HOSPITAL EXPERIENCE 1969–73.** A. VILARDO and and THE OVARIAN CANCER GROUP (V. M. Dalley, J. Baker, C. L. Harmer & E. Wiltshaw)

A review of 324 cases of adenocarcinoma of the ovary seen at the RHM between 1969 and 1973 is presented. Three treatment policies were followed for early-stage (I & II) disease: (a) no post-operative treatment, (b) pelvic radiotherapy, and (c) abdominopelvic radiotherapy. Overall 5-year survival was related to stage, and 69%, 39%, 3·7% and 0% were recorded for Stages I–IV respectively. Well differentiated tumours did better than poorly differentiated ones, and there was some indication that in Stages I and II abdominopelvic irradiation was better than pelvic irradiation alone, which in its turn was better than no post-operative treatment. This study describes the background for our present trials in all stages of the epithelial tumours of the ovary.

**PROGNOSTIC VARIABLES IN CARCINOMA OF THE OESOPHAGUS.** R. COX, C. E. NEWMAN, E. CLARIDGE & H. R. MATTHEWS, *Departments of Clinical Oncology, Medical Physics and Biomedical Engineering, Queen Elizabeth Hospital, Birmingham, B15 2TH, and Department of Thoracic Surgery, East Birmingham Hospital, Birmingham B9 5ST*

Fifteen variables were assessed at presentation in 100 consecutive patients with carcinoma of the oesophagus. These patients were then followed up for a minimum of one year to determine which of the variables are of prognostic significance.

Differences in median survival of >100 days were found with the following variables: complete resection *vs* all other treatments, the decision to explore or palliate, presence or absence of a coexistent medical condition, high or moderate physical status, absence or presence of a post-operative complication, minimal dysphagia *vs* moderate or severe, and the sex of the patient (median survival is longer in women).

Analysis of survival by the Life Table/Log Rank method showed the following to be highly significant variables ($P<0{\cdot}001$) operation, presence or absence of post operative complication, decision to explore or palliate, physical status and performance status. Significant variables ($0{\cdot}05>P>0{\cdot}001$) were coexistent medical condition, age, histology, weight loss, degree of dysphagia and presence of secondary spread. Not significant were sex, presence of painful dysphagia, duration of symptoms and the site of the tumour. Some of these variables are inter-related.

At present only 5% of all patients with carcinoma of the oesophagus survive 5 years. Chemotherapy may be of value in improving the prognosis and this will be tested in clinical trials. Design of such trials will require a knowledge of prognostic variables.

**ISOELECTRIC FOCUSING OF OESTRADIOL RECEPTOR PROTEIN FROM HUMAN MAMMARY CARCINOMA—A COMPARISON WITH THE DEXTRAN-COATED CHARCOAL ASSAY.** E. J. LLOYD, D. M. BARNES & L. G. SKINNER, *Clinical Research Laboratories, Christie Hospital and Holt Radium Institute, Manchester M20 9BX*

A laboratory method for the detection of oestradiol-17$\beta$ receptor protein ($RE_c$) in breast tumour tissue by isoelectric focusing (IEF) in polyacrylamide gel (Gustafsson *et al.*, 1978, *Cancer Res.*, **38**, 4225) was investigated. The results obtained by this method were compared to those from the dextran-coated charcoal method (DCC) (Korenman & Dukes, 1970, *J. Clin. Endocrinol*, **30**, 659). The estimation of $RE_c$ by IEF may have considerable clinical importance as $RE_c$ can be detected by this method in tissue samples $\frac{1}{10}$ the wet weight normally considered minimal for DCC analysis. The method may therefore be suitable for Tru-cut and Fine-needle biopsy samples.

A modified IEF method was used to investigate 32 cytosols prepared from unselected breast-tumour tissue. The results, when compared with DCC results obtained on the same cytosol, agreed on the presence or absence of receptors in 97% of cases. Quantitative analysis of the positive results showed a correlation coefficient of 0·88 ($P<0{\cdot}001$). However, IEF gave significantly lower receptor concentrations than the DCC method. Twenty-six Tru-cut biopsy tumour specimens were pre-incubated with radioactive oestradiol and analysed for receptor by IEF. The results were compared with those obtained by the DCC method on the same tumour. In 88% of the specimens examined both methods agreed as to the presence or absence of $RE_C$. A correlation coefficient, of 0·62 ($0{\cdot}01 < P < 0{\cdot}02$) was calculated from the 15 positive results. There was no significant difference between the results from the two methods. Quantitative results were related to total protein concentration in the cytosol. Negative receptor status was defined as $<5$ fmol/mg protein for both methods.

### CYTOSOL OESTROGEN AND PROGESTERONE RECEPTORS, NODE STATUS AND EARLY RECURRENCE OF PRIMARY BREAST CANCER.

J. M. T. Howat, M. Harris, R. Swindell & D. M. Barnes, *Christie Hospital and Holt Radium Institute and Withington Hospital, Manchester M20*

Recent papers have emphasized the value of the measurement of cytosol oestrogen receptors ($RE_C$) in primary breast tumours as a predictor of early recurrence, (Knight *et al.*, 1977; Maynard *et al.*, 1978; Cooke *et al.*, 1979); the clinical application of progesterone receptor ($RP_C$) estimations is less clear.

This is a preliminary report on $RE_C$ measured in tumour cytosols from 175 patients treated by modified radical mastectomy in whom there was histological proof of the presence or absence of axillary node involvement. At a median follow-up of 24 months (range 1–48 months) the occurrence rate was significantly lower in patients whose tumours were $RE^+$ than in those whose tumours were $RE^-$ ($P=0{\cdot}02$, 102E+ ; 73E−). There was no difference in the recurrence rates of $RP^+$ and $RP^-$ tumours. The overall recurrence rate was significantly higher in node-positive patients regardless of the receptor status ($P<0{\cdot}001$) none the less a significant improvement in disease-free interval was seen in $RE^+$ patients when node status was taken into account ($P=0{\cdot}045$). When analysed with regard to the degree of node involvement it was only those patients with few nodes (1–3) who benefited by having RE, for they had a significant improvement in the disease-free interval when compared with $RE^-$ patients ($P=0{\cdot}049$. 21+, 17−). No significant difference in recurrence rate was seen in node-negative patients ($P=0{\cdot}913$, 62+ ; 38−) nor in those with more than 4 axillary nodes ($P=0{\cdot}286$, 19+ ; 18−).

Although the follow-up is short this study confirms that measurement of RE may provide useful prognostic information, but they cannot be considered in isolation and the importance of axillary-node involvement should not be underestimated.

### ELASTOSIS IN HUMAN BREAST CANCER AND RESPONSE TO ENDOCRINE THERAPY.

J. R. W. Masters*, R. R. Millis†, R. J. B. King‡, M. Minton† & R. D. Rubens†, **Institute of Urology, St. Paul's Hospital, London, WC2, †Imperial Cancer Research Fund Breast Cancer Unit, Guy's Hospital, London, SE1 and ‡ICRF, Lincoln's Inn Fields, London, WC2*

The amount of elastosis in primary breast tumours was found to be associated with response to endocrine therapy in a preliminary study of a small series of cases (Masters *et al.* (1979) *Br. J. Cancer*, **39**, 536). In order to confirm this finding in histological sections from the primary tumours of 245 patients with advanced breast cancer, elastosis was subjectively assessed as absent, present in small or moderate amounts, or present in gross amounts. The patients received a total of 289 endocrine treatments assessable using UICC criteria (oophorectomy, 38; ovarian irradiation, 22; androgens, 45; oestrogens, 37; hypophysectomy, 32; tamoxifen, 115). In the women whose tumours did not contain elastosis there were 8/56 (14%) responses, in contrast to 13/31 (42%) in those with gross elastosis (see Table). The assessment of elastosis is a simple exercise which requires no additional material, and these data confirm that there is an association between elastosis in primary breast tumours and the response to endocrine therapy for advanced disease.

| | Amount of elastosis | | |
|---|---|---|---|
| Response | None | Present | Gross |
| Complete | 0 | 8 | 4 |
| Partial | 8 | 59 | 9 |
| No change | 7 | 17 | 3 |
| Progressive disease | 41 | 118 | 15 |
| Total | 56 | 202 | 31 |

### IS THERE POLYMORPHIC METABOLISM OF AMINOGLUTETHIMIDE IN MAN?

S. J. HARLAND, R. C. COOMBES, M. JARMAN, E. C. NICE & A. B. FOSTER, *Institute of Cancer Research and Ludwig Institute of Cancer Research, Sutton, Surrey*

Aminoglutethimide (AGT) inhibits the synthesis of corticosteroids in man and is used in advanced breast cancer as a means of achieving a medical adrenalectomy. Since a principal metabolic reaction for this compound is N-acetylation (Douglas & Nichols, 1972, *J. Pharm. Pharmacol.*, **24**, Suppl. 150) it is of interest to determine whether there is a bimodal distribution for the rate of this pathway in the population as is the case with a number of other drugs which are N-acetylated. Nine fasting volunteers, 5 rapid and 4 slow acetylators, took a 250 mg tablet of AGT, gave blood samples and collected urine for the next 24 h. Analysis of plasma for AGT and its N-acetyl derivative by HPLC revealed (1) no difference between the two groups in the levels of the parent compound at 0·5, 2 and 8 h after its administration, and (2) the levels of N-acetyl AGT were significantly higher in the rapid acetylators at the 3 times (means of 1·06, 1·22 and 1·05 $\mu$g/ml, respectively). The clinical importance of this finding is being currently investigated. Other studies have shown that there is no stereoselective metabolism of the enantiomers of AGT. Nitroglutethimide has been identified as a urinary metabolite by GC-MS. This metabolite may be of toxicological significance.

### CLINICAL EXPERIENCE WITH ORAL HIGH-DOSE METHOTREXATE.

D. J. FORECAST, B. MUNDAY & A. HOWELL, *Departments of Medicine and Clinical Pharmacology and Therapeutics, University of Birmingham, Edgbaston, Birmingham*

Oral methotrexate MTX may give comparable concentration–time curves to infusions if the dose is subdivided. It may be given on an outpatient basis, thus saving the expense of hospital admission and patient time. (Bell *et al.*, 1978 *Br. Med. J.*, i, 857). Since tumours may recur between courses of chemotherapy, the aim of this study was to test the safety and absorption of oral MTX given to out-patients between conventional courses of i.v. chemotherapy. Twenty-seven patients (9 non-Hodgkin's lymphoma (NHL), 5 bladder, 7 teratoma, 5 gynaecological malignancy and one head and neck) were given a total of 125 courses. Multiple blood samples were taken over 48 h to test absorption during 20 of these. MTX regimes were, 8 × 50 mg 2-hourly, or 4 × 100 mg 5-hourly, or 4 × 50 mg 5-hourly, all with leucovorin rescue at 24 h (6 × 15 mg 6-hourly).

There was no systemic toxicity or treatment delay due to haematological toxicity in patients with NHL (CHOP, MTX given on Day 14 of 28-day cycle), bladder carcinoma (*cis*-Pt, MTX on Day 10 and 17 of 21-day cycle) or amongst 7 patients given MTX alone (one NHL, 5 gyn., 1 head and neck). However, there were 5 episodes of oral ulceration and 7 of low white count ($4{\cdot}0 \times 10^9/l$) in 9 of the 47 courses given to teratoma patients on *cis*-Pt and VP-16 over 5 days with MTX on Day 10 and 17.

Areas under curves for the 3 dose schedules were: 8 × 50 mg, 26,023 ± 6876 ng. h/ml (n = 6); 4 × 100 mg, 24,624 ± 9195 ng. h/ml (n = 5) and 4 × 50 mg, 14,391 ± 7644 ng. h/ml (n = 5). Two patients sampled during several cycles showed no appreciable fall off in absorption with time. We conclude that the toxicity of oral high-dose MTX is acceptable, and that absorption occurs between cycles of i.v. chemotherapy.

### HIGH-DOSE MELPHALAN IN ADVANCED MALIGNANT MELANOMA.

G. J. S. RUSTIN, M. A. CORNBLEET, D. W. HEDLEY & T. J. MCELWAIN, *Department of Medicine, The Royal Marsden Hospital and Institute of Cancer Research, Downs Road, Sutton, Surrey, England*

Twenty-seven patients with symptomatic or life-threatening metastases from malignant melanoma were treated with high-dose melphalan (> 140 mg/m$^2$) and autologous marrow rescue. Fourteen of these patients also received cyclophosphamide (500 mg) i.v. 7 days before the melphalan. These manoeuvres reduced the gut and marrow toxicity of the melphalan, so that doses of up to 260 mg/m$^2$ could be tolerated. Objective tumour regression was obtained in 20/27 (74%) of the patients with 12/27 (44%) showing a decrease in the product of 2 diameters of all measurable lesions of > 50% of whom 2/27 (7%)

entered complete remission. This compares with a regression rate of only 9% when conventional doses of melphalan are used (Luce, 1972, *Cancer*, **30**, 1604). Overall there was no survival benefit for those whose tumours regressed compared to those with no response. However, life-table analysis showed that median survival for the 9 patients who received $>200$ mg/m$^2$ of melphalan having previously been primed was 11 months from treatment and 14 months from dissemination, compared with 3 and 8 months respectively for those who received $<200$ mg/m$^2$ of melphalan.

Although survival from dissemination in this treated group is longer than in other published series, we would not advocate high-dose melphalan in melanoma except as palliation in patients with severe symptoms. However, the response rate and survival data do suggest that this treatment might form the basis of combination therapy. We are at present investigating its combination with misonidazole, and its use in the treatment of other tumours.

### THE PATHOGENESIS AND CONTROL OF INTERFERON-INDUCED PYREXIA.

T. J. Priestman & R. Lucken, *Wellcome Research Laboratories, Beckenham, Kent*

Recent papers have reported the use of leucocyte and fibroblast interferon (IFN) in the treatment of advanced cancer. In all studies, pyrexia was seen after initial doses of IFN. The material used in these trials was only 0·1–1% pure, and it has been suggested that fever was caused by non-IFN protein. In a study with lymphoblastoid IFN (HLBI), some 20–50 times purer than IFN used in previous clinical series, pyrexia was initially dose-limiting (Priestman, 1980, *Lancet*, ii, 113). The possibility of endotoxin contamination was excluded by negative *Limulus* lysate assays, chick-embryo toxicity, and rabbit pyrogenicity tests. The clinical and laboratory data indicated that IFN-induced fever was mediated by the release of endogenous pyrogen. A subsequent clinical trial showed that pretreatment with salicylates reduced the degree of fever after HLBI administration. It is concluded that IFN is inherently pyrogenic, causing the release of endogenous pyrogen, and that salicylate therapy will reduce the fever until tolerance to this phenomenon develops 4–5 days after starting IFN treatment.

### HIGH-DOSE COMBINATION CHEMOTHERAPY FOR NON-HODGKIN'S LYMPHOMA OF UNFAVOURABLE HISTOLOGICAL TYPE.

R. Bell, C. J. Gallagher, J. Ford, J. S. Malpas & T. A. Lister, *ICRF Department of Medical Oncology, St. Bartholomew's Hospital, London EC1*

Twenty-one consecutive untreated patients with non-Hodgkin's lymphoma (NHL) of unfavourable histology were treated between May 1978 and August 1979. Fifteen males and 6 females, median age 55 years old (range 18–70) were of the following histological groups (Rappaport) diffuse histiocytic 15, diffuse undifferentiated 6, immunoblastic 8, lymphoblastic 3, centroblastic 3, malignant histiocytosis 3 and other high-grade 4. All but one had Stage IV disease with a mean of 2·2 sites of extranodal involvement per pt. These included: liver 10 pts, marrow 7, gastrointestinal 7, lung 5, bone 4, soft tissues 4, testes 2, pericardium 2, urinary tract 2 and oropharynx 1, superior venacaval obstruction 4 pts. High-dose combination chemotherapy was given as cyclophosphamide 2·5 g/m$^2$ i.v., Adriamycin 50 mg/m$^2$ i.v., vincristine 2 mg i.v., methotrexate 12·5 mg i.t. and daily prednisolone 1 g/m$^2$ i.v. $\times 5$ (CHOMP), repeated every 3–4 weeks for a minimum of 6 courses. Ten pts received full doses and 11 reduced doses, because of haematological and gastrointestinal toxicity. There were 7 responders (33% overall) of which 4 were complete responses, 2 partial responses with minimal X-ray abnormalities and one failure who entered CR after cranial and abdominal radiotherapy. All responders are alive without relapse for a mean period of 17·0 months (range 12–24·7) with a mean duration of remission of 10·6 months (range 6·2–14). Six pts failed to respond and died with resistant disease and infection. Four pts died of infection during remission induction with responding disease, and four others died in their first cycle of treatment, one of infection, one of gastric haemorrhage, one of myocardial infarction and one of severe metabolic disturbance. A more intensive programme has doubled the survival (33%) of our Stage IV unfavourable histology pts (Lister *et al.*, 1978, *Cancer Chemother. Rep.*, **1**, 107).

**TUMOUR IMPLANTS IN MOUSE EARS: A MODEL FOR LATE IRRADIATION EFFECTS ON TUMOUR VASCULATURE.** J. SHEWELL, *Radiobiology Department, St Bartholomew's Hospital Medical College, London*

The thinness of the mouse ear allows direct observation of its blood vessels, a property used in studying vascular damage after irradiation (Bakowska & Lindop, 1971, *6th Eur. Conf. Microcirculation*, p. 145; Stearner *et al.*, 1976, *Rad. Res.*, **65**, 351). The developing vasculature of tumour implants of C3H/Bts mammary tumours in one ear of C3H/Bts male mice can also be followed. Late radiation fibrosis of the ear vessels might impair post-irradiation administration of cytotoxic drugs. Using this model, it has been shown that 80 mg/kg methotrexate i.v. produces a tumour response (Shewell, 1976, *Br. J. Cancer*, **33**, 210) of 0·6 in both non-irradiated and irradiated tumours up to 6 mths after irradiation. After this time, and proportional to irradiation dose (5–25 Gy 220 kVp X-rays) i.v. methotrexate is increasingly ineffective on irradiated implants. This timing coincides with the development of morphological vascular abnormalities in the irradiated non-tumour ear.

**INHIBITION BY MATURING GRANULOCYTES OF THE REGENERATION OF HAEMOPOIETIC PROGENITOR CELLS IN MICE TREATED WITH COMBINATIONS OF CYTOTOXIC AGENTS.** N. M. BLACKETT, *Biophysics Division, Institute of Cancer Research, Sutton*

Studies on the response of haemopoietic tissue in mice to cytotoxic agents in combination with whole-body irradiation have shown that the number of maturing granulocytic cells in the marrow has a large inhibitory effect on the regeneration of the early progenitor cells. A similar effect is observed with the regeneration of normal marrow cells injected after the irradiation, indicating that the effect is the result of a change in the stimulus for marrow regeneration.

With 3 cytotoxic agents, cyclophosphamide, cytosine arabinoside and 5-fluorouracil, there is the same high inverse correlation between the number of granulocytes with ring-shaped nuclei at the time of irradiation, and the subsequent regeneration of stem cells. It seems likely that these effects are due to the operation of an important biological regulatory mechanism of haemopoietic stem cells that can be manipulated in ways that could improve marrow transplantation and chemotherapy.

**RELATIONSHIP BETWEEN ABILITY OF TUMOUR CELLS TO AGGREGATE PLATELETS AND THEIR CAPACITY TO SEED OUT OF THE CIRCULATION.** N. WILLMOTT, A. MALCOLM* & K. C. CALMAN, **Department of Oncology and Pathology, University of Glasgow*

Two murine tumours, the TLX-5 lymphoma and Sarcoma 180, were examined for their capacity (a) to aggregate circulating blood paltelets as measured by the capacity of i.v. injected tumour cells to elicit a drop in platelet count 20 min after injection and (b) the anatomical distribution of tumour deposits in mice sacrificed when the morbid effects of an i.v. injection of tumour cells became evident. It was found that administration of up to $2 \times 10$ TLX-5 lymphoma cells failed to elicit platelet aggregation, and at necropsy tumour deposits were detected histologically in spleen, liver and kidney. In contrast, $1{\cdot}5 \times 10^6$ *in vivo* grown Sarcoma 180 cells elicited platelet aggregation and at necropsy tumour deposits were detected only in the lungs. If, however, Sarcoma 180 cells were grown in tissue culture they lost their ability to aggregate platelets and also their capacity to form tumour deposits after i.v. injection. These observations are consistent with the hypotheses that (a) platelet aggregation is important in the seeding out of tumour cells from the circulation and (b) tumour cells that are not adapted for existence in the asocial environment of the blood stream may be destroyed if confined there.

**LECTIN AGGLUTINABILITY OF MAMMARY TUMOURS WITH DIFFERING METASTATIC COLONIZATION POTENTIALS.** J. E. PRICE & D. TARIN, *Department of Histopathology, John Radcliffe Hospital, University of Oxford*

Lectin agglutinability has been used in this investigation as one means of comparing the surface composition of cells from tumours with high and low pulmonary colonization potential. As in our previous work, we have used only primary (*i.e.* naturally occurring)

mammary tumours in mice. These were disaggregated with collagenase to obtain a single-cell suspension and the metastatic colonization potential assessed by reinoculation *via* the tail vein into the donor and batches of 5 syngeneic animals. Aliquots from the same cell suspension were studied for lectin agglutinability on the same day. Debris and red cells were removed from the aliquots by density-gradient centrifugation on Percoll, and the rate and degree of agglutinability assessed by measurement of the disappearance of single cells from suspension with a Coulter counter linked with a C-1000 channelizer. It was found that agglutinability with the lectins Concanavalin-A and wheatgerm agglutinin bore no relationship to the pulmonary colonization potential of the primary mammary tumour. However, studies on disaggregated secondary deposits from tumours which manifested high colonization potential were consistently less agglutinable than the cells of the primary tumours from which they were originally taken. Further investigation is now in progress to examine whether this difference is due to a selection process during dissemination or to site-induced changes in the cells which lodge in the lung.

**METASTATIC PROPERTIES AND CELL SURFACE GLYCOPROTEINS OF A HAMSTER TUMOUR OF HSV AETIOLOGY AND ITS TWO METASTATIC SUBLINES.** R. C. Rees & J. R. Walker, *Department of Virology, The University of Sheffield Medical School, Sheffield*

The acceptance of a realistic animal model for human malignancy requires that the tumour should not be highly antigenic or encapsulated, but that cells should spontaneously metastasize to sites distant from the primary solid mass. Two such metastatic sublines have been isolated from a hamster tumour of HSV-2 aetiology (333–2–26). These metA and metB lines were established by *in vivo* s.c. transplantation of lung metastatic foci which occurred at low frequency in hamsters after resection of the primary tumour. Both lines were considerably more metastatic than the parent; hamster mortality due to metastasis was typically 100% 40 days after tumour resection compared with 20% in the parent line.

The efficiency of plating in soft agar of the parental and metA and metB lines was assessed; no correlation between anchorage-independent growth and metastatic potential was seen. In addition, the cell-surface glycoprotein profile was analysed by PAGE after labelling by pretreatment with galactose oxidase or periodate and borotritiide reduction. Development of metastatic potential was correlated with an increase in high-mol.-wt species of surface glycoproteins possessing a terminal neuraminic acid and subterminal galactose residue.

**CELLULAR BASIS FOR THE LOSS OF CARCINOGEN FROM METHYLCHOLANTHRENE - IMPREGNATED MILLIPORE DISCS.** M. F. A. Woodruff, J. Bard, A. Ross & G. Forbes, *MRC Clinical and Population Cytogenetics Unit, Western General Hospital, Edinburgh EH4 2XU*

Millipore discs impregnated with methylcholanthrene (MC) implanted s.c. in mice, evoke an early intense macrophage and giant cell reaction. Later, discs become covered with connective tissue, and eventually fibrosarcomas develop. Studies with $^3$H-labelled material show that MC is removed from the discs and rapidly broken down (half life about 7 days) to a water-soluble product which is excreted in faeces and urine. Removal of label is halted by whole-body irradiation (5·5 Gy) with the disc area shielded; this observation, in conjunction with the histological and autoradiographic findings, and the paucity of label in cells stripped from excised discs, suggests that the removal and degradation of MC depends on macrophages and the replacement of spent macrophages by new cells generated centrally. The rate of disappearance of label from implanted $^3$H-MC discs was not altered by administration of *Corynebacterium parvum* (CP); this however does not exclude the possibility that the metabolic pathways involved in the removal of MC are altered. To investigate this it is proposed to study, in both normal and CP-treated mice, the extent to which cytochrome P450 and other enzymes concerned in the activation and detoxification of polycyclic hydrocarbons by liver microsomal fractions are inducible in the macrophages which accumulate in inflammatory exudates. The histological techniques used should be readily applicable to the study of the early stages of chemical carcinogenesis and the host reaction to transformed cells.

**OBSERVATIONS UPON THE DISTRIBUTION OF MAST CELLS IN LYMPH NODES, DURING THE PROGRESSION OF A LYMPHOID LEUKAEMIA OF THE RAT.** S. T. QAZZAZ & R. W. STODDART, *Department of Experimental Pathology, Stopford Building, University of Manchester, Manchester M13 9PT*

Few previous studies have been made of the distribution and numbers of mast cells in lymph nodes in relation to normal and pathological states. In normal human and rat nodes, most mast cells are found either at the margins of the medulla and paracortex (MMC) or at the outer edge of the node (CMC) the minority being in the intervening paracortex (PMC). They are rare in follicular areas.

During the progression of the Roser leukaemia in the rat, the MMC disappear before the lymph nodes are enlarged, or are histologically abnormal, while the PMC are lost during infiltration and enlargement. The CMC are eventually reduced to those remaining in the subcapsular sinus. After an early decline, total mast cell numbers rise again in the terminal stages of the disease.

The results support the view that mast cells migrate centripetally across lymph nodes, though the early disappearance of the MMC is unlikely to be caused by mechanical obstruction of the node by proliferating leukaemic cells.

**EXTRAVASCULAR NATURAL CYTOTOXICITY IN MAN: STATUS OF LYMPH NODE AND TUMOUR-INFILTRATING LYMPHOCYTES.** M. MOORE & B. M. VOSE, *The Paterson Laboratories, Christie Hospital and Holt Radium Institute, Manchester 20*

A major distinction is reported between the cytolytic activity of peripheral-blood lymphocytes (PBL) and that of lymph-node and tumour-infiltrating lymphocytes (TIL) against targets of the NK-sensitive K562 cell line in short-term $^{51}$Cr-release assays. In the PBL of normal donors and lung-cancer patients in whom disease was advanced, anti-K562 reactivity, though variable, was consistently detectable, and this activity could be augmented to a similar extent in patients and controls by treatment of effectors with exogenous interferon (IF). By contrast, anti-K562 activity in lymph-node cells (LNC) and TIL was virtually absent, and significant levels could not be induced by exposure to IF. This activity was not attributable to co-existent suppressor cells for NK function, since admixture of LNC and TIL with normal PBL failed to modulate K562 killing by the latter. The results imply that K562-reactive NK cells and their precursors may frequently be present at sub-threshold levels in the lymph nodes of tumour-bearing patients, and a similar explanation could account for the inactivity of TIL. However, in the latter situation, actively-induced NK dysfunction *in situ* incapable of regeneration by IF, and attributable to presently undefined tumour-associated factors, may also be involved.

**ROLE OF MINOR TRANSPLANTATION ANTIGENS IN CONTROLLING T-CELL RESPONSES TO H-Y.** W. FIERZ, M. BRENAN, A. MÜLLBACHER & E. SIMPSON, *Transplantation Biology Section, Clinical Research Centre, Harrow, Middlesex*

Failure to activate the immune response may account for the growth of some tumours. Some (*e.g.* those caused by MSV virus in mice) are rapidly rejected with concomitant production of H-2-restricted cytotoxicity. Others, including "spontaneous" tumours of unknown aetiology are rarely rejected. We have used the male-specific antigen, H-Y, as a model for tumour antigens. Female mice of some strains are responders, rejecting syngeneic male skin and/or generating H-2 restricted cytotoxic cells (Hurme *et al.*, 1978, *J. Exp. Med.*, **147**, 758; Müllbacher & Brenan, 1980, *Nature*, **285**, 34). Mouse strains of the H-2 haplotype can produce good cytotoxic responses (CBA), a more variable response (C3H, B10.BR) or no response (AKR) (data to be presented).

The difference in responsiveness within H-2$^{k}$ strains must therefore be due to one or more of the non-H-2 genes at which they differ. We are presently testing the possible role of minor transplantation antigens.

**LYMPHOCYTES MAINTAINED IN T-CELL GROWTH FACTOR (INTERLEUKIN 2) SHOW ENHANCED CYTOTOXICITY FOR HUMAN TUMOUR CELLS.** B. M. VOSE & M. MOORE, *The Paterson Laboratories, Christie Hospital and Holt Radium Institute, Manchester M20 9BX*

Conditions for the production of supernates from mitogen-stimulated human lymphocytes with the capacity to induce proliferation in long-term MLC or PHA cultures which do not respond to PHA have been investigated. These supernates can be used to maintain lymphocytes in continuous growth with a doubling time of ~48 h. Cells grown from MLC-stimulated cultures show >80% SRBC-rosetting cells, are $Fc^-$, do not show ADCC activity and have reduced lysis of K562 compared with freshly isolated effectors. Cultured T cells (CTC) show high lytic activity against the inducing PHA blasts but not against autologous or third-party targets. Similar experiments have been performed with lymphocytes from the blood, lymph node, spleen and tumour of cancer patients, and CTC tested for cytotoxicity against autologous and allogeneic tumour cells and the K562 cell line. Cytotoxicity for autologous tumour was found in all samples. This was accompanied by killing of allogeneic cells in most instances, but killing of K562 was only rarely demonstrable. These data would be consistent with a polyclonal expansion of cytotoxic effectors in the samples. The finding of autologous reactivity suggests the presence of autorecognitive cytotoxic T cells in cancer patients with specificity for tumour. Cloning experiments are currently in progress to investigate this possibility further.

**GROWTH OF HUMAN BLADDER-TUMOUR CELL LINES IN AN IMMUNE-DEPRIVED MOUSE HOST.** K. M. JOY*, R. J. BERRY*, & R. M. HICKS*, **Department of Cell Pathology and *Department of Oncology, Middlesex Hospital Medical School, London W1P 7LD*

Mice immunologically deprived by thymectomy, cytosine arabinoside pre-treatment and whole-body irradiation are known to be receptive to xenografts from an established laboratory cell line (Steel *et al.*, 1978) and human tumour biopsy tissue (Selby *et al.*, 1980). Such immune-deprived mice have been investigated as a potential xenotransplantation model for neoplastic human bladder tissue, for use in subsequent studies on neoplastic transformation *in vitro*.

Four established tumour cell lines derived from different transitional-cell carcinomas of the human urinary bladder were xenografted: EJ, RT112, T24, and RT4. The tumour cells were injected s.c. at the rate of $10^6$ cells per mouse into both immune-deprived mice and non-deprived controls. EJ cells rapidly produced tumours in 10/11 mice, while 8/10 mice that received RT112 cells developed tumours. The T24 cell line produced tumours in 10/20 mice, and on transplantation of solid tumour tissue from one of these animals 11/12 mice developed tumours. Tumours arose in 4/6 mice injected with RT4 cells, one of which was later transplanted into 10 mice with an 80% incidence of tumour formation. With all the cell lines tumours were generally detectable within 10 days of xenografting, and viable tumours were maintained in immune-deprived mice for over 120 days. No tumour was produced in any of the immunologically competent control mice.

The *in vivo* growth of xenografted cells was assessed by histopathological and electron microscopical techniques. RT112, T24 and RT4 tumours displayed a pseudopapillary growth pattern, while EJ tumours were poorly differentiated and had a solid pattern of growth. Tumours from all 4 cell lines showed similar growth. They exhibited progression from an encapsulated stage through capsule infiltration to malignant invasion. This immune-deprived mouse model thus appears to be a satisfactory alternative to the nude mouse as a host for human bladder tumour-cell lines.

**THE GROWTH OF HUMAN PANCREATIC TUMOURS IN ATHYMIC NUDE RATS *rnu/rnu*.** G. DAVIES, D. DUKE, A. G. GRANT, S. A. KELLY & J. HERMON-TAYLOR, *Department of Surgery, St George's Hospital Medical School, London, SW17 0RE*

The athymic nude rat (*rnu/rnu*) has been successfully established as an *in vivo* model for the growth of pancreatic exocrine tumours derived from a pancreatic exocrine adenocarcinoma maintained in cell culture. S.c. injections of $3 \times 10^7$ cells into the anterior chest wall of 25 animals yielded 21 tumours (84%). The critical period for the establishment of growth was between 28 and 42 days. Each tumour grew to a size of 400–600 $cm^2$. Tumours were successfully transplanted into further rats, and one pancreatic xenograft was passaged into nude mice (*nu/nu*) and transplanted back into nude rats; the take rate increased to 90%. Distant metastases did not occur but when tumours were excised surgically from nude rats, regrowth of the primary tumour occurred at the original site. The xenografts maintained the histological and cytological characteristics of the original tumour from which the cell line was derived; the karyotype of the cell line was also maintained in the solid tumour after passage and transplantation. Successful xenografting of 3 out of 4 primary explants from patients with pancreatic cancer is also reported. The athymic nude rat is a valuable complementary tool to the nude mouse for the study of pancreatic and other common solid human tumours, and for surgical and serial serological studies.

### LIMITATIONS OF HUMAN TUMOUR XENOGRAFTS IN INDIVIDUAL PATIENT DRUG-SENSITIVITY TESTING.

M, Bailey, A. Jones, D. Raghavan, P. Selby, A. Shorthouse, J. Gibbs & M. Peckham, *Institute of Cancer Research, Ludwig Institute for Cancer Research and Royal Marsden Hospital, Sutton, Surrey*

The clinical applications of the human tumour xenograft model are not yet clearly defined. it has been suggested that this system might provide a means of testing the drug sensitivity of an individual patient's tumour (*The Nude Mouse in Experimental and Clinical Research*, Academic Press, pp. 450 and 293). We have reviewed our data on xenografts of mammary, bronchial and ovarian carcinoma, malignant melanomas and testicular teratomas to assess the practicality of this suggestion.

The table shows the small number of patients in whom drug-sensitivity testing could have been performed before the patient's demise. The reason for this low figure is that patients with aggressive tumours had a high xenograft take rate, but died before testing could be performed, whereas patients with less aggressive tumours who survived long enough for drug testing to be possible had a low xenograft take rate. We conclude that xenografted tumours are not of practical value for individual patient drug testing.

| Tumour type | Total viable spec. implanted | Viable spec. from untreated patients | Serially transplantable takes | Usable takes (%) |
|---|---|---|---|---|
| Breast | 91 | 91 | 9 | 6 (6·6) |
| Teratoma | 46 | 34 | 11 | 7 (20) |
| Ovary | 37 | 20 | 7 | 2 (10) |
| Bronchus | 49 | 48 | 38 | 5 (12) |
| Melanoma | 16 | 8 | 10 | 4 (25) |
| Overall | 239 | 201 | 75 | 24 (12) |

### *IN VITRO* CYTOTOXIC DRUG SENSITIVITY TESTING OF HUMAN TUMOUR XENOGRAFTS GROWN AS MULTICELLULAR TUMOUR SPHEROIDS.

A. C. Jones*†, I. J. Stratford*, P. A. Wilson* & M. J. Peckham*†, **Institute of Cancer Research*, †*Royal Marsden Hospital, Sutton, England*

Using a sequential static-spinner culture system, an attempt has been made to initiate multicellular tumour spheroid formation from 13 different xenografted human tumours. All the tumours used had been established in immune-suppressed mice and were taken from the second passage onwards.

The range of tumours examined includes melanomas, ovarian, bronchial and breast carcinomas and a teratoma. Data on the colony-forming ability in soft agar of cells obtained from disaggregated spheroids have been obtained in 3 tumours. There appears to be a marked enhancement of the colony-forming ability of these cells, compared with the cells obtained directly by disaggregation of the tumour from the mouse. Individually growing spheroids obtained from 4 different tumours have a range of volume-doubling times of 2·4–15 days. Cytotoxic drug testing measurements of growth delay and clonogenic survival have been obtained with spheroids from a bronchial and a breast carcinoma.

These results support the idea that multicellular tumour spheroids may be useful as a solid-tumour model *in vitro*, suitable for testing cytotoxic drug sensitivity.

### MONOCLONAL ANTIBODIES FROM MICE BEARING HUMAN TUMOUR XENOGRAFTS.

H. Warenius* & K. Sikora†, **Department of Radiotherapy, Newcastle General Hospital, Newcastle-upon-Tyne and †MRC Clinical Oncology and Radiotherapeutics Unit, The Medical School, Cambridge*

We have examined the serological response in immunosuppressed mice bearing xenografts of the human colorectal-carcinoma line HT29R. The serum of such animals contains antibodies which bind to HT29R cell-membrane components. This antibody activity cannot be completely absorbed by either human lymphocytes or erythrocytes, indicating that it may be at least in part directed against tumour-specific antigens. By fusing spleens from tumour-bearing animals to an established myeloma line we were able to produce 14 monoclonal antibodies which bound to HT29R. Three of these have been found to bind specifically to HT29R membranes. A further 4 bind to both HT29R and other colorectal-carcinoma membranes. Two of these also bound to a carcino-embryonic antigen preparation. The remaining 7 bound to membranes from normal red cells, lymphocytes and colon, as well as HT29R. The serum of xenograft-bearing mice was examined for blocking activity against the specific anti-HT29R antibodies. This provides a possible method of measuring tumour load.

### SERUM FERRITIN AS A THIRD MARKER IN GERM-CELL TUMOURS.

A. Grail, G. Bates, A. Milford-Ward & B. W. Hancock, *University Department of Medicine and Immunology, Royal Hallamshire Hospital, Sheffield.*

Serial measurements of serum ferritin have been assessed as an additional diagnostic and prognostic marker in a study of 12 patients with germ-cell tumours. The standard markers, serum AFP and βhCG, were also assessed serially.

A poor prognosis was associated with persistently high levels of serum ferritin and either, or both, high AFP and βhCG levels. Falls and maintenance of normal levels of serum ferritin indicated favourable response to treatment; rising values were associated with recurrence of dissemination of tumour. The metabolic rates of decay of AFP and βhCG give valuable indications of residual tumour, when values exceed the normal half-life values of $<5$ days for AFP and $<24$ h for βhCG.

Although AFP and βhCG are useful as markers in the follow-up of patients, increasing levels suggesting recurrent tumour, falls to within the normal range do not necessarily indicate a disease-free state. Chemotherapy may convert the tumour to a non-marker-producing form and/or may selectively eradicate the clone of marker-producing cells. Due to heterogeneity in metastases, production of tumour markers from different sites may vary.

Possible explanations for the high serum ferritin level in malignancy include reticulo-endothelial block of iron release, tissue damage and increased synthesis by the tumour. Even if serum ferritin cannot be classed specifically as a tumour product, it is useful in the early detection of recurrent tumour and extragonadal progression of disease.

### LOCALISATION OF HUMAN TUMOURS BY EXTERNAL SCANNING AFTER INJECTION WITH RADIO-LABELLED ANTIBODY TO AFP.

D. S. Fairweather, A. K. Halsall, K. R. Hine, A. Howell, P. W. Dykes*, A. R. Bradwell, J. Blackburn & A. Reader, *Departments of Medicine, Oncology, Immunology and Medical Physics, Queen Elizabeth Medical Centre and *The General Hospital, Birmingham*

Alpha-foetoprotein (AFP) is used as a tumour marker in germ-cell tumours. This preliminary study was carried out to assess whether deposits of these tumours could be identified by external scanning after injection of radio-labelled antibody to AFP. The antibodies to AFP were raised in sheep by immunization with AFP extracted from amniotic fluid. After immunoadsorption with normal sera the IgG was further purified by ion-exchange chromatography. Samples were tested for acute toxicity and pyrogenicity in rabbits. The purified antibody was labelled with $^{131}I$ using the Chloramine-T method, and injected into the patients after an initial uneventful test dose. Prior to the scan, the patient also received $^{99m}Tc$-pertechnetate and $^{99m}Tc$-labelled human serum albumin to outline the extra vascular space and blood pool, to allow subtraction scanning.

A total of 15 scans were performed on 12 patients, 10 with malignant teratoma, one with an endodermal sinus tumour and one with a seminoma (used as a negative control). All 9 patients with high serum AFP gave positive scans; 5 of these at the time of diagnosis. All abnormal areas were confirmed by CT scans and lymphangiography, although 4 secondary deposits were missed.

Three patients gave normal scans when studied again after successful treatment, and when the serum AFP had fallen to normal. A further 5 studied for the first time after treatment (4 with high serum AFP) gave positive scans, 3 of which were confirmed by CT scanning. Two areas of anti-AFP uptake over the stomach were not confirmed by the CT scans.

These preliminary results show that it is possible to identify tumour deposits using antibody to AFP. Further work is needed, to establish whether this technique will aid the staging and management of these treatable tumours.

### DETECTION OF MAMMARY-CARCINOMA CELLS IN MARROW USING ANTISERA TO EPITHELIAL-MEMBRANE ANTIGEN.

D. P. Dearnaley*, J. P. Sloane†, M. G. Ormerod‡, K. Steele‡, R. C. Coombes* & A. M. Neville*, **Ludwig Institute for Cancer Research*, *‡Institute of Cancer Research and †Royal Marsden Hospital, Sutton, Surrey*

We have previously described an epithelial-membrane antigen (EMA) which can be demonstrated by immunohistochemical methods on formalin-fixed, paraffin-embedded sections of human tissue (Heyderman *et al.*, 1979, *J. Clin. Pathol.*, **32**, 35). EMA is confined to but widely distributed in epithelial tissues and tumours derived from them (Sloane & Ormerod, *Cancer*, in press) and has been used to detect micrometastases in histological section (Sloane *et al.*, *Br. J. Cancer*, in press).

We have developed a technique using an alkaline phosphatase conjugate for staining smears of marrow. Giemsa, Luke's and anti-EMA preparations were made from 74 posterior-iliac-crest marrow aspirates from patients with breast cancer. Anti-EMA staining revealed malignant cells in 15 patients, compared with 7 demonstrated by conventional methods. The 5 patients with $>100$ EMA-positive cells/smear were all diagnosed as malignant on Giemsa or Luke's preparations, but only 2/12 with $<100$ positive cells/smear were detected. Ten/24 patients with bone metastases had EMA-positive smears, as did 4/20 patients with disseminated disease but without bone involvement. Twenty patients had apparently localized primary breast cancer, one of whom had EMA-positive cells in the marrow, as did one/10 patients thought to be free of metastases during follow-up. Less than 5 EMA-positive cells were found in the specimens from these patients. This technique may be capable of detecting micrometastatic disease in breast cancer, as well as improving the detection of marrow involvement in patients with disseminated disease. Further studies are being undertaken to determine whether larger quantities of marrow from multiple sites will enable the detection of micrometastatic disease in patients with poor-risk primary breast cancer.

### THE VALUE OF RADIOIMMUNODETECTION IN THE MANAGEMENT OF CHORIOCARCINOMA.

R. H. J. Begent, F. Searle, G. Stanway, R. F. Jewkes, P. Vernon, E. S. Newlands & K. D. Bagshawe, *Depts of Medical Oncology and Nuclear Medicine, Charing Cross Hospital, London, W6 8RF*

There have been a number of recent reports of radioimmunodetection (RID) of cancer, whereby tumours are localized by external scintigraphy after i.v. injection of radiolabelled antibody directed against a tumour product. Images of human tumours producing carcinoembryonic antigen, human chorionic gonadotrophin (hCG) and alpha-foetoprotein have been obtained using the appropriate antibody, but it remains to be shown whether the method is of value in management of patients.

Eighteen patients with hCG-producing tumours (12 gestational choriocarcinoma and 6 malignant teratoma) have been studied by RID, using antibodies directed against hCG. Amongst 13 patients with positive RID were 3 with drug-resistant choriocarcinoma and opacities in the lung fields on computerized tomography. It was not clear whether these opacities were the site of viable tumour or necrotic tissue. RID was definitely positive at the sites of the opacities in 2 patients and suggestive in the third. Deposits of active tumour were subsequently excised from the relevant area of the lung in these patients, and this appears to have contributed to long-term remission in all 3. By contrast, 3 patients with pulmonary opacities on conventional radiology and negative RID do not appear to have active hCG-producing tumour, as judged by serum hCG measurements and in one case by biopsy.

These preliminary results indicate that RID may be of value in management of patients with choriocarcinoma, if it is wished to distinguish between viable and non-viable deposits before surgery.

### MONOCLONAL ANTIBODIES TO HUMAN NEUROBLASTOMA—THEIR POTENTIAL USE IN AUTOLOGOUS MARROW TRANSPLANTATION.

J. T. KEMSHEAD*†, J. PRITCHARD‡, J. S. MALPAS† & M. GREAVES*, **Membrane Immunology Laboratory Imperial Cancer Research Fund, London WC2, †Med. Oncology Lab., St Bartholomew's Hospital, London EC1, ‡Dept of Haematology, Hosp. Sick Children, London*

Advanced neuroblastoma, particularly when bone and/or marrow metastases are present (60+% of cases) has a poor prognosis despite combined treatment with surgery, radiotherapy and conventional-dose combination chemotherapy. In children whose marrows appear by microscopic assessment to be free of tumour cells, we have recently used high-dose (melphalan) chemotherapy with autologous marrow rescue, to see whether remission of disease can be prolonged (Hedley *et al.*, 1979, *Exp. Haematol.*, **7,** 360). Of 12 patients so treated, 3 remain alive and well but 9 have relapsed—mostly at metastatic sites. This pattern of relapse might reflect either failure to control metastatic disease even by high-dose melphalan or re-seeding by residual undetected tumour cells in the reinfused marrow. To investigate this latter possibility, monoclonal antibodies to human neuroblastoma have been produced. A panel of antibodies has been tested against human neuroblastoma cell lines and differential expression of antigens both quantitative and qualitative has been found between cultures. For example, the monoclonal antibody M1/N1 recognizes an antigen expressed in about 4× the quantity on CHP100 cells when compared to CHP212 cells (Kemshead *et al.*, submitted). Similar heterogeneity in the binding of monoclonal antibodies is found on analysis of bone marrow aspirates from children with metastatic neuroblastoma. The biological significance of this antigenic modulation will be discussed and the possibilities for the diagnostic and therapeutic use of these antibodies in the management of patients with neuroblastoma will be assessed.

### USE OF A GLUCOCORTICOID-SENSITIVE HUMAN LYMPHOID CELL LINE TO STUDY INTERACTIONS BETWEEN PREDNISOLONE AND CYTOSINE ARABINOSIDE *IN VITRO*.

M. R. NORMAN & R. GLEDHILL, *Department of Chemical Pathology, King's College Hospital Medical School, London, SE5 8RU*

The established human cell line CCRF CEM, which was originally derived from a patient with acute lymphoblastic leukaemia, was used to isolate a glucocorticoid-sensitive clone, C7 (Norman & Tompson, 1977, *Cancer Res.*, **37,** 3785) and we are now using these cells to simulate the chemotherapy of leukaemia *in vitro*. We have examined the interaction between prednisolone and cytosine arabinoside (Ara-C) according to two protocols: (i) with Ara-C present during the final 24 h of a 48 h exposure of cells to prednisolone, and (ii) with Ara-C present only during the 24 h *prior to* a 48 h treatment with prednisolone. At a concentration of $10^{-8}$M, Ara-C had no measurable effect on lymphoblast cloning efficiency in agarose gels, but nevertheless in protocol (i) it reduced the number of cells killed by prednisolone (0·5 to $5 \times 10^{-6}$M). In protocol (ii) the same concentration of Ara-C increased cell killing by prednisolone ($10^{-6}$M) at 24, 48 and 72 h. These results show that the timing of steroid administration may be crucial when used in conjunction with other drugs.

### TUMOUR OSTEOLYSIS: RESPONSE TO DIPHOSPHONATES AND PROSTAGLANDIN INHIBITORS.

A. W. Samuel, C. S. B. Galasko, S. Rushton & E. Lacey, *Department of Orthopaedic Surgery, University of Manchester, Hope Hospital, Salford*

Previous studies (Galasko & Bennett, 1976, *Nature*, **263**, 508) have shown that prostaglandin-synthesis inhibitor indomethacin can reduce rabbit VX2 carcinoma prostaglandin activity, osteoclast proliferation and osteolysis. Twenty-seven human mammary carcinomas and 20 VX2 carcinomas were cultured against neonatal mouse calvarias, tumour osteolysis being calculated by measuring calcium release. Anti-inflammatory drugs indomethacin, Ibuprofen and Flurbiprofen and the diphosphonates, ethane hydroxy diphosphonate and dichloromethylene diphosphonate, which affect bone resorption directly, were added to the culture medium. Significant reductions in tumour osteolysis were obtained for all drugs ($P<0{\cdot}001$) and VX2 carcinoma (*t* test) whereas the diphosphonates and indomethacin were the most effective drugs with mammary carcinoma, ($P<0{\cdot}001$). The diphosphonates would seem to be as effective as the most effective anti-inflammatory drug in reducing tumour osteolysis, suggesting these agents be evaluated as adjuvant therapy in "early" mammary carcinoma when osteoclastic proliferation would be most marked.

### EFFECT OF 4-HYDROXYANISOLE 4HOA ON MELANOMA.

D. J. T. Webster, R. H. Whitehead & L. E. Hughes, *Department of Surgery, Welsh National School of Medicine, Cardiff*

It has been suggested that substituted phenols might be effective cytotoxic agents in malignant melanoma. 4-Hydroxyanisole (4HOA) is cytotoxic to cultured melanoma cells at $10^{-4}$M. Such tissue levels can safely be obtained in mice, as the required dose is well below the $LD_{50}$ (400 mg/kg). We have therefore studied its effect on transplanted B16 melanoma in mice, and subsequently on patients with advanced malignant melanoma. Male C57 BL mice were inoculated with $5\times10^5$ B16 melanoma cells, into the right thigh, on Day 0. They were randomly allocated to one of 3 groups and were treated by the appropriate protocol: Group I, 0·1 ml saline i.p. daily (control group); Group II, 2·5 mg 4HOA in 0·1 ml i.p. daily (prophylactic group); Group III, 0·1 ml saline i.p. daily until Day 8, then 2·5 mg 4HOA daily (treatment group). Prophylactic use of 4HOA delayed the appearance of tumours (7 days *vs* 9 days, $P=0{\cdot}02$ Wilcoxon rank-sum test) but all animals developed tumours. At sacrifice on Day 15 there were no significant differences in tumour weights: Group I, $2{\cdot}06\pm0{\cdot}73$ g; Group II, $1{\cdot}71\pm0{\cdot}64$ g; Group III, $1{\cdot}92\pm0{\cdot}97$ g. Subsequent pilot studies in patients with advanced malignant melanoma have been started. Twelve/21 cutaneous nodules of malignant melanoma directly injected with 12·5 mg 4HOA underwent temporary regression. I.v. 4HOA was well tolerated in 6 patients up to doses of 1·5 g and total doses of 7·5 g. Nausea was a frequent complaint and in one patient a transient fall in peripheral leucocytes was seen. No serious side effects were seen, and one patient had a brief partial response. Although these results are not very encouraging, further assessment of this agent using different protocols may be justified.

### THE ENHANCEMENT OF EFFECT OF CHEMOTHERAPEUTIC DRUGS BY RADIOSENSITISERS:

W. M. C. Martin & N. J. McNally, *Gray Laboratory of the Cancer Research Campaign, Mount Vernon Hospital, Northwood, Middlesex*

Cells from s.c. tumours of the culture-adapted sarcoma, WHFIB, were found to exhibit repair of potentially lethal damage (PLD) after treatment by cyclophosphamide (CY). Misonidazole (MISO) given 1 h prior to CY inhibited this PLD repair and caused potentiation of CY cytotoxicity, which was more marked at 24 h than 4 h after CY injection. 1 mg/g MISO caused a dose-modifying factor (DMF) of 2·1 with this system. Comparable effects were also found using the regrowth-delay assay. Other 2-nitroimidazole radiosensitizers also potentiated the action of CY.

MISO given 6 h before CY, simultaneously, or even 2 h after, caused significant potentiation of cytotoxicity, but MISO given 1 h before CY produced the optimal effect. There was a linear relationship between sensitizer dose and the regrowth delay following combined treatment. With melphalan, 1 mg/g MISO caused a DMF of 2·5. 2 mm WHFIB lung tumours did not exhibit repair of PLD after CY and also showed less potentiation of CY cytotoxicity by MISO than s.c. tumours. Normal-tissue effects have been encouraging: the CY-induced $LD_{50/60}$ was altered by less than 10% by combination with 1 mg/g MISO; and 1 mg/g MISO caused no significant increase in leucopaenia with therapeutic doses of CY.

**_IN VITRO_ RADIATION SURVIVAL CURVES FOR SOME HUMAN TUMOURS NOT PREVIOUSLY ADAPTED TO CULTURE.** V. D. Courtenay & J. Mills, *Radiotherapy Department, Institute of Cancer Research, Sutton, Surrey*

The response to $\gamma$-radiation was measured in 8 different human tumours, using a replenishable soft-agar-colony technique described by Courtenay & Mills (1978, *Br. J. Cancer*, **37**, 261). The tumours examined were 3 melanomas, 3 ovarian carcinomas and 2 pancreatic adenocarcinomas. They were grown as xenografts in immune-suppressed CBA mice and maintained in serial passage, with the exception of one of the ovarian tumours, which was taken directly from the patient in the form of ascites. Single-cell suspensions prepared from the tumours were exposed to $\gamma$-rays from a $^{60}Co$ source under oxygenated conditions.

The shapes of the survival curves were similar within each tumour category, but were dissimilar for different categories of tumour, particularly in the shoulder region. Average radiation parameters calculated by linear-regression analysis for the melanomas, ovarian and pancreatic tumours, respectively, were $D_0 = 1{\cdot}4$, 0·88 and 1·1 Gy and $D_q = 3{\cdot}0$, 2·5 and 0·4 Gy. If these measurements reflect the radiosensitivity of the original tumour in the patient, they could explain the poor response to fractionated radiotherapy of certain types of tumours, particularly melanomas, treated with small dose fractions.

**MEASUREMENT OF THE UPTAKE OF DAUNORUBICIN BY FLOW MICROFLUORIMETRY.** A. T. McGown & B. W. Fox, *Paterson Laboratories, Christie Hospital and Holt Radium Institute, Manchester M20 9BU*

Flow microfluorimetry offers a rapid and sensitive technique for the investigation of cellular Daunorubicin (DnR) (NSC-82151) incorporation. The intrinsic fluorescence of DnR gives a direct measure of the intracellular drug concentration.

The rate and extent of drug incorporation into P388 murine lymphoma cells in culture is dependent on several parameters. The process of drug accumulation is temperature dependent, an increase in uptake accompanying a rise in incubation temperature. Variation of the pH of the culture medium indicates that the drug is taken up in its deprotonated form ($pK_a = 8{\cdot}25$, Dano, 1973, *Biochim. Biophys. Acta*, **323**, 466). The rate and extent of uptake is also proportional to the concentration of drug in the incubation medium.

The presence of serum lowers the total drug incorporation. Albumin, a major componen t of serum, shows a similar effect. However, no evidence for DnR-albumin interactions has been found by optical or fluorimetric methods.

The metabolic inhibitors 2-deoxyglucose and azide have no effect on the drug accumulation.

Recent improvements in the sensitivity of flow microfluorimetry (Keene & Hodgson, *Cytology* (in press)) allow us to observe the cells. However, no pattern is observed in living cells.

**COMPARATIVE KINETIC EVALUATION OF THE METABOLIC N-DEMETHYLATION OF HEXAMETHYLMELAMINE (HMM) AND ITS METABOLITES _IN VITRO_.** C. J. Brindley & A. Gescher, *Cancer Chemotherapy Research Group, Department of Pharmacy, University of Aston in Birmingham*

Experimental antitumour tests and results from clinical trials indicate that none of the N-demethylated metabolites of HMM is more effective or less toxic than the parent drug. Yet there is strong evidence that HMM

itself is not cytotoxic and requires metabolic activation (A. E. Bateman *et al.*, 1979, *Br. J. Cancer*, **40,** 81). The N-methyl groups in the HMM molecule are essential for antineoplastic activity and undergo extensive metabolic oxidation *in vitro* and *in vivo*. We characterized the enzyme system which catalyses the N-demethylation of HMM and its metabolites, to elucidate the relationship between biotransformation and differences in cytotoxicities of these agents, and to explain the pharmacokinetic profiles of HMM metabolites after HMM administration (M. Broggini *et al.*, *Cancer Treat. Rep.*, in press). The following apparent $K_M$ values were calculated by determining colorimetrically the total amount of formaldehyde and formaldehyde precursors produced during the incubation of drugs with mouse liver microsomes:

| Compound | $R_1$ | $R_2$ | $R_3$ | $R_4$ | app. $K_M$ (mM) |
|---|---|---|---|---|---|
| HMM | $CH_3$ | $CH_3$ | $CH_3$ | $CH_3$ | $0{\cdot}09 \pm 0{\cdot}01$ |
| PMM | $CH_3$ | $CH_3$ | $CH_3$ | H | $0{\cdot}23 \pm 0{\cdot}08$ |
| TMM | $CH_3$ | $CH_3$ | H | H | $0{\cdot}21 \pm 0{\cdot}03$ |
| TetraMM | $CH_3$ | H | H | $CH_3$ | $0{\cdot}91 \pm 0{\cdot}07$ |
| TriMM | H | H | H | $CH_3$ | $1{\cdot}7 \pm 0{\cdot}6$ |

The affinity of these compounds for the mixed-function oxygenases appears to be related to the number of N-methyl moieties within the molecules, and the long plasma half-lives of TetraMM and TriMM after HMM administration may be associated with their low affinities for the metabolizing enzymes.

### COMPARATIVE PHARMACOLOGY OF PENTAMETHYLMELAMINE IN MAN, RAT AND MOUSE.

C. J. Rutty, D. R. Newell, J. F. R. Muindi & K. R. Harrap, *Department of Biochemical Pharmacology, Institute of Cancer Research, Sutton, Surrey*

Despite marked activity against certain experimental mouse tumours, and in particular a number of human tumour xenografts, phase I clinical studies with pentamethylmelamine (PMM) have failed to demonstrate any complete or partial responses in man (1980, *Proc. Am. Assoc. Cancer Res.*, **21,** 136, 143, 178, 347). A pharmacological rationale has been sought to explain this apparent discrepancy. Oxidative N-demethylation of PMM to lower methylmelamines has been demonstrated in man, rat and mouse. This proceeds via N-methylolmelamines, which are thought to be responsible for the antitumour activity (1980, *Chem. Biol. Interact.*, **29,** 235). PMM and its N-demethylated metabolites were measured in plasma by HPLC, and N-methylolmelamines by a micro-Nash technique. In the mouse PMM was metabolized rapidly and extensively to N-methylolmelamines ($t_{\frac{1}{2}} \sim 12$ min). However, in the rat PMM metabolism was significantly slower ($t_{\frac{1}{2}} = 38$ min) and the levels of N-methylolmelamines correspondingly lower. In man the rate of PMM metabolism was considerably slower ($t_{\frac{1}{2}} = 108$ min) and N-methylolmelamines were not detectable. These metabolic differences may explain the sensitivity of certain mouse tumours, and human tumours grown in the mouse, and the lack of clinical response to PMM. The direct administration of N-methylolmelamines would circumvent the need for metabolic activation, and might thereby lead to improved therapeutic efficacy.

### STRUCTURALLY DIVERSE HEPATOCARCINOGENS *IN VIVO* INDUCE DRUG RESISTANCE IN RAT HEPATOCYTES WHEN TESTED *IN VITRO*.

B. I. Carr, *Department of Medical Oncology, City of Hope National Medical Center, Duarte, California* 91010, *U.S.A.*

Carcinogen-altered cells have generally been shown to be more resistant to the toxic effects of carcinogens than normal cells (review: Diamond, 1969, *Prog. Exp. Tumor Res.*, **11,** 364) as predicted by Haddow (1938, *Acta Unio Int. Contra Cancer*, **3,** 342). It was therefore reasoned that the marked resistance of human hepatocellular carcinoma to cytotoxic chemotherapy might also be due to a carcinogen-induced phenotypic change in hepatocytes, resulting in an increased resistance to both carcinogen- and chemotherapy-mediated cytotoxicity.

Male F344 rats were fed a basal diet and given aflatoxin $B_1$ ($AFB_1$) 30 $\mu$g daily i.p., or a basal diet containing either ethionine

0·25% (w/w), or 3′-methyl-4-dimethylaminoazobenzene (0·06% (w/w). Rats were sacrificed weekly and their hepatocytes were placed in primary monolayer culture. Following a 3h attachment, hepatocytes were exposed for 24 h to Adriamycin ($1{\cdot}8 \times 10^{-4}$M) cycloheximide ($7 \times 10^{-3}$M) or $AFB_1$ ($10^{-6}$M). Resistance was defined as the percentage of attached cells excluding trypan blue compared to untreated controls. Each of the 3 carcinogens induced >50% resistance to $AFB_1$ in the isolated hepatocytes after one week, and comparable resistance to cycloheximide and Adriamycin after 4 weeks. Hepatocytes from normal rats did not survive these concentrations of test drugs (Carr, 1980, *Proc. Am. Assoc. Cancer Res.*, **21**, 110).

*In vivo*, 3 different hepatocarcinogens induced marked resistance in rat hepatocytes to the potent carcinogen $AFB_1$, as well as to the cytotoxic drug Adriamycin, using an *in vitro* assay, several months before the appearance of hepatocellular carcinoma. Clinical drug resistance may be an early carcinogen-induced phenotype in primary tumours.

**INTERACTION OF PLATINUM CO-ORDINATION COMPLEXES WITH THE CONSTITUENTS IN CULTURE MEDIA AND THE RELEVANCE TO THEIR REACTIONS *IN VIVO*.** M. LAVERICK & A. H. W. NIAS, *Richard Dimbleby Department of Cancer Research, St Thomas's Hospital Medical School, London, SE1 and P. J. Sadler and I. M. Ismail, Chemistry Dept, Birkbeck College, London, WC1E*

Defined culture media have been devised, which provide optimal conditions for cell proliferation and a high plating efficiency. Media suitable for biophysical studies, such as radiobiology, may contain constituents which cause artefacts in biochemical studies, such as cancer chemotherapy. Previous experiments with the medium Ham's F12 and the platinum II co-ordination complex cis-PAD showed a marked change in cytotoxicity after a small change in the constituents of the medium (Szumiel & Nias, 1976, *Chem-Biol Interact*, **14**, 217). The effect of the complex CHIP (*cis*-dichloro-bis (isopropylamine) *trans* dihydroxy platinum IV) on Chinese hamster ovary (CHO) cells has been found to be markedly influenced by the culture medium. The cytotoxicity of CHIP is less when cells are grown and treated in Ham's F12 medium than in Minimal Essential Medium (MEM). The biological half life of CHIP measured in Ham's F12 at 37°C over 1–2 h showed a biphasic decay in cytotoxic activity, with half-lives of 30 min and 130 min. Over a similar period at 37°C, no change in cytotoxic effect is seen in MEM or physiological saline. L-cysteine, used at levels similar to those found in Ham's F12 medium, can alone reduce cytotoxicity of CHIP. Specific interactions between CHIP and L-cysteine have been detected on a similar time scale by nuclear-magnetic-resonance spectroscopy and spectrophotometry. The interaction of this drug with the culture medium could also mask or alter the potentiating effect with radiation *in vitro*. The choice of media for these studies must therefore be decided on the basis of the clinical environment in which cancer cells are expected to be growing and would be treated.

**THE ROLE OF DNA EXCISION REPAIR IN THE RECOVERY OF HUMAN CELLS FROM CISPLATIN TOXICITY.** M. F. PERA* & J. J. ROBERTS, *Institute of Cancer Research, Royal Cancer Hospital, Pollards Wood Research Station, Chalfont St Giles, Bucks. HP8 4SP*

* Supported by NCI Fellowship F32CA06525-01.

We evaluated the capacity of human cells to recover from the cytotoxic effects of the antitumour agent cisplatin, and we studied the relationship between biological recovery and DNA excision repair. Normal human fibroblasts were grown to confluence and treated with cisplatin. The treated cells were then plated immediately, or they were held in the density-inhibited, non-dividing state for various times after treatment. Cells plated immediately after a 1h exposure to 40 $\mu$M cisplatin showed a survival, measured by colony-forming efficiency, that was 0·21–1·0% of control values. If the cells were held in a density-inhibited state after treatment, no DNA synthesis occurred and survival increased during a 6-day holding period to levels ~15–40% of controls. During the holding period, nondividing cells removed platinum residues from their DNA by a first-order process with a half-life of 2·5 days. The relationship between the logarithm of cell survival and binding of platinum to DNA was determined for cultures plated immediately after drug treatment, and it was found to be similar to that observed for cells that were held in the non-dividing state and allowed to perform DNA repair prior to plating and entry into the proliferative cycle. DNA–DNA interstrand crosslinks, measured by determination of the renaturable fraction of DNA in cell lysates, appeared to be removed at a rate comparable to that of overall adduct removal. These results show that human cells can recover from the cytotoxic effects of cisplatin, that this biological recovery is related to DNA excision repair and interstrand crosslink removal, and that the determinant of cytotoxicity is the extent of drug-DNA interaction at the time cells undergo DNA synthesis and division.

**ACYLATED DINUCLEOSIDE PHOSPHATE DERIVATIVES AS AGENTS TO OVERCOME RESISTANCE TO PURINE ANTIMETABOLITES.** D. M. Tidd, I. Gibson & P. D. G. Dean,* *Universities of East Anglia and *Liverpool*

Montgomery *et al.* (1963, *Nature*, **199**, 769) reported that a dinucleoside mono-phosphate derivative of 6-mercaptopurine, *bis*(thioinosine)-5′,5‴-phosphate, was capable of inhibiting the growth of 6-mercaptopurine-resistant H.Ep 2 cells in concentrations at which 6-mercaptopurine itself was without effect. This result was not repeated in other cell systems, nor did other antimetabolite dinucleoside monophosphates show any advantage over the parent drugs in terms of their effects on resistant cell sublines. We have synthesized $P^1$, $P^2$-*bis*(6-thioinosine-5′) pyrophosphate and have found that this compound is roughly equivalent to 6-thioinosine in its activity against thiopurine-sensitive and insensitive L1210 cultures. Apparently the derivative was cleaved extracellularly, initially to 6-thioinosine and its diphosphate. In contrast, $P^1$, $P^2$-*bis* (2′,3′-0-dibutyryl-6-thioinosine-5′) pyrophosphate was less active than 6-thioinosine against L1210 cells. However, on a molar basis, this acylated compound was ~85-fold more active than 6-thioinosine against the thiopurine-insensitive subline. Similarly, *bis*($N^2$-butyryl-6-thioguanosine)-5′,5‴-monophosphate was less effective than 6-thioguanosine against thiopurine-sensitive L1210 cells, but ~64-fold more active than 6-thioguanosine against the thiopurine-insensitive cells. These results suggest that the intact acylated compounds were taken up by cells in sufficient quantity to generate inhibitory concentrations of activated drug nucleotide metabolites by intracellular cleavage.

**INTERACTION OF VP 16–213 WITH DNA REPAIR ANTAGONISTS.** A. M. Arnold & J. M. A. Whitehouse, *CRC Medical Oncology Unit, University of Southampton*

The podophyllotoxin derivative VP 16–213 can apparently cause single-stranded breaks in DNA, capable of repair. Several compounds are known to be powerful inhibitors of DNA repair, so it was of interest to study the interactions of VP 16–213 with chloroquine and caffeine. Female DBA mice weighing 20 g were inoculated s.c. with $10^5$ TLX lymphoma cells. Three days after inoculation the animals were treated with VP 16-213, together with either chloroquine (20–40 mg/kg) by i.p. injection or caffeine (0·125–1%) added to the drinking water. By way of a control, vincristine, a drug not thought to damage DNA, was also tested with chloroquine. Results were expressed as increased lifespan (ILS) over untreated controls. VP 16-213 alone (6.25 mg/kg and 12.5 mg/kg) produced an ILS of 9% and 30% respectively. VP 16-213 in the same doses but together with 40 mg/kg chloroquine produced ILS of 78% and 76% respectively ($P=0{\cdot}05$). By contrast chloroquine did not enhance the effect of vincristine. One per cent caffeine added to drinking water of animals given VP 16-213 produced a significant but less marked ILS. These preliminary observations provide further evidence that the mode of action of VP 16-213 may be due to the formation of single-stranded breaks in DNA, though it is possible that other mechanisms may be responsible for the apparent synergism.

**ALKALINE ELUTION STUDIES WITH BUSULPHAN AND RELATED DIALKANESULPHONATES.** P. Bedford & & B. W. Fox, *Paterson Laboratories, Christie Hospital and Holt Radium Institute, Manchester M20 9BX*

The DNA-DNA and DNA-protein cross-linking produced by two members of the anti-tumour dialkane sulphonic acid esters of general structure:

$$CH_3SO_2O(CH_2)_nOSO_2CH_3$$

have been studied, using the technique of alkaline elution (Kohn *et al.*, 1976, *Biochemistry*, **15**, 4629). The two agents, methylene dimethanesulphonate (MDMS, n = 1) and 1,4-dimethylsulphonyl-oxybutane (busulphan, n = 4) have been compared in this study. Both agents were found to produce DNA-DNA interstrand crosslinks in cells derived from the transplantable Yoshida rodent sarcoma. In addition, a substantial number of DNA-protein crosslinks and a small number of single-strand breaks were observed after MDMS treatment. The DNA-protein component of crosslinking was entirely attributed to the formaldehyde produced on hydrolysis of the drug. The approximate relative occurrence of the observed lesions as a percentage of total damage after MDMS was calculated as 26%, 64% and 10% respectively for DNA-DNA cross-links, DNA-protein cross-links and single-strand breaks. At doses producing 50% cell death in the cross-sensitive YS line, MDMS was found to induce 0·103 cross-links/$10^9$ dalton compared with 0·151 cross-links/$10^9$ dalton after an equitoxic dose of busulphan. Initial temporal studies with each agent, using a cell line resistant to MDMS ($YR_8$) and resistant to busulphan ($Y_{BUS}R$) compared with the wild-type YS line, suggested that a saturation of excision repair of the cross-links could occur at high doses of each agent in the sensitive line, whereas this was not the case at equivalent doses in the resistant lines.

### THE COMPARISON OF THREE ASSAYS FOR THE DETERMINATION OF *IN VITRO* CHEMOSENSITIVITY USING HUMAN CELL LINES.

A. P. Wilson, C. E. Newman, C. H. J. Ford & A. Howell, *Clinical Oncology Unit, University of Birmingham, Queen Elizabeth Hospital, Birmingham*

The chemosensitivities of 3 human cell lines (T13; transformed fibroblasts: MCF-7; breast ca: Calu 6; lung ca) to Adriamycin (ADM), Bleomycin (BLM), *cis*-platinum (CIS), cyclophosphamide - phosphoramide mustard (CYM), 5-fluorouracil (FU) and cytosine arabinoside (CYT) have been determined, using a biochemical assay (Kauffman *et al.*, 1978, *Strahlentherapie*, **154**, 277) a clonogenic assay (Salmon *et al.*, 1978, *N. Engl. J. Med.*, **298**, 1321) and a monolayer assay (Wilson, 1978, *Clin. Sci. Mol. Med.*, **55**, 16P). The end points of the assays were depressions of nucleotide incorporation, colony formation and leucine incorporation respectively. All drugs were tested at $10^{-2}$, $10^{-3}$ and $10^{-4}$ mg/ml. Cell lines were classified as sensitive (>60% inhibition), resistant (<40% inhibition) or intermediate (40–60% inhibition).

With ADM, all 3 assays showed good correlation with sensitivity and resistance, but for the other drugs the biochemical test was the least sensitive of the assays and correlated only for resistance. Calu 6 and MCF-7 were resistant to CYM in all assays; T13 was sensitive in the monolayer assay only. The monolayer and clonogenic systems correlated well at $10^{-2}$ and $10^{-4}$ mg/ml, but there was disparity at $10^{-3}$ mg/ml. Reduction of drug exposure time in the monolayer assay from 48 h to 3 h gave absolute correlation between the two assays.

These comparisons were performed as a preliminary to comparing results using different assays on primary human tumours for the purpose of predictive chemosensitivity testing for the individual. The results indicate that the biochemical system is suitable for testing the sensitivity of cells to ADM, and that the clonogenic and monolayer assays give similar results, particularly if the drug-exposure time of the monolayer assay is reduced.

### EVALUATION OF THE *IN VITRO* RESPONSE OF HUMAN GLIOMAS TO CHEMOTHERAPEUTIC AGENTS AND ITS CORRELATION WITH CLINICAL RESPONSE.

J. L. Darling & D. G. T. Thomas, *Department of Neurological Surgery, Institute of Neurology, London, WC1N 3BG*

It is well recognized that histopathologically similar malignant gliomas do not respond uniformly to single anti-neoplastic agents. Recent experimental data suggests that this may be due to a cellular heterogeneity in response to chemotherapeutic agents. Using a technique based on scintillation autofluorescence, we have measured the response of 36 malignant gliomas *in vitro*. Each tumour was transferred into monolayer culture and tested separately against a panel of 3 chemotherapeutic agents: vincristine, CCNU and procarbazine. Response, as measured by inhibition of protein synthesis, was determined at a range of drug concentrations, following both short-term recovery (4–24 h) and long-term recovery (4–12 days). Among the tumours tested, a 1·2–6·7 log variation in response was noted for each of the drugs studied.

It was possible to correlate clinical data with the *in vitro* data in 6 patients. Of 3 patients that clinically responded, 2 responded *in vitro* to all 3 drugs and one responded to 2 of the 3 drugs. Of 3 patients who failed to respond clinically, only one had a significant responses *in vitro* to a drug.

These data demonstrate a wide variation in response *in vitro* among histologically similar tumours to single chemotherapeutic agents. The true significance of this is not known, but in preliminary analysis a close correlation is noted between *in vitro* response and clinical response.

**EVALUATION OF THE DRUG SENSITIVITIES OF HUMAN NEUROBLASTOMA CELLS *IN VITRO*.** B. T. Hill & R. D. H. Whelan, *Laboratory of Cellular Chemotherapy, Imperial Cancer Research Fund Laboratories, London, WC2 3PX*

The dismal prognosis for the majority of children with neuroblastoma highlights the need for more effective therapies. One approach is to screen experimentally for drugs active in this disease. The patterns of survival of human neuroblastoma (CHP100) cells were established following a 24 h *in vitro* exposure to a range by colony formation in agar. The dose–response curves were of two types, exponential-plateau or exponential. The former category included: hydroxyurea, methotrexate, ICRF 159, vincristine, vindesine, VM-26 and VP-16-213. Exponential curves were seen with actinomycin D, adriamycin, bleomycin, cis-platinum, dibromodulcitol, 5-fluorouracil, m-AMSA, melphalan and peptichemio. A comparison was attempted between those drug concentrations required to reduce survival by 70% under these conditions, with peak plasma levels achievable in man. The results obtained, after many and varied assumptions, allowed a division of the drugs tested into 3 groups: (i) most effective agents—m-AMSA, VM-26 and adriamycin, (ii) agents with some activity—vincristine, vindesine, VP-16-213, cis-platinum, melphalan and peptichemio, although only for higher doses of the latter 3 drugs, (iii) agents with little, if any, activity—actinomycin D, bleomycin, dibromodulcitol, 5-fluorouracil, hydroxyurea and ICRF 159. Clinical data suggest value in treating neuroblastoma with VM-26 and Adriamycin (in our Group (i)) and with vincristine, melphalan (high dose), peptichemio and cis-platinum (in our Group (ii)). No activity has been reported for actinomycin D or bleomycin (in our Group (iii)). Our experimental results suggest that clinical evaluation of m-AMSA and VP-16-213 might be profitable.